# Practical and economic lithiations of functionalized arenes and heteroarenes using Cy_2_NLi in the presence of Mg, Zn or La halides in a continuous flow†
†Electronic supplementary information (ESI) available. See DOI: 10.1039/c5sc02558c
‡
‡We thank the SFB 749 (DFG) for support and financial contributions to this project. We also thank Rockwood Lithium GmbH (Frankfurt) and BASF AG (Ludwigshafen) for the generous gift of chemicals.


**DOI:** 10.1039/c5sc02558c

**Published:** 2015-08-10

**Authors:** Matthias R. Becker, Maximilian A. Ganiek, Paul Knochel

**Affiliations:** a Ludwig-Maximilians-Universität München , Department Chemie , Butenandtstrasse 5-13 (Haus F) , 81377 München , Germany . Email: paul.knochel@cup.uni-muenchen.de

## Abstract

The economic amide base lithium dicyclohexylamide (Cy_2_NLi) allows fast and convenient (40 s, 0 °C) *in situ* trapping flow metalations of a broad range of functionalized arenes, heteroarenes and acrylate derivatives in the presence of various metal salts (ZnCl_2_·2LiCl, MgCl_2_, LaCl_3_·2LiCl). The resulting Zn-, Mg- or La-organometallic intermediates are trapped with various electrophiles in high yields. These flow metalations are easily scaled-up without further optimization.

## Abstract 2

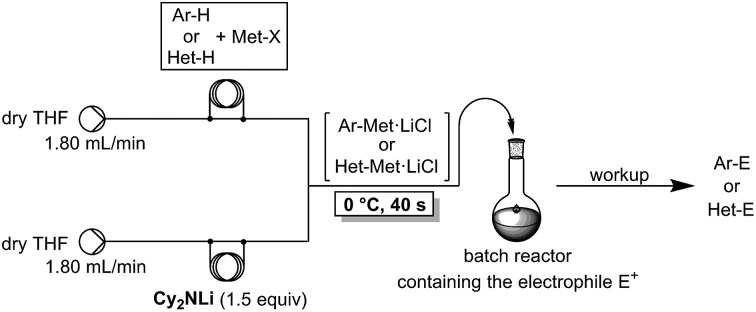
The economic amide base lithium dicyclohexylamide (Cy_2_NLi) undergoes fast (40 s) and convenient (0 °C) *in situ* trapping flow metalations of a broad range of functionalized aromatics in the presence of various metal salts.

## Introduction

The lithiation of arenes and heteroarenes is a common method for the functionalization of unsaturated molecules.^1^ Pioneering work of Snieckus^2^ and others^3^ have demonstrated the utility of aromatic lithiations for the preparation of pharmaceutical and agrochemical targets. Nevertheless, the use of powerful lithium bases has some drawbacks such as low metalation temperatures and a moderate functional group tolerance. Also, it requires a careful choice of the lithium base used for the metalation step.

**Scheme 1:**
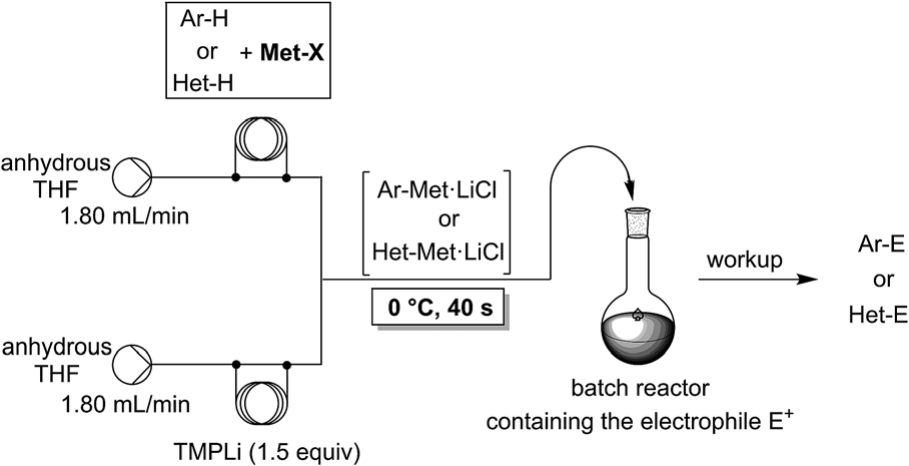
Continuous flow set-up for *in situ* trapping metalations using TMPLi in the presence of metal salts (Met-X = ZnCl_2_·2LiCl, MgCl_2_, CuCN·2LiCl, LaCl_3_·2LiCl) and subsequent batch quenching with electrophiles (E^+^).

Recently, we have shown that an *in situ* trapping metalation sequence using TMPLi (TMP = 2,2,6,6-tetramethylpiperidyl) allows the performance of selective lithiations of various arenes and heteroarenes at 0 °C within 40 s if conducted in a continuous flow system (Scheme 1).^4^,^5^ Under conventional batch conditions, these *in situ* trapping metalations require cryogenic temperatures (–78 °C) in order to avoid unwanted side reactions or decomposition of the organometallic intermediate. Furthermore, the scale-up of these batch metalations proved to be difficult, requiring much optimization. Despite the convenient reaction conditions in flow mode, the use of stoichiometric amounts of TMPLi makes this lithiation still expensive (TMPH = *ca.* 630 $ mol^–1^).^6^ The steric hindrance of the TMP-moiety was required in order to avoid side-reactions. Due to the fast mixing of the reaction components and the prevention of hot spot formation,^7^ such highly sterically hindered bases may no longer be mandatory when using the flow methodology.^8^ Preliminary experiments attempting to perform metalations of various aromatics using cheaper readily available lithium or other metallic amides R_2_NMet (R = iPr (isopropyl), Cy (cyclohexyl), TMS (trimethylsilyl); Met = Li, MgHal, ZnHal) were disappointing either due to insufficient reactivity or unwanted side-reactions. The *in situ* trapping methodology developed in our laboratory, in which we mix the aromatic substrate with a metallic salt and add TMPLi proves to be compatible with the replacement of TMPLi with much cheaper bases, since this Barbier-type lithiation minimizes the contact time of the lithium base with the aromatic substrate. The replacement of TMPLi by Cy_2_NLi is of special importance since the price of the corresponding amine Cy_2_NH (*ca.* 6.40 $ mol^–1^) is only *ca.* 1% of TMPH.^6^,^9^

Herein we wish to report the use of the economic amide base lithium dicyclohexylamide (Cy_2_NLi) instead of TMPLi for *in situ* trapping metalations under continuous flow conditions. Cy_2_NLi has – to the best of our knowledge – not yet been used for extensive lithiations of functionalized arenes and heteroarenes.^10^

## Results and discussion

**Scheme 2:**
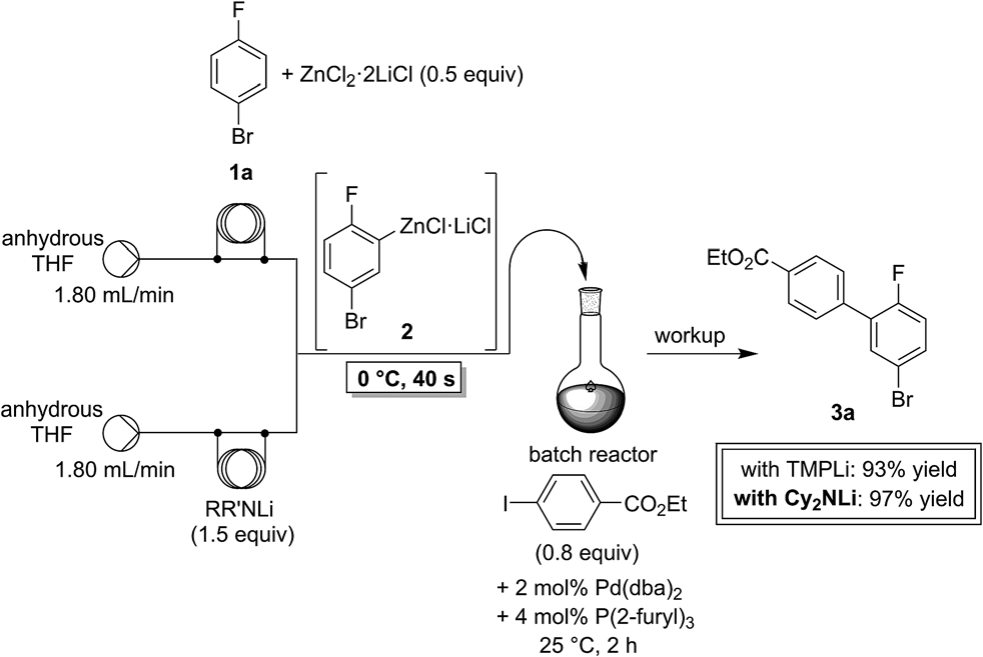
Continuous flow *in situ* trapping zincation of 1-bromo-4-fluorobenzene (**1a**) using TMPLi and Cy_2_NLi and subsequent Pd-catalyzed Negishi cross-coupling with ethyl 4-iodobenzoate in a batch reactor.

In a first experiment, we have metalated 1-bromo-4-fluorobenzene (**1a**) under flow conditions (Scheme 2). Thus, **1a** (1.0 equiv.) was mixed with ZnCl_2_·2LiCl (0.5 equiv.) and submitted to flow metalation^11^ (0 °C, 40 s) using respectively TMPLi and Cy_2_NLi. The corresponding arylzinc intermediate (**2**) was quenched *via* a Pd-catalyzed Negishi cross-coupling^12^ in a batch reactor containing ethyl 4-iodobenzoate (0.8 equiv.) and a standard Pd-catalytic system (2 mol% Pd(dba)_2_; dba = dibenzylideneacetone and 4 mol% P(2-furyl)_3_)^13^ providing the expected biphenyl (**3a**) in 93% (using TMPLi) and 97% (using Cy_2_NLi) yield. Like for reactions with TMPLi, *in situ* trapping metalations with Cy_2_NLi can be simply scaled up without further optimization just by running the reaction for a longer time. Therefore, the reaction of **2** with 3-iodoanisole (0.8 equiv.) affords after a Negishi cross-coupling the expected product **3b** in 97% yield on a 1.7 mmol scale and in 95% yield on a 11 mmol scale (Table 1, entry 1). Using Cy_2_NLi for the *ortho*-lithiation of 1,3-dihaloarenes (**1b**, **c**) abstracts under our standard reaction conditions (0 °C, 40 s) the most acidic hydrogen at position 2. *In situ* transmetalations with ZnCl_2_·2LiCl or MgCl_2_ (0.5 equiv., respectively) generate the corresponding aryl-zinc and -magnesium species, which are quenched in subsequent batch reactions with aryl iodides (0.8 equiv.), *S*-phenyl benzenethiosulfonate (0.8 equiv.) and ethyl cyanoformate (0.8 equiv.) leading to the trisubstituted arenes (**3c–f**) in 67–98% yield (entries 2–5). The *in situ* metalations with Cy_2_NLi are not limited to haloarenes, and sensitive functionalities such as esters and nitriles are tolerated as well. Thus, diethyl 4-bromoisophthalate (**1d**) is smoothly flow-zincated in position 6 and a Negishi cross-coupling with ethyl 4-iodobenzoate (0.8 equiv.) produces the expected triester (**3g**) in 72% yield (entry 6). Similarly, substituted nitriles such as **1e** and **1f** are *in situ* metalated in the presence of ZnCl_2_·2LiCl (0.5 equiv.) within 40 s at 0 °C, and subsequent quenching reactions with aryl iodides (0.8 equiv.) having either electron-donating or electron-withdrawing substituents lead to the cyano-substituted biphenyls (**3h–j**) in 70–97% yield (entries 7–9).

**Table 1:** Continuous flow trapping-metalation of arenes **1** followed by reaction with electrophiles leading to products of type **3**

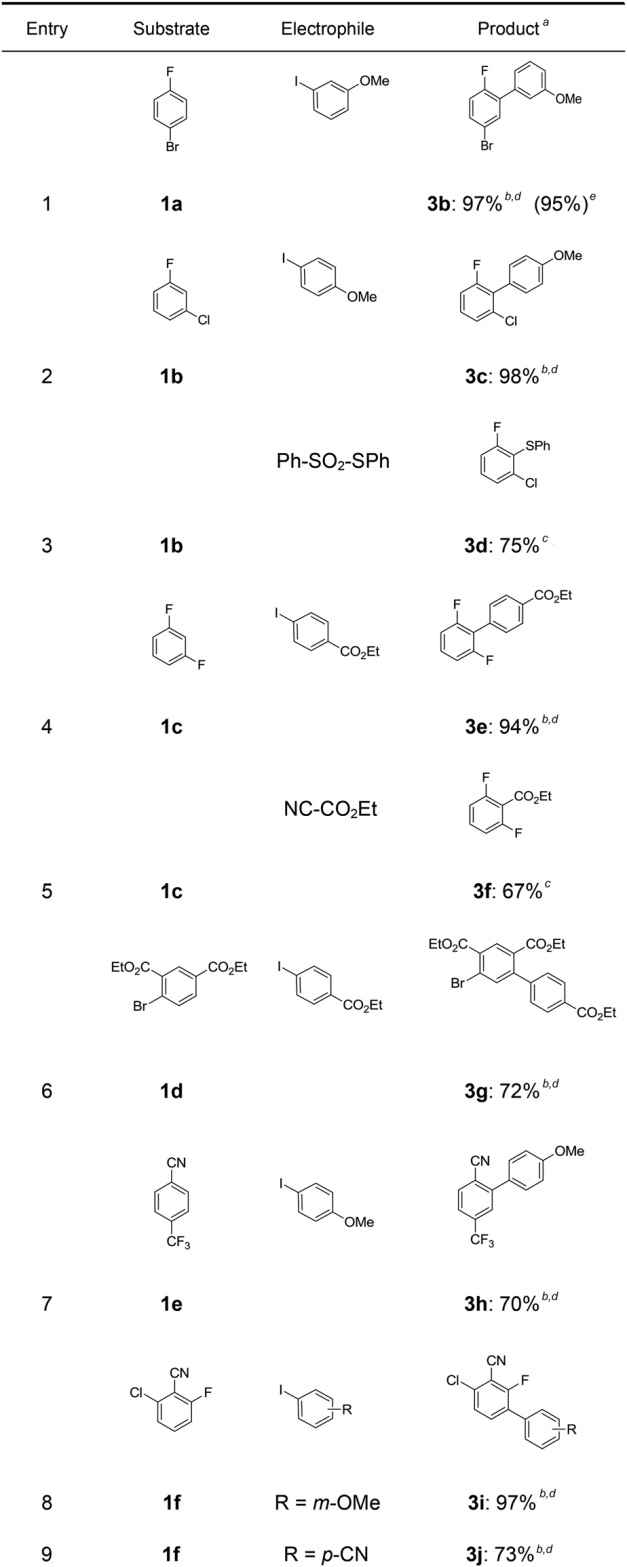

This *in situ* trapping methodology with Cy_2_NLi in a flow reactor is not limited to functionalized arenes. In fact, it can be readily extended to a broad range of sensitive, electron-deficient heteroarenes (Table 2). Thus, 2-fluoropyridine (**4a**), which is notoriously difficult to metalate,^14^ undergoes a smooth zincation or magnesiation in position 3 in the presence of ZnCl_2_·2LiCl or MgCl_2_ and quenching with ethyl 4-iodobenzoate (0.8 equiv.) or *S*-methyl methanethiosulfonate (0.8 equiv.) produces the disubstituted pyridines (**5a**, **b**) in 75–94% yield (entries 1 and 2). However, using our standard conditions, 2,6-dibromopyridine (**4b**) is *in situ* metalated in position 4 and a subsequent Negishi cross-coupling with ethyl 4-iodobenzoate (0.8 equiv.) affords the desired pyridine (**5c**) in 67% yield (entry 3). Similarly, ethyl 2-chloronicotinate (**4c**) is flow-zincated within 40 s at 0 °C in position 4 affording the trisubstituted pyridine (**5d**) in 88% yield after a Cu-mediated allylation with 3-bromocyclohexene (0.8 equiv.; entry 4). The sensitive 2,3-dichloropyrazine (**4d**) is smoothly flow-metalated (0 °C, 40 s) in the presence of ZnCl_2_·2LiCl (0.5 equiv.) and quenching with 3-iodoanisole (0.8 equiv.) leads to the pyrazine (**5e**) in 77% yield (entry 5). The *in situ* trapping metalations with Cy_2_NLi can also be used for the functionalization of a broad range of substituted 5-membered ring heterocycles. Thus, the lanthanation of 1-methylpyrazole (**4e**) in the presence of LaCl_3_·2LiCl (0.5 equiv.) under standard conditions (0 °C, 40 s) produces the desired alcohol (**5f**) in 62% yield after addition to *p*-chlorobenzaldehyde (0.8 equiv.; entry 6). Ethyl 5-bromo-2-furoate (**4f**) is regioselectively flow metalated in position 3 and a subsequent Cu-catalyzed reaction with 3-bromocyclohexene (0.8 equiv.) leads to the trisubstituted furan (**5g**) in 76% yield (entry 7). The *in situ* trapping zincation of 2-bromothiophene (**4g**) within 40 s at 0 °C abstracts the most acidic hydrogen at position 5 affording the 2,5-disubstituted thiophenes (**5h**, **i**) in 89–91% yield after Negishi cross-couplings with 4-iodobenzotrifluoride (0.8 equiv.) and 1-iodo-3-nitrobenzene (0.8 equiv.; entries 8 and 9).

**Table 2:** Continuous flow trapping-metalation of heterocycles **4** followed by reaction with electrophiles leading to products of type **5**

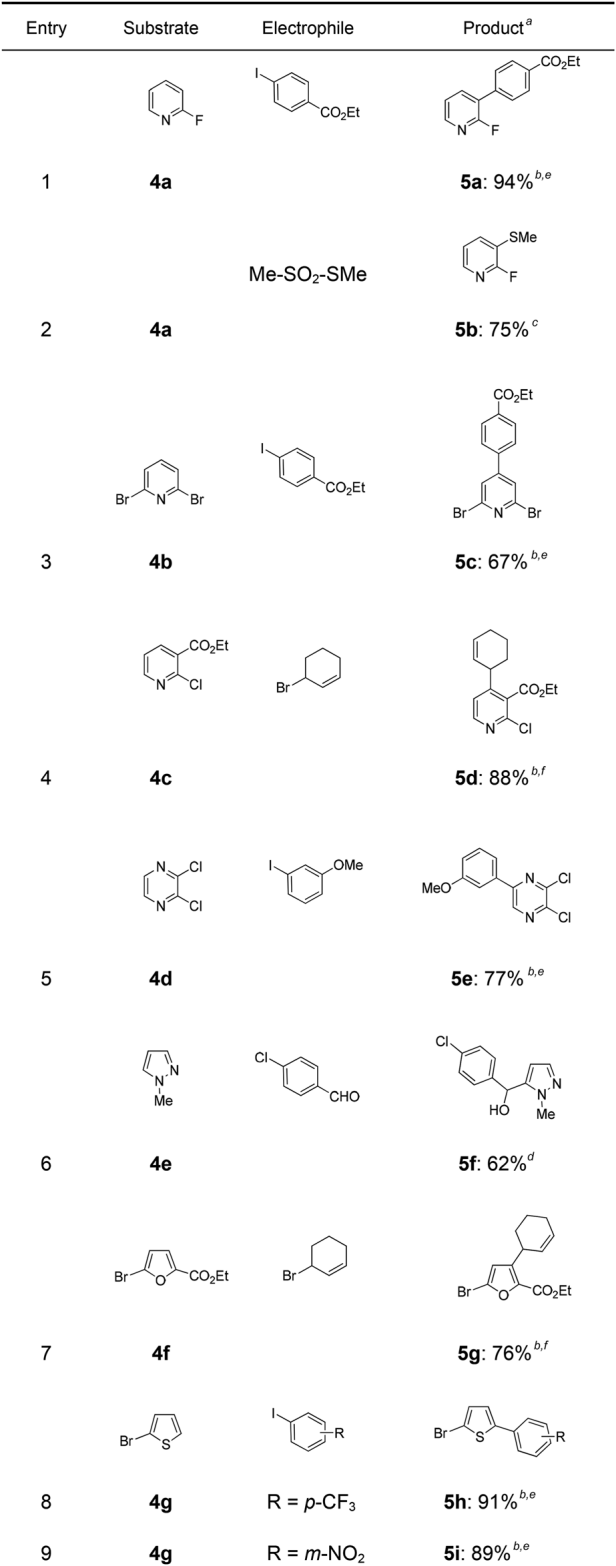

**Scheme 3:**
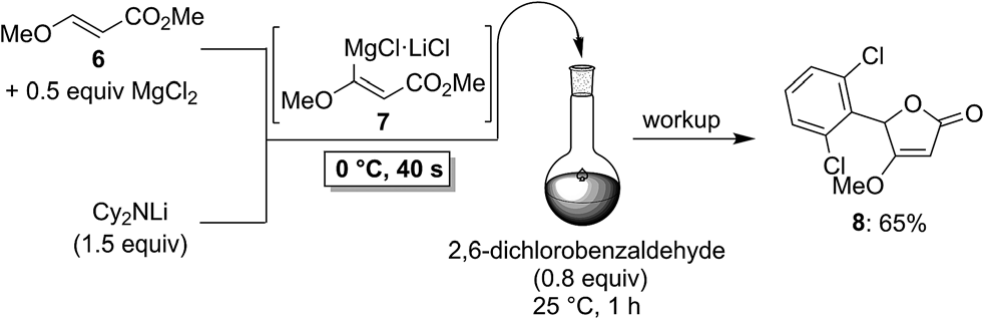
*In situ* trapping magnesiation of (*E*)-methyl 3-methoxyacrylate (**6**) using Cy_2_NLi in a flow reactor.

**Scheme 4:**
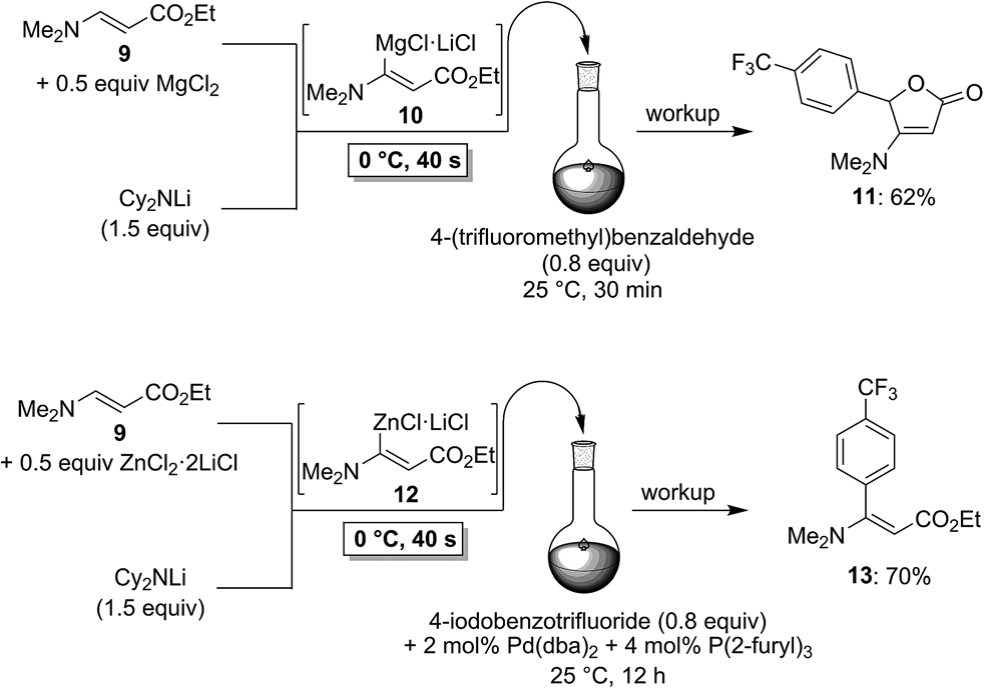
*In situ* trapping magnesiation and zincation of (*E*)-ethyl 3-(dimethylamino)acrylate (**9**) using Cy_2_NLi in a flow reactor.

To demonstrate the broad practicability of the *in situ* trapping metalations with Cy_2_NLi, we investigated the functionalization of acyclic acrylate derivatives, which are prone to polymerize. However, submitting a mixture of (*E*)-methyl 3-methoxyacrylate (**6**) with MgCl_2_ (0.5 equiv.) to the flow metalation with Cy_2_NLi (1.5 equiv.) for 40 s at 0 °C leads to the formation of the magnesium intermediate **7** in high conversion (Scheme 3). Subsequent reaction of **7** with 2,6-dichlorobenzaldehyde (0.8 equiv.) produces the lactone **8** in 65% yield. Similarly, (*E*)-ethyl 3-(dimethylamino)acrylate (**9**) is *in situ* metalated (0 °C, 40 s) in the presence of MgCl_2_ or ZnCl_2_·2LiCl (Scheme 4). The corresponding magnesium (**10**) and zinc (**12**) organometallic intermediates undergo various quenching reactions, such as an addition to 4-(trifluoromethyl)benzaldehyde (0.8 equiv.) and a Negishi cross-coupling with 4-iodobenzotrifluoride (0.8 equiv.), providing the corresponding lactone (**11**) and the ester **13** in 62–70% yield.

## Conclusions

In summary, the economic amide base lithium dicyclohexylamide (Cy_2_NLi) undergoes fast and convenient (40 s, 0 °C) *in situ* trapping flow metalations of a broad range of functionalized arenes, heteroarenes and acrylate derivatives in the presence of various metal salts (ZnCl_2_·2LiCl, MgCl_2_, LaCl_3_·2LiCl). The resulting Zn-, Mg- or La-organometallic intermediates are trapped with numerous electrophiles in high yields. These flow-metalations are easily scaled-up without further optimization simply by running the reaction for a longer time. Further applications and extensions of this method are currently underway.

## Supplementary Material

Supplementary information
