# Supplementary material for: Practical and economic lithiations of functionalized arenes and heteroarenes using Cy2NLi in the presence of Mg, Zn or La halides in a continuous flow
Source: Chem Sci. 2015 Aug 10;6(11):6649–53. doi: 10.1039/c5sc02558c (PMC5802273; doi:10.1039/c5sc02558c)

Electronic Supplementary Material (ESI) for Chemical Science.
This journal is © The Royal Society of Chemistry 2015

# Content

General considerations……………………………………………………..…….2

Typical procedures (TP)……………………………………………………..…...4

Preparation of products…….…………………………………………………..……6

NMR-spectra………………………………………………………..……….28

## General considerations

In situ trapping metalations were carried out with a FlowSyn system purchased from Uniqsis. Metalations were performed in a coiled reactor and a tube (1.0 mm id; 2.0 and 0.4 mL) and reactant solutions were pre-cooled in 1.0 mL loops (1.0 mm id) made from PFA or PTFE Teflon. Carrier solvents as well as reactant solutions were stored under argon. Reactions with electrophiles were carried out under batch conditions with magnetic stirring and in flame-dried glassware under argon. Syringes, which were used to transfer reagents and solvents, were purged with argon prior to use.

### Solvents

Solvents were dried according to standard methods by distillation from drying agents as stated below and were stored under argon. Otherwise they were obtained from commercial sources and used without further purification.

THF was continuously refluxed and freshly distilled from sodium benzophenone ketyl under nitrogen.

Solvents for column chromatography were distilled prior to use.

### Reagents

All reagents were obtained from commercial sources and used without further purification unless otherwise stated.

**$Cy_2NH$** was distilled prior to use and stored under argon.

***n*-BuLi** solution in hexane was purchased from Rockwood Lithium and the concentration was determined by titration against 1,10-phenanthroline in THF with *i*-PrOH.

**CuCN·2LiCl** solution (1.00 M in THF) was prepared by drying CuCN (8.96 g; 100 mmol) and LiCl (8.48 g; 200 mmol) in a Schlenk flask under vacuum for 5 h at 150 °C. After cooling to 25 °C, dry THF (100 mL) was added and stirred until the salts were dissolved.

**$MgCl_2$** solution (0.50 M in THF) was prepared by suspending Mg turnings (2.55 g; 105 mmol) in dry THF (200 mL) in a flame-dried and argon flushed Schlenk flask. Then 1,2-dichloroethane (9.90 g; 100 mmol; 7.92 mL) was carefully added over 15 min and the reaction mixture was stirred overnight at 25 °C until gas evolution was complete.

**$ZnCl_2$·2LiCl** solution (1.00 M in THF) was prepared by drying $ZnCl_2$ (27.3 g; 200 mmol) and LiCl (17.0 g; 400 mmol) in a Schlenk flask under vacuum for 5 h at 150 °C. After cooling to 25 °C, dry THF (200 mL) was added and stirred until the salts were dissolved.
**$Cy_2NLi$** solution in THF was prepared by slow addition of *n*-BuLi (8.2 mL; 20 mmol; 2.5 M in hexane) to a solution of $Cy_2NH$ (3.7 g; 4.1 mL; 20 mmol) in THF (20 mL) at -40 °C and stirred for 30 min at -40 °C. The concentration was determined by titration against *N*-benzylbenzamide in THF.

### Chromatography

Flash column chromatography was performed using $SiO_2$ 60 (0.040-0.063 mm, 230-400 mesh ASTM) from Merck.
Thin layer chromatography (TLC) was performed using aluminium plates covered with $SiO_2$ (Merck 60, F-254). Spots were visualized under UV light and/or by staining of the TLC plate with one of the solutions below, followed by heating with a heat gun:

- $KMnO_4$ (0.3 g), $K_2CO_3$ (20 g) and KOH (0.3 g) in water (300 mL).
- Neat iodine absorbed on silica gel.
- Phosphor molybdic acid (5.0 g), $Ce(SO_4)_2$ (2.0 g) and conc. $H_2SO_4$ (12.0 mL) in water (230 mL).

### Analytical data

NMR spectra were recorded on *Bruker* ARX 200, AC 300, WH 400 or AMX 600 instruments. Chemical shifts are reported as δ-values in ppm relative to the deuterated solvent peak: $CDCl_3$ ($\delta_H$: 7.26; $\delta_C$: 77.16). For the observation of the observed signal multiplicities, the following abbreviations were used: s (singlet), d (doublet), dd (doublet of doublets), t (triplet), q (quartet), sext (sextet), m (multiplet) and br (broad). If not otherwise noted, the coupling constants given are C-H-coupling constants. Melting points are uncorrected and were measured on a *Büchi* B.540 apparatus. Infrared spectra were recorded from 4000-400 $cm^{-1}$ on a *Nicolet* 510 FT-IR or a *Perkin-Elmer* 281 IR spectrometer. Samples were measured neat (*Smiths Detection* DuraSampl *IR* II Diamond ATR). The absorption bands are reported in wavenumbers ($cm^{-1}$). Gas chromatography (GC) was performed with instruments of the type *Hewlett-Packard* 6890 or 5890 Series II, using a column of the type HP 5 (*Hewlett-Packard*, 5% phenylmethylpolysiloxane; length: 10 m, diameter: 0.25 mm, film

thickness: 0.25 µm). The detection was accomplished using a flame ionization detector. Mass spectra (MS) and high resolution mass spectra (HRMS) were recorded on a *Finnigan* MAT95Q or *Finnigan* MAT90 instrument for electron impact ionization (EI) and electrospray ionization (ESI). For the combination of gas chromatography with mass spectroscopic detection, a GC-MS of the type *Hewlett-Packard* 6890 / MSD 5793 networking was used (column: HP 5-MS, *Hewlett-Packard*; 5% phenylmethylpolysiloxane; length: 15 m, diameter 0.25 mm; film thickness: 0.25 µm).

## Typical procedures (TP)

**Typical procedure for the in situ trapping metalation in flow followed by the reaction with an electrophile in batch (TP1):**

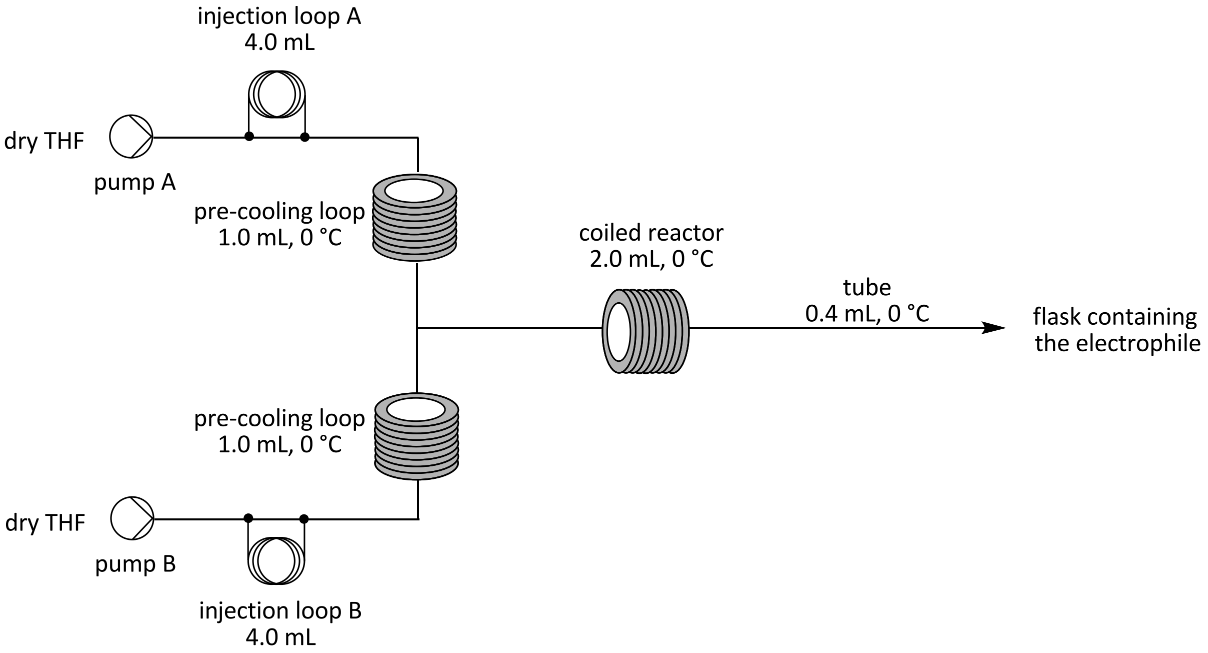


The flow system (FlowSyn, Uniqsis) was dried by flushing it with dry THF (flow rate of all pumps: 1.00 mL/min; run-time: 30 min). Injection loop A (4.0 mL) was loaded with the reactant solution (0.43 – 0.45 M in dry THF containing 0.5 equiv metal salt additive; 5.0 mL) and injection loop B (4.0 mL) was loaded with $Cy_2NLi$ (0.65 – 0.69 M in dry THF; 1.5 equiv; 5.0 mL). The solutions were simultaneously injected into separate THF streams (pump A and B, flow rates: 1.80 mL/min), which passed a pre-cooling loop (1.0 mL; residence time: 33 s; 0 °C) respectively, before they were mixed in a coiled reactor followed by a tube (2.4 mL in total; residence time: 40 s; 0 °C). The combined streams were collected in a flame-dried,

argon flushed 25 mL flask equipped with a magnetic stirrer and a septum containing the electrophile (0.8 equiv) dissolved in dry THF (2 mL). Then the reaction mixture was further stirred for the indicated time at the indicated temperature. The completion of the reaction was checked by GC analysis of reaction aliquots quenched with concentrated aqueous $NH_4Cl$ solution and using undecane as an internal standard.

**Typical procedure for the scale-up in situ trapping metalation in flow followed by the reaction with an electrophile in batch (TP2):**

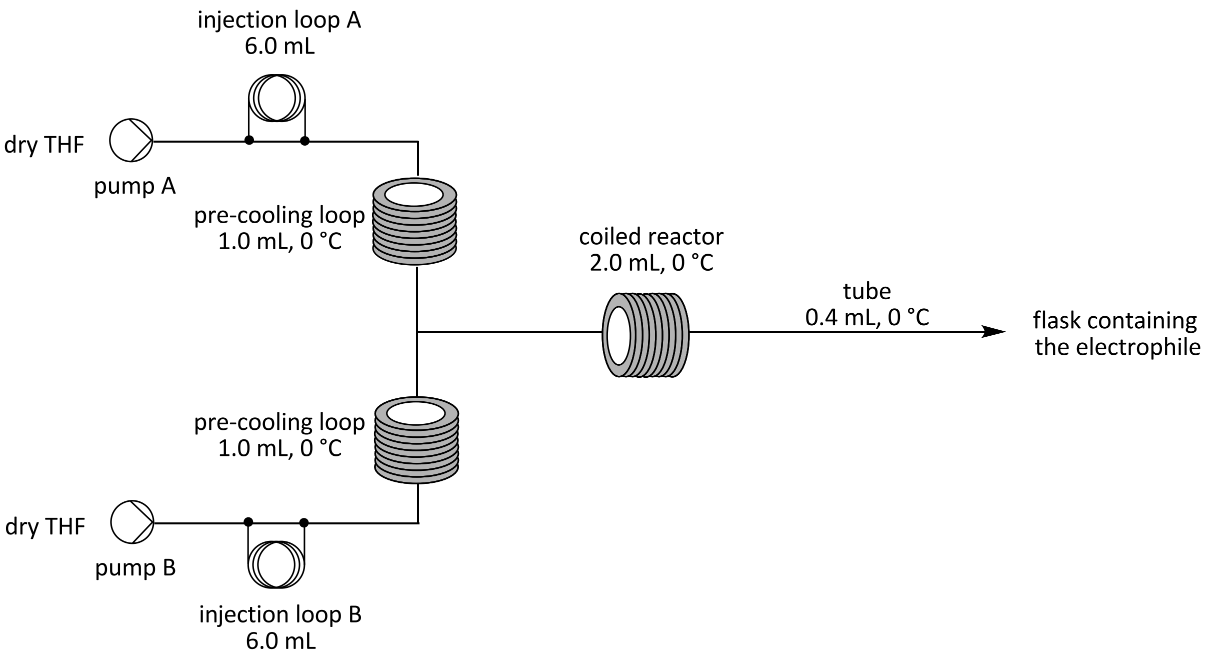


The flow system (FlowSyn, Uniqsis) was dried by flushing it with dry THF (flow rate of all pumps: 1.00 mL/min; run-time: 30 min). Injection loop A (6.0 mL) was loaded with the reactant solution (0.45 M in dry THF containing 0.5 equiv metal salt additive; 7.0 mL) and injection loop B (6.0 mL) was loaded with $Cy_2NLi$ (0.68 M in dry THF; 1.5 equiv; 7.0 mL). The solutions were simultaneously injected into separate THF streams (pump A and B, flow rates: 1.80 mL/min), which passed a pre-cooling loop (1.0 mL; residence time: 33 s; 0 °C) respectively, before they were mixed in a coiled reactor followed by a tube (2.4 mL in total; residence time: 40 s; 0 °C). The combined streams were collected in a flame-dried, argon flushed 250 mL flask equipped with a magnetic stirrer and a septum containing the electrophile (0.8 equiv) dissolved in dry THF (20 mL). After 4.5 min, the injection loops were

reloaded with the reactant solution and $Cy_2NLi$, injected into the separate THF streams again and collected in the same flask as well. The number of reloads was depending on the desired reaction scale. Then the reaction mixture was further stirred for the indicated time at the indicated temperature. The completion of the reaction was checked by GC analysis of reaction aliquots quenched with concentrated aqueous $NH_4Cl$ solution and using undecane as an internal standard.

## Preparation of products

Synthesis of ethyl 5'-bromo-2'-fluorobiphenyl-4-carboxylate (**3a**)

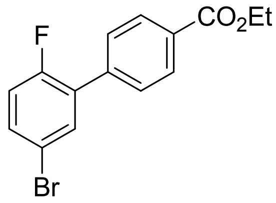


According to **TP1**, injection loop A and B were loaded with solutions of 1-bromo-4-fluorobenzene (**1a**; 0.44 M containing 0.5 equiv $ZnCl_2\cdot 2LiCl$) and $Cy_2NLi$ (0.66 M). After injection and in situ trapping metalation the combined streams were collected in a flask containing ethyl 4-iodobenzoate (389 mg; 1.41 mmol; 0.8 equiv), $Pd(dba)_2$ (20 mg; 2 mol%) and tfp (16 mg; 4 mol%) dissolved in THF (2 mL). The reaction mixture was stirred for further 2 h at 25 °C before it was quenched with water (30 mL). The aq. layer was extracted with EtOAc (3×60 mL), the combined organic fractions were dried over anhydrous $Na_2SO_4$, filtrated and the solvent was removed *in vacuo*. Purification by flash column chromatography (silica gel; *i*-hexane:EtOAc = 40:1) afforded **3a** as a colorless solid (443 mg; 1.37 mmol; 97%).

**m.p.** (°C): 73.8 – 75.7.

**$^1$H-NMR** (300 MHz, $CDCl_3$): $\delta$ / ppm = 8.12 – 8.08 (m, 2H), 7.59 – 7.54 (m, 3H), 7.45 – 7.41 (m, 1H), 7.07 – 7.01 (m, 1H), 4.39 (q, $J$ = 7.1 Hz, 2H), 1.40 (t, $J$ = 7.1 Hz, 3H).

**$^{13}$C-NMR** (75 MHz, $CDCl_3$): $\delta$ / ppm = 166.2, 158.7 (d, $J$ = 249.7 Hz), 138.7 (d, $J$ = 1.5 Hz), 133.2 (d, $J$ = 3.0 Hz), 132.4 (d, $J$ = 8.3 Hz), 130.2, 130.1 (d, $J$ = 15.1 Hz), 129.7, 128.9 (d, $J$ = 3.0 Hz), 118.0 (d, $J$ = 24.1 Hz), 116.9 (d, $J$ = 3.8 Hz), 61.1, 14.3.

**IR** (Diamond-ATR, neat): $\tilde{\nu}$ / cm$^{-1}$ = 3060, 2993, 2978, 2931, 2869, 1885, 1713, 1611, 1576, 1558, 1513, 1473, 1465, 1443, 1415, 1386, 1378, 1366, 1318, 1274, 1254, 1218, 1210, 1185, 1120, 1102, 1080, 1031, 1016, 943, 901, 870, 861, 854, 775, 755, 742, 720, 702, 654.

**MS** (EI, 70 eV): m/z (%) = 324 (32), 322 (32), 296 (23), 294 (24), 279 (62), 278 (12), 277 (65), 171 (15), 170 (100), 169 (15), 85 (33).

**HRMS** (EI): calcd. for [$C_{15}H_{12}BrFO_2$]: 322.0005; found: 321.9999 ($M^+$).

Synthesis of 5-bromo-2-fluoro-3'-methoxybiphenyl (**3b**)

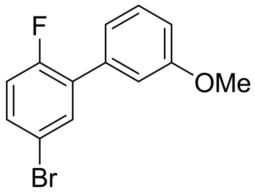


According to **TP1**, injection loop A and B were loaded with solutions of 1-bromo-4-fluorobenzene (**1a**; 0.44 M containing 0.5 equiv $ZnCl_2{\cdot}2LiCl$) and $Cy_2NLi$ (0.66 M). After injection and in situ trapping metalation the combined streams were collected in a flask containing 3-iodoanisole (330 mg; 1.41 mmol; 0.8 equiv), $Pd(dba)_2$ (20 mg; 2 mol%) and tfp (16 mg; 4 mol%) dissolved in THF (2 mL). The reaction mixture was stirred for further 2 h at 25 °C before it was quenched with water (30 mL). The aq. layer was extracted with EtOAc (3×50 mL), the combined organic fractions were dried over anhydrous $Na_2SO_4$, filtrated and the solvent was removed *in vacuo*. Purification by flash column chromatography (silica gel; *i*-hexane:EtOAc = 30:1) afforded **3b** as a yellow oil (384 mg; 1.37 mmol; 97%).

**$^1$H-NMR** (300 MHz, $CDCl_3$): $\delta$ / ppm = 7.58 – 7.55 (m, 1H), 7.40 – 7.37 (m, 1H), 7.35 – 7.32 (m, 1H), 7.11 – 7.07 (m, 1H), 7.05 – 6.99 (m, 2H), 6.95 – 6.91 (m, 1H), 3.84 (s, 3H).

**$^{13}$C-NMR** (75 MHz, $CDCl_3$): $\delta$ / ppm = 159.6, 158.8 (d, $J$ = 248.2 Hz), 135.7 (d, $J$ = 1.5 Hz), 133.4 (d, $J$ = 3.0 Hz), 131.7 (d, $J$ = 8.3 Hz), 131.0 (d, $J$ = 15.1 Hz), 129.6, 121.3 (d, $J$ = 3.0 Hz), 117.9 (d, $J$ = 24.9 Hz), 116.7 (d, $J$ = 3.8 Hz), 114.6 (d, $J$ = 3.0 Hz), 113.8, 55.3.

**IR** (Diamond-ATR, neat): $\tilde{\nu}$ / cm$^{-1}$ = 3068, 3001, 2935, 2835, 1873, 1745, 1599, 1582, 1565, 1495, 1470, 1430, 1389, 1380, 1292, 1263, 1251, 1224, 1205, 1171, 1119, 1076, 1052, 1029, 995, 939, 872, 857, 812, 779, 723, 712, 692.

**MS** (EI, 70 eV): m/z (%) = 281 (38, M$^+$), 252 (31), 239 (38), 237 (45), 186 (22), 172 (25), 171 (25), 170 (61), 169 (12), 158 (62), 157 (100), 152 (12), 140 (12), 138 (13), 132 (20), 85 (20), 75 (10), 74 (12), 63 (14), 50 (11), 43 (11), 41 (11).

**HRMS** (EI): calcd. for [$C_{13}H_{11}BrFO$]: 280.9977; found: 280.9969 (M$^+$ + H).

Scale-up synthesis of 5-bromo-2-fluoro-3'-methoxybiphenyl (**3b**)

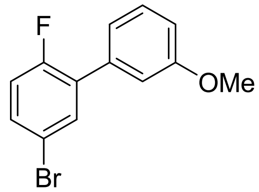


According to **TP2**, injection loop A and B were loaded with solutions of 1-bromo-4-fluorobenzene (**1a**; 0.45 M containing 0.5 equiv $ZnCl_2$·2LiCl) and $Cy_2NLi$ (0.68 M). After injection and in situ trapping metalation the combined streams were collected in a flask containing 3-iodoanisole (2.02 g; 8.64 mmol; 0.8 equiv), $Pd(dba)_2$ (124 mg; 2 mol%) and tfp (100 mg; 4 mol%) dissolved in THF (20 mL). The injection loops were reloaded three times and the reaction mixture was stirred for further 2 h at 25 °C before it was quenched with water (100 mL). The aq. layer was extracted with EtOAc (3×200 mL), the combined organic fractions were dried over anhydrous $Na_2SO_4$, filtrated and the solvent was removed *in vacuo*. Purification by flash column chromatography (silica gel; *i*-hexane:EtOAc = 30:1) afforded **3b** as a yellow oil (2.31 g; 8.22 mmol; 95%).

Synthesis of 2-chloro-6-fluoro-4'-methoxybiphenyl (**3c**)

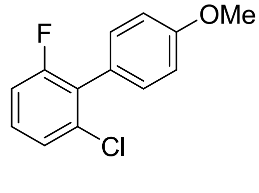

According to **TP1**, injection loop A and B were loaded with solutions of 1-chloro-3-fluorobenzene (**1b**; 0.43 M containing 0.5 equiv $ZnCl_2$·2LiCl) and $Cy_2NLi$ (0.65 M). After injection and in situ trapping metalation the combined streams were collected in a flask containing 4-iodoanisole (323 mg; 1.38 mmol; 0.8 equiv), $Pd(dba)_2$ (20 mg; 2 mol%) and tfp (16 mg; 4 mol%) dissolved in THF (2 mL). The reaction mixture was stirred overnight at 25 °C before it was quenched with water (20 mL). The aq. layer was extracted with EtOAc (3×70 mL), the combined organic fractions were dried over anhydrous $Na_2SO_4$, filtrated and the solvent was removed *in vacuo*. Purification by flash column chromatography (silica gel; *i*-hexane:EtOAc = 50:1) afforded **3c** as a colorless oil (320 mg; 1.35 mmol; 98%).

**$^1$H-NMR** (300 MHz, $CDCl_3$): $\delta$ / ppm = 7.33 – 7.28 (m, 3H), 7.27 – 7.20 (m, 1H), 7.10 – 7.04 (m, 1H), 7.03 – 6.99 (m, 2H), 3.87 (s, 3H).

**$^{13}$C-NMR** (75 MHz, $CDCl_3$): $\delta$ / ppm = 160.5 (d, $J$ = 247.5 Hz), 159.5, 134.6 (d, $J$ = 3.8 Hz), 131.4 (d, $J$ = 1.5 Hz), 128.7 (d, $J$ = 18.9 Hz), 128.7 (d, $J$ = 9.8 Hz), 125.4 (d, $J$ = 3.8 Hz), 124.7, 114.2 (d, $J$ = 23.4 Hz), 113.6, 55.2.

**IR** (Diamond-ATR, neat): $\tilde{\nu}$ / cm$^{-1}$ = 3003, 2936, 2837, 1892, 1602, 1579, 1565, 1518, 1445, 1410, 1293, 1244, 1177, 1109, 1074, 1036, 1020, 1002, 964, 936, 889, 828, 805, 780, 738, 717.

**MS** (EI, 70 eV): m/z (%) = 238 (32), 237 (15, M$^+$), 236 (100), 221 (12), 195 (12), 193 (34), 167 (12), 158 (10).

**HRMS** (EI): calcd. for [$C_{13}H_{10}ClFO$]: 236.0404; found: 236.0399 (M$^+$).

Synthesis of (2-chloro-6-fluorophenyl)(phenyl)sulfane (**3d**)

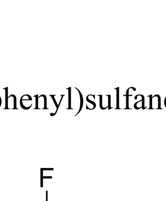


According to **TP1**, injection loop A and B were loaded with solutions of 1-chloro-3-fluorobenzene (**1b**; 0.44 M containing 0.5 equiv $MgCl_2$) and $Cy_2NLi$ (0.66 M). After injection and in situ trapping metalation the combined streams were collected in a flask containing S-

phenyl benzenethiosulfonate (353 mg; 1.41 mmol; 0.8 equiv) dissolved in THF (2 mL). The reaction mixture was stirred for further 30 min at 25 °C before it was quenched with water (30 mL). The aq. layer was extracted with EtOAc (3×60 mL), the combined organic fractions were dried over anhydrous $Na_2SO_4$, filtrated and the solvent was removed *in vacuo*. Purification by flash column chromatography (silica gel; *i*-hexane:EtOAc = 50:1) afforded **3d** as a colorless oil (253 mg; 1.06 mmol; 75%).

**$^1$H-NMR** (300 MHz, $CDCl_3$): $\delta$ / ppm = 7.36 – 7.15 (m, 7H), 7.12 – 7.05 (m, 1H).

**$^{13}$C-NMR** (75 MHz, $CDCl_3$): $\delta$ / ppm = 163.8 (d, $J$ = 250.5 Hz), 140.7 (d, $J$ = 0.8 Hz), 135.2 (d, $J$ = 1.5 Hz), 131.0 (d, $J$ = 9.8 Hz), 129.0, 128.4, 126.4, 126.0 (d, $J$ = 3.0 Hz), 120.9 (d, $J$ = 21.1 Hz), 114.8 (d, $J$ = 24.1 Hz).

**IR** (Diamond-ATR, neat): $\tilde{\nu}$ / cm$^{-1}$ = 3074, 2925, 2853, 1584, 1565, 1477, 1444, 1327, 1301, 1266, 1248, 1177, 1156, 1102, 1081, 1068, 1050, 1024, 999, 966, 888, 834, 778, 734, 711, 686.

**MS** (EI, 70 eV): m/z (%) = 240 (34), 239 (14, $M^+$), 238 (86), 204 (16), 203 (100), 202 (74), 126 (14), 109 (11), 101 (22), 77 (36), 65 (17), 57 (11), 51 (50), 50 (13).

**HRMS** (EI): calcd. for [$C_{12}H_8ClFS$]: 238.0019; found: 238.0036 ($M^+$).

Synthesis of ethyl 2',6'-difluorobiphenyl-4-carboxylate (**3e**)

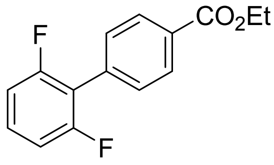


According to **TP1**, injection loop A and B were loaded with solutions of 1,3-difluorobenzene (**1c**; 0.44 M containing 0.5 equiv $ZnCl_2{\cdot}2LiCl$) and $Cy_2NLi$ (0.66 M). After injection and in situ trapping metalation the combined streams were collected in a flask containing ethyl 4-iodobenzoate (389 mg; 1.41 mmol; 0.8 equiv), $Pd(dba)_2$ (20 mg; 2 mol%) and tfp (16 mg; 4 mol%) dissolved in THF (2 mL). The reaction mixture was stirred for further 2 h at 25 °C before it was quenched with water (25 mL). The aq. layer was extracted with EtOAc (3×80 mL), the combined organic fractions were dried over anhydrous $Na_2SO_4$, filtrated and

the solvent was removed *in vacuo*. Purification by flash column chromatography (silica gel; *i*-hexane:EtOAc = 50:1) afforded **3e** as a colorless oil (350 mg; 1.33 mmol; 94%).

**$^{1}$H-NMR** (300 MHz, $CDCl_3$): $\delta$ / ppm = 8.13 (d, $J$ = 8.1 Hz, 2H), 7.55 (d, $J$ = 8.1 Hz, 2H), 7.36 – 7.26 (m, 1H), 7.04 – 6.95 (m, 2H), 4.41 (q, $J$ = 7.1 Hz, 2H), 1.41 (t, $J$ = 7.1 Hz, 3H).

**$^{13}$C-NMR** (75 MHz, $CDCl_3$): $\delta$ / ppm = 166.2, 159.9 (dd, $J$ = 249.7 Hz, $J$ = 6.8 Hz), 133.8, 130.8 (t, $J$ = 2.3 Hz), 130.2, 129.6 (t, $J$ = 10.6 Hz), 129.4, 117.6 (t, $J$ = 18.9 Hz), 111.7 (dd, $J$ = 18.1 Hz, $J$ = 8.3 Hz), 61.0, 14.3.

**IR** (Diamond-ATR, neat): $\tilde{\nu}$ / cm$^{-1}$ = 3068, 2983, 2935, 2906, 1935, 1714, 1625, 1612, 1588, 1578, 1566, 1519, 1466, 1404, 1368, 1314, 1266, 1230, 1183, 1101, 1070, 1028, 1009, 997, 858, 787, 771, 723, 702.

**MS** (EI, 70 eV): m/z (%) = 262 (23, $M^+$), 234 (35), 218 (14), 217 (73), 189 (33), 188 (29), 123 (14), 111 (23), 109 (18), 97 (40), 95 (23), 85 (40), 83 (36), 82 (14), 81 (28), 71 (57), 70 (14), 69 (48), 67 (24), 57 (100), 56 (20), 55 (68), 44 (30), 43 (78), 43 (14), 41 (46).

**HRMS** (EI): calcd. for [$C_{15}H_{12}F_2O_2$]: 262.0805; found: 262.0803 ($M^+$).

Synthesis of ethyl 2,6-difluorobenzoate (**3f**)

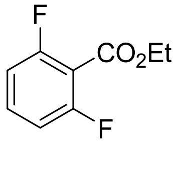


According to **TP1**, injection loop A and B were loaded with solutions of 1,3-difluorobenzene (**1c**; 0.45 M containing 0.5 equiv $MgCl_2$) and $Cy_2NLi$ (0.67 M). After injection and in situ trapping metalation the combined streams were collected in a flask containing ethyl cyanoformate (143 mg; 1.44 mmol; 0.8 equiv) dissolved in THF (2 mL). The reaction mixture was stirred for further 30 min at 25 °C before it was quenched with water (30 mL). The aq. layer was extracted with EtOAc (3×70 mL), the combined organic fractions were dried over anhydrous $Na_2SO_4$, filtrated and the solvent was removed *in vacuo*. Purification by flash column chromatography (silica gel; *i*-hexane:EtOAc = 30:1) afforded **3f** as a yellow oil (178 mg; 0.96 mmol; 67%).

**$^1$H-NMR** (400 MHz, $CDCl_3$): $\delta$ / ppm = 7.41 – 7.33 (m, 1H), 6.94 – 6.89 (m, 2H), 4.40 (q, $J$ = 7.1 Hz, 2H), 1.37 (t, $J$ = 7.1 Hz, 3H).

**$^{13}$C-NMR** (101 MHz, $CDCl_3$): $\delta$ / ppm = 161.5 (t, $J$ = 1.0 Hz), 160.6 (dd, $J$ = 256.4 Hz, $J$ = 6.0 Hz), 132.4 (t, $J$ = 11.1 Hz), 111.9 (dd, $J$ = 21.1 Hz, $J$ = 5.0 Hz), 111.5 (t, $J$ = 18.1 Hz), 62.0, 14.1.

**IR** (Diamond-ATR, neat): $\tilde{\nu}$ / cm$^{-1}$ = 2986, 2937, 1730, 1625, 1595, 1469, 1392, 1368, 1287, 1262, 1235, 1174, 1107, 1058, 1006, 856, 796, 767, 701, 583, 564.

**MS** (EI, 70 eV): m/z (%) = 186 (33, $M^+$), 159 (11), 158 (100), 142 (38), 114 (12), 113 (72), 63 (43).

**HRMS** (EI): calcd. for [$C_9H_8F_2O_2$]: 186.0492; found: 186.0490 ($M^+$).

Synthesis of triethyl 5-bromobiphenyl-2,4,4'-tricarboxylate (**3g**)

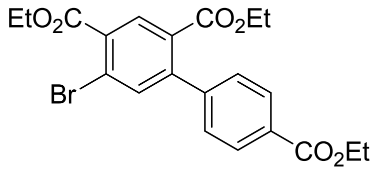


According to **TP1**, injection loop A and B were loaded with solutions of diethyl 4-bromoisophthalate (**1d**; 0.45 M containing 0.5 equiv $ZnCl_2 \cdot 2LiCl$) and $Cy_2NLi$ (0.68 M). After injection and in situ trapping metalation the combined streams were collected in a flask containing ethyl 4-iodobenzoate (398 mg; 1.44 mmol; 0.8 equiv), $Pd(dba)_2$ (21 mg; 2 mol%) and tfp (17 mg; 4 mol%) dissolved in THF (2 mL). The reaction mixture was stirred for further 2 h at 25 °C before it was quenched with water (20 mL). The aq. layer was extracted with EtOAc (3×70 mL), the combined organic fractions were dried over anhydrous $Na_2SO_4$, filtrated and the solvent was removed *in vacuo*. Purification by flash column chromatography (silica gel; *i*-hexane:EtOAc = 15:1) afforded **3g** as an orange solid (463 mg; 1.03 mmol; 72%).

**m.p.** (°C): 89.2 – 91.6.

**$^1$H-NMR** (300 MHz, $CDCl_3$): $\delta$ / ppm = 8.29 (s, 1H), 8.06 (d, $J$ = 8.1 Hz, 2H), 7.65 (s, 1H), 7.34 (d, $J$ = 8.1 Hz, 2H), 4.45 – 4.33 (m, 4H), 4.08 (q, $J$ = 7.1 Hz, 2H), 1.43 – 1.37 (m, 6H), 1.00 (t, $J$ = 7.1 Hz, 3H).

**$^{13}$C-NMR** (75 MHz, $CDCl_3$): $\delta$ / ppm = 166.5, 166.1, 165.0, 145.4, 143.7, 136.3, 133.0, 131.7, 130.1, 129.9, 129.4, 128.2, 125.0, 62.0, 61.5, 61.1, 14.3, 14.2, 13.7.

**IR** (Diamond-ATR, neat): $\tilde{\nu}$ / cm$^{-1}$ = 3076, 2980, 2938, 2907, 2872, 1709, 1594, 1535, 1466, 1445, 1405, 1392, 1365, 1353, 1309, 1270, 1228, 1175, 1098, 1012, 931, 888, 864, 853, 839, 773, 720, 705, 696, 666.

**MS** (EI, 70 eV): m/z (%) = 451 (17), 450 (74), 449 (21, $M^+$), 448 (73), 406 (21), 405 (100), 404 (23), 403 (99), 377 (28), 376 (12), 375 (28), 349 (12), 347 (12), 331 (18), 166 (10), 165 (10), 150 (16).

**HRMS** (EI): calcd. for [$C_{21}H_{21}BrO_6$]: 448.0522; found: 448.0508 ($M^+$).

Synthesis of 4'-methoxy-5-(trifluoromethyl)biphenyl-2-carbonitrile (**3h**)

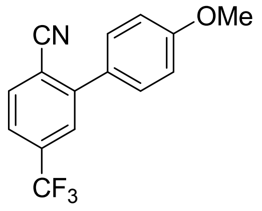


According to **TP1**, injection loop A and B were loaded with solutions of 4-(trifluoromethyl)benzonitrile (**1e**; 0.44 M containing 0.5 equiv $ZnCl_2$·2LiCl) and $Cy_2NLi$ (0.66 M). After injection and in situ trapping metalation the combined streams were collected in a flask containing 4-iodoanisole (330 mg; 1.41 mmol; 0.8 equiv), $Pd(dba)_2$ (20 mg; 2 mol%) and tfp (16 mg; 4 mol%) dissolved in THF (2 mL). The reaction mixture was stirred overnight at 25 °C before it was quenched with water (20 mL). The aq. layer was extracted with EtOAc (3×60 mL), the combined organic fractions were dried over anhydrous $Na_2SO_4$, filtrated and the solvent was removed *in vacuo*. Purification by flash column chromatography (silica gel; *i*-hexane:EtOAc = 40:1) afforded **3h** as a colorless solid (274 mg; 0.99 mmol; 70%).

**m.p.** (°C): 128.0 – 129.3.

**$^1$H-NMR** (400 MHz, $CDCl_3$): $\delta$ / ppm = 7.85 (d, $J$ = 8.1 Hz, 1H), 7.73 (s, 1H), 7.64 (d, $J$ = 8.1 Hz, 1H), 7.53 – 7.49 (m, 2H), 7.05 – 7.01 (m, 2H), 3.86 (s, 3H).

**$^{13}$C-NMR** (101 MHz, $CDCl_3$): $\delta$ / ppm = 160.7, 146.1, 134.5 (q, $J$ = 33.2 Hz), 134.3, 130.1, 129.1, 126.7 (q, $J$ = 4.0 Hz), 123.7 (q, $J$ = 4.0 Hz), 123.1 (q, $J$ = 273.6 Hz), 117.7, 114.5, 114.5, 55.4.

**IR** (Diamond-ATR, neat): $\tilde{\nu}$ / cm$^{-1}$ = 3058, 3002, 2947, 2847, 2227, 1900, 1719, 1607, 1576, 1519, 1491, 1465, 1454, 1447, 1423, 1407, 1338, 1313, 1293, 1265, 1256, 1249, 1183, 1161, 1132, 1108, 1074, 1037, 1019, 972, 944, 909, 853, 932, 818, 793, 754, 737, 726, 713, 697, 663.

**MS** (EI, 70 eV): m/z (%) = 278 (19), 277 (100, $M^+$), 234 (38).

**HRMS** (EI): calcd. for [$C_{15}H_{10}F_3NO$]: 277.0714; found: 277.0712 ($M^+$).

Synthesis of 4-chloro-2-fluoro-3'-methoxybiphenyl-3-carbonitrile (**3i**)

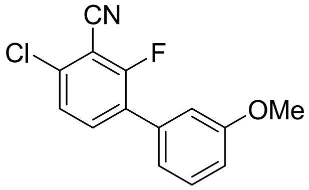


According to **TP1**, injection loop A and B were loaded with solutions of 2-chloro-6-fluorobenzonitrile (**1f**; 0.45 M containing 0.5 equiv $ZnCl_2 \cdot 2LiCl$) and $Cy_2NLi$ (0.67 M). After injection and in situ trapping metalation the combined streams were collected in a flask containing 3-iodoanisole (337 mg; 1.44 mmol; 0.8 equiv), $Pd(dba)_2$ (21 mg; 2 mol%) and tfp (17 mg; 4 mol%) dissolved in THF (2 mL). The reaction mixture was stirred for further 2 h at 25 °C before it was quenched with water (25 mL). The aq. layer was extracted with EtOAc (3×50 mL), the combined organic fractions were dried over anhydrous $Na_2SO_4$, filtrated and the solvent was removed *in vacuo*. Purification by flash column chromatography (silica gel; *i*-hexane:EtOAc = 15:1) afforded **3i** as a yellow solid (365 mg; 1.39 mmol; 97%).

**m.p.** (°C): 103.8 – 105.5.

**$^1$H-NMR** (400 MHz, $CDCl_3$): $\delta$ / ppm = 7.62 – 7.58 (m, 1H), 7.40 – 7.35 (m, 2H), 7.06 – 7.03 (m, 1H), 7.00 – 6.95 (m, 2H), 3.84 (s, 3H).

**$^{13}$C-NMR** (101 MHz, $CDCl_3$): $\delta$ / ppm = 160.7 (d, $J$ = 263.5 Hz), 159.9, 136.5 (d, $J$ = 2.0 Hz), 135.4 (d, $J$ = 5.0 Hz), 134.0 (d, $J$ = 1.0 Hz), 130.0, 128.7 (d, $J$ = 12.1 Hz), 125.8 (d, $J$ = 5.0 Hz), 121.1 (d, $J$ = 3.0 Hz), 114.6 (d, $J$ = 3.0 Hz), 114.4, 111.4, 104.0 (d, $J$ = 19.1 Hz), 55.4.

**IR** (Diamond-ATR, neat): $\tilde{\nu}$ / cm$^{-1}$ = 3107, 3049, 3007, 2945, 2893, 2837, 2239, 1652, 1608, 1579, 1561, 1492, 1463, 1406, 1339, 1313, 1289, 1273, 1228, 1216, 1175, 1150, 1114, 1095, 1076, 994, 930, 906, 885, 872, 826, 794, 777, 744, 718, 694, 684.

**MS** (EI, 70 eV): m/z (%) = 263 (33), 262 (15, $M^+$), 261 (100), 231 (25), 218 (24), 195 (10), 182 (11).

**HRMS** (EI): calcd. for [$C_{14}H_9ClFNO$]: 261.0357; found: 261.0352 ($M^+$).

Synthesis of 4-chloro-2-fluorobiphenyl-3,4'-dicarbonitrile (**3j**)

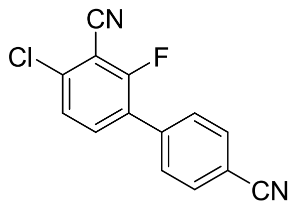


According to **TP1**, injection loop A and B were loaded with solutions of 2-chloro-6-fluorobenzonitrile (**1f**; 0.45 M containing 0.5 equiv $ZnCl_2{\cdot}2LiCl$) and $Cy_2NLi$ (0.67 M). After injection and in situ trapping metalation the combined streams were collected in a flask containing 4-iodobenzonitrile (330 mg; 1.44 mmol; 0.8 equiv), $Pd(dba)_2$ (21 mg; 2 mol%) and tfp (17 mg; 4 mol%) dissolved in THF (2 mL). The reaction mixture was stirred for further 2 h at 25 °C before it was quenched with water (25 mL). The aq. layer was extracted with EtOAc (3×60 mL), the combined organic fractions were dried over anhydrous $Na_2SO_4$, filtrated and the solvent was removed *in vacuo*. Purification by flash column chromatography (silica gel; *i*-hexane:EtOAc = 5:1) afforded **3j** as a colorless solid (269 mg; 1.05 mmol; 73%).

**m.p.** (°C): 223.0 – 224.8.

$^1$**H-NMR** (400 MHz, $CDCl_3$): $\delta$ / ppm = 7.78 – 7.76 (m, 2H), 7.63 – 7.59 (m, 3H), 7.45 – 7.43 (m, 1H).

$^{13}$**C-NMR** (101 MHz, $CDCl_3$): $\delta$ / ppm = 160.5 (d, $J$ = 264.5 Hz), 138.0 (d, $J$ = 2.0 Hz), 137.2 (d, $J$ = 4.0 Hz), 135.0 (d, $J$ = 5.0 Hz), 132.7, 129.5 (d, $J$ = 3.0 Hz), 126.9 (d, $J$ = 12.1 Hz), 126.3 (d, $J$ = 4.0 Hz), 118.2, 113.0, 110.9, 104.6.

**IR** (Diamond-ATR, neat): $\tilde{\nu}$ / cm$^{-1}$ = 3083, 2925, 2235, 1940, 1660, 1609, 1570, 1459, 1430, 1395, 1307, 1282, 1266, 1229, 1208, 1183, 1157, 1115, 1079, 1016, 969, 914, 885, 859, 854, 834, 823, 794, 770, 751, 693, 667.

**MS** (EI, 70 eV): m/z (%) = 258 (32), 257 (15, $M^+$), 256 (100), 220 (11), 194 (10).

**HRMS** (EI): calcd. for [$C_{14}H_6ClFN_2$]: 256.0204; found: 256.0191 ($M^+$).

Synthesis of ethyl 4-(2-fluoropyridin-3-yl)benzoate (**5a**)

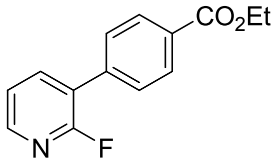


According to **TP1**, injection loop A and B were loaded with solutions of 2-fluoropyridine (**4a**; 0.44 M containing 0.5 equiv $ZnCl_2 \cdot 2LiCl$) and $Cy_2NLi$ (0.66 M). After injection and in situ trapping metalation the combined streams were collected in a flask containing ethyl 4-iodobenzoate (389 mg; 1.41 mmol; 0.8 equiv), $Pd(dba)_2$ (20 mg; 2 mol%) and tfp (16 mg; 4 mol%) dissolved in THF (2 mL). The reaction mixture was stirred for further 2 h at 25 °C before it was quenched with water (30 mL). The aq. layer was extracted with EtOAc (3×50 mL), the combined organic fractions were dried over anhydrous $Na_2SO_4$, filtrated and the solvent was removed *in vacuo*. Purification by flash column chromatography (silica gel; *i*-hexane:EtOAc = 5:1 + 0.5 vol% $NEt_3$) afforded **5a** as a yellow oil (327 mg; 1.33 mmol; 94%).

$^1$**H-NMR** (300 MHz, $CDCl_3$): $\delta$ / ppm = 8.22 – 8.20 (m, 1H), 8.13 – 8.09 (m, 2H), 7.91 – 7.84 (m, 1H), 7.64 – 7.59 (m, 2H), 7.30 – 7.26 (m, 1H), 4.38 (q, $J$ = 7.1 Hz, 2H), 1.39 (t, $J$ = 7.1 Hz, 3H).

**$^{13}$C-NMR** (75 MHz, $CDCl_3$): $\delta$ / ppm = 166.1, 160.3 (d, $J$ = 240.7 Hz), 147.1 (d, $J$ = 15.1 Hz), 140.7 (d, $J$ = 4.5 Hz), 138.2 (d, $J$ = 5.3 Hz), 130.4, 129.9, 128.7 (d, $J$ = 3.0 Hz), 122.9 (d, $J$ = 28.7 Hz), 121.9 (d, $J$ = 4.5 Hz), 61.1, 14.3.

**IR** (Diamond-ATR, neat): $\tilde{\nu}$ / $cm^{-1}$ = 3061, 2982, 2933, 2907, 2874, 1712, 1611, 1603, 1577, 1562, 1514, 1476, 1438, 1397, 1367, 1314, 1269, 1246, 1204, 1183, 1101, 1025, 1003, 855, 845, 805, 773, 757, 731, 702, 645, 634, 616, 608, 591, 570.

**MS** (EI, 70 eV): m/z (%) = 245 (35, $M^+$), 217 (31), 201 (17), 200 (100), 172 (30), 145 (16), 125 (10).

**HRMS** (EI): calcd. for [$C_{14}H_{12}FNO_2$]: 245.0802; found: 245.0840 ($M^+$).

Synthesis of 2-fluoro-3-(methylthio)pyridine (**5b**)

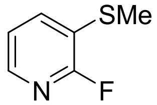


According to **TP1**, injection loop A and B were loaded with solutions of 2-fluoropyridine (**4a**; 0.44 M containing 0.5 equiv $MgCl_2$) and $Cy_2NLi$ (0.66 M). After injection and in situ trapping metalation the combined streams were collected in a flask containing S-methyl methanethiosulfonate (178 mg; 1.41 mmol; 0.8 equiv) dissolved in THF (2 mL). The reaction mixture was stirred for further 30 min at 25 °C before it was quenched with water (25 mL). The aq. layer was extracted with EtOAc (3×60 mL), the combined organic fractions were dried over anhydrous $Na_2SO_4$, filtrated and the solvent was removed *in vacuo*. Purification by flash column chromatography (silica gel; *i*-hexane:EtOAc = 5:1 + 0.5 vol% $NEt_3$) afforded **5b** as an orange oil (152 mg; 1.06 mmol; 75%).

**$^1$H-NMR** (300 MHz, $CDCl_3$): $\delta$ / ppm = 7.96 – 7.93 (m, 1H), 7.62 – 7.55 (m, 1H), 7.14 – 7.09 (m, 1H), 2.45 (s, 3H).

**$^{13}$C-NMR** (75 MHz, $CDCl_3$): $\delta$ / ppm = 160.1 (d, $J$ = 236.2 Hz), 143.2 (d, $J$ = 14.3 Hz), 137.5 (d, $J$ = 3.8 Hz), 122.1 (d, $J$ = 33.2 Hz), 121.7 (d, $J$ = 4.5 Hz), 14.8 (d, $J$ = 1.5 Hz).

**IR** (Diamond-ATR, neat): $\tilde{\nu}$ / cm$^{-1}$ = 3065, 2959, 2925, 2855, 1585, 1559, 1446, 1408, 1322, 1303, 1289, 1233, 1189, 1141, 1082, 1047, 971, 959, 921, 901, 840, 789, 742, 708, 678, 620, 554.

**MS** (EI, 70 eV): m/z (%) = 143 (100, M$^+$), 110 (12).

**HRMS** (EI): calcd. for [$C_6H_6FNS$]: 143.0205; found: 143.0193 (M$^+$).

Synthesis of ethyl 4-(2,6-dibromopyridin-4-yl)benzoate (**5c**)

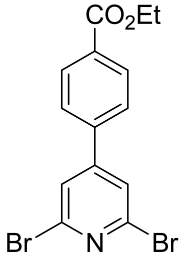


According to **TP1**, injection loop A and B were loaded with solutions of 2,6-dibromopyridine (**4b**; 0.45 M containing 0.5 equiv $ZnCl_2$·2LiCl) and $Cy_2NLi$ (0.67 M). After injection and in situ trapping metalation the combined streams were collected in a flask containing ethyl 4-iodobenzoate (398 mg; 1.44 mmol; 0.8 equiv), $Pd(dba)_2$ (21 mg; 2 mol%) and tfp (17 mg; 4 mol%) dissolved in THF (2 mL). The reaction mixture was stirred for further 2 h at 25 °C before it was quenched with water (20 mL). The aq. layer was extracted with EtOAc (3×60 mL), the combined organic fractions were dried over anhydrous $Na_2SO_4$, filtrated and the solvent was removed *in vacuo*. Purification by flash column chromatography (silica gel; *i*-hexane:EtOAc = 30:1 + 0.5 vol% $NEt_3$) afforded **5c** as a colorless solid (371 mg; 0.96 mmol; 67%).

**m.p.** (°C): 114.9 – 116.5.

**$^1$H-NMR** (400 MHz, $CDCl_3$): $\delta$ / ppm = 8.15 – 8.12 (m, 2H), 7.65 (s, 2H), 7.63 – 7.60 (m, 2H), 4.40 (q, $J$ = 7.1 Hz, 2H), 1.40 (t, $J$ = 7.1 Hz, 3H).

**$^{13}$C-NMR** (101 MHz, $CDCl_3$): $\delta$ / ppm = 165.8, 152.1, 141.5, 139.5, 132.0, 130.5, 127.2, 125.1, 61.4, 14.3.

**IR** (Diamond-ATR, neat): $\tilde{\nu}$ / cm$^{-1}$ = 3098, 3066, 2983, 2931, 2910, 2866, 1714, 1664, 1613, 1575, 1515, 1482, 1467, 1439, 1407, 1358, 1314, 1276, 1221, 1183, 1168, 1154, 1119, 1106, 1092, 1063, 1025, 1018, 979, 883, 850, 817, 800, 774, 758, 718, 701, 670.

**MS** (EI, 70 eV): m/z (%) = 387 (22), 385 (44, M$^+$), 383 (23), 359 (30), 357 (59), 355 (29), 342 (54), 341 (17), 340 (100), 338 (50), 233 (30), 231 (30), 153 (29), 152 (48), 151 (23), 126 (18), 125 (15), 76 (10).

**HRMS** (EI): calcd. for [$C_{14}H_{11}Br_2NO_2$]: 382.9157; found: 382.9150 (M$^+$).

Synthesis of ethyl 2-chloro-4-(cyclohex-2-enyl)nicotinate (**5d**)

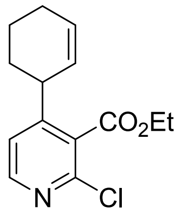


According to **TP1**, injection loop A and B were loaded with solutions of ethyl 2-chloronicotinate (**4c**; 0.43 M containing 0.5 equiv $ZnCl_2$·2LiCl) and $Cy_2NLi$ (0.65 M). After injection and in situ trapping metalation the combined streams were collected in a flask containing 3-bromocyclohexene (222 mg; 1.38 mmol; 0.8 equiv) and CuCN·2LiCl (0.17 mL; 10 mol%) dissolved in THF (2 mL) and cooled to 0 °C. The reaction mixture was stirred for further 1.5 h at 0 °C before it was quenched with sat. aq. $NH_4Cl/NH_3$ (10 vol%; 20 mL). The aq. layer was extracted with EtOAc (3×60 mL), the combined organic fractions were dried over anhydrous $Na_2SO_4$, filtrated and the solvent was removed *in vacuo*. Purification by flash column chromatography (silica gel; *i*-hexane:EtOAc = 20:1 + 0.5 vol% $NEt_3$) afforded **5d** as a colorless oil (321 mg; 1.21 mmol; 88%).

**$^1$H-NMR** (300 MHz, $CDCl_3$): $\delta$ / ppm = 8.29 (d, $J$ = 5.2 Hz, 1H), 7.15 (d, $J$ = 5.2 Hz, 1H), 5.99 – 5.92 (m, 1H), 5.56 – 5.52 (m, 1H), 4.50 – 4.33 (m, 2H), 3.45 – 3.38 (m, 1H), 2.08 – 1.98 (m, 3H), 1.75 – 1.43 (m, 3H), 1.40 – 1.35 (m, 3H).

**$^{13}$C-NMR** (75 MHz, $CDCl_3$): $\delta$ / ppm = 165.9, 155.6, 149.7, 147.4, 130.5, 129.8, 127.1, 121.8, 62.1, 39.2, 31.2, 24.6, 20.8, 14.0.

**IR** (Diamond-ATR, neat): $\tilde{\nu}$ / cm$^{-1}$ = 3023, 2982, 2932, 2861, 2838, 1730, 1650, 1579, 1546, 1447, 1393, 1379, 1363, 1309, 1270, 1226, 1215, 1180, 1119, 1095, 1061, 1044, 1012, 938, 910, 890, 852, 841, 803, 736, 724, 694.

**MS** (EI, 70 eV): m/z (%) = 267 (15), 265 (43), 236 (19), 222 (16), 221 (39), 220 (58), 219 (100), 218 (44), 203 (14), 202 (24), 201 (41), 200 (40), 190 (13), 184 (23), 183 (25), 182 (83), 166 (36), 156 (16), 155 (14), 154 (42), 128 (28), 127 (24), 116 (10), 115 (12), 77 (17), 41 (11).

**HRMS** (EI): calcd. for [$C_{14}H_{16}ClNO_2$]: 265.0870; found: 265.0862 ($M^+$).

Synthesis of 2,3-dichloro-5-(3-methoxyphenyl)pyrazine (**5e**)

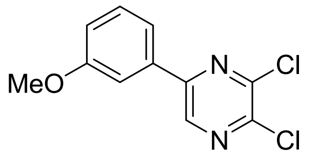


According to **TP1**, injection loop A and B were loaded with solutions of 2,3-dichloropyrazine (**4d**; 0.44 M containing 0.5 equiv $ZnCl_2$·2LiCl) and $Cy_2NLi$ (0.66 M). After injection and in situ trapping metalation the combined streams were collected in a flask containing 3-iodoanisole (330 mg; 1.44 mmol; 0.8 equiv), $Pd(dba)_2$ (20 mg; 2 mol%) and tfp (16 mg; 4 mol%) dissolved in THF (2 mL). The reaction mixture was stirred overnight at 25 °C before it was quenched with water (25 mL). The aq. layer was extracted with EtOAc (3×70 mL), the combined organic fractions were dried over anhydrous $Na_2SO_4$, filtrated and the solvent was removed *in vacuo*. Purification by flash column chromatography (silica gel; *i*-hexane:EtOAc = 30:1 + 0.5 vol% $NEt_3$) afforded **5e** as a yellow solid (275 mg; 1.08 mmol; 77%).

**m.p.** (°C): 111.0 – 112.7.

**$^1$H-NMR** (400 MHz, $CDCl_3$): $\delta$ / ppm = 8.67 (s, 1H), 7.53 – 7.51 (m, 2H), 7.41 – 7.37 (m, 1H), 7.03 – 7.00 (m, 1H), 3.87 (s, 3H).

**$^{13}$C-NMR** (101 MHz, $CDCl_3$): $\delta$ / ppm = 160.4, 150.7, 146.8, 145.4, 138.5, 135.1, 130.3, 119.3, 116.6, 112.4, 55.5.

**IR** (Diamond-ATR, neat): $\tilde{\nu}$ / cm$^{-1}$ = 3057, 2938, 2840, 1715, 1607, 1580, 1545, 1506, 1491, 1465, 1451, 1434, 1415, 1351, 1323, 1300, 1273, 1220, 1207, 1196, 1175, 1161, 1065, 1035, 925, 887, 859, 786, 753, 694, 677, 659.

**MS** (EI, 70 eV): m/z (%) = 257 (10), 256 (63), 254 (100), 253 (21), 226 (11), 225 (11), 224 (15), 211 (11); 162 (10), 133 (13), 114 (10), 103 (19), 90 (10), 89 (19), 63 (23).

**HRMS** (EI): calcd. for [$C_{11}H_8Cl_2N_2O$]: 254.0014; found: 254.0014 ($M^+$).

Synthesis of (4-chlorophenyl)(1-methyl-1H-pyrazol-5-yl)methanol (**5f**)

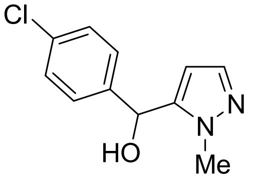


According to **TP1**, injection loop A and B were loaded with solutions of 1-methylpyrazole (**4e**; 0.43 M containing 0.5 equiv $LaCl_3 \cdot 2LiCl$) and $Cy_2NLi$ (0.65 M). After injection and in situ trapping metalation the combined streams were collected in a flask containing 4-chlorobenzaldehyde (194 mg; 1.38 mmol; 0.8 equiv) dissolved in THF (2 mL). The reaction mixture was stirred for further 1 h at 25 °C before it was quenched with water (20 mL). The aq. layer was extracted with EtOAc (3×60 mL), the combined organic fractions were dried over anhydrous $Na_2SO_4$, filtrated and the solvent was removed *in vacuo*. Purification by flash column chromatography (silica gel; *i*-hexane:EtOAc = 10:1 + 0.5 vol% $NEt_3$ → EtOAc + 0.5 vol% $NEt_3$) afforded **5f** as an orange oil (192 mg; 0.86 mmol; 62%).

**$^1$H-NMR** (400 MHz, $CDCl_3$): $\delta$ / ppm = 7.32 – 7.26 (m, 5H), 5.96 (s, 1H), 5.83 (s, 1H), 3.70 (s, 3H), 3.32 (br, 1H).

**$^{13}$C-NMR** (101 MHz, $CDCl_3$): $\delta$ / ppm = 143.8, 139.5, 137.8, 133.9, 128.7, 127.8, 105.9, 67.6, 37.1.

**IR** (Diamond-ATR, neat): $\tilde{\nu}$ / cm$^{-1}$ = 3215, 2944, 2858, 1596, 1578, 1488, 1397, 1326, 1282, 1199, 1087, 1052, 1013, 936, 853, 841, 799, 777, 764, 725, 709, 686, 672.

**MS** (EI, 70 eV): m/z (%) = 224 (32), 223 (18, $M^+$), 222 (100), 221 (12), 207 (20), 205 (50), 141 (17), 139 (46), 112 (17), 111 (34), 111 (20), 110 (19), 109 (97), 105 (18), 95 (10), 83 (64), 82 (11), 77 (35), 75 (14), 56 (11), 42 (14).

**HRMS** (EI): calcd. for [$C_{11}H_{11}ClN_2O$]: 222.0560; found: 222.0553 ($M^+$).

Synthesis of ethyl 5-bromo-3-(cyclohex-2-enyl)furan-2-carboxylate (**5g**)

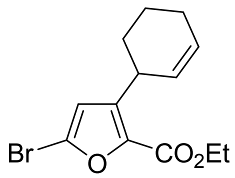


According to **TP1**, injection loop A and B were loaded with solutions of ethyl 5-bromofuran-2-carboxylate (**4f**; 0.45 M containing 0.5 equiv $ZnCl_2$·2LiCl) and $Cy_2NLi$ (0.68 M). After injection and in situ trapping metalation the combined streams were collected in a flask containing 3-bromocyclohexene (232 mg; 1.44 mmol; 0.8 equiv) and CuCN·2LiCl (0.18 mL; 10 mol%) dissolved in THF (2 mL) and cooled to 0 °C. The reaction mixture was stirred for further 1 h at 0 °C before it was quenched with sat. aq. $NH_4Cl/NH_3$ (10 vol%; 20 mL). The aq. layer was extracted with EtOAc (3×60 mL), the combined organic fractions were dried over anhydrous $Na_2SO_4$, filtrated and the solvent was removed *in vacuo*. Purification by flash column chromatography (silica gel; *i*-hexane:EtOAc = 100:1) afforded **5g** as a colorless oil (327 mg; 1.09 mmol; 76%).

**$^1$H-NMR** (400 MHz, $CDCl_3$): $\delta$ / ppm = 6.33 (s, 1H), 5.83 – 5.78 (m, 1H), 5.54 – 5.50 (m, 1H), 4.34 – 4.28 (m, 2H), 4.05 – 4.00 (m, 1H), 2.04 – 1.93 (m, 3H), 1.73 – 1.42 (m, 3H), 1.35 – 1.31 (m, 3H).

**$^{13}$C-NMR** (101 MHz, $CDCl_3$): $\delta$ / ppm = 158.3, 142.3, 141.2, 128.9, 128.3, 126.2, 114.7, 60.8, 32.3, 30.0, 24.8, 21.1, 14.3.

**IR** (Diamond-ATR, neat): $\tilde{\nu}$ / $cm^{-1}$ = 2931, 1705, 1591, 1476, 1446, 1398, 1383, 1308, 1258, 1143, 1094, 1068, 1037, 1015, 979, 933, 896, 881, 865, 814, 762, 724, 678.

**MS** (EI, 70 eV): m/z (%) = 300 (49), 298 (51), 271 (45), 269 (43), 254 (29), 253 (91), 252 (32), 251 (100), 225 (32), 223 (28), 145 (21), 144 (23), 117 (56), 116 (29), 115 (60), 91 (53), 89 (21), 81 (28), 79 (34), 78 (22), 77 (43), 67 (19), 65 (24), 63 (26), 51 (33), 41 (41).

**HRMS** (EI): calcd. for [$C_{13}H_{15}BrO_3$]: 298.0205; found: 298.0198 ($M^+$).

Synthesis of 2-bromo-5-(4-(trifluoromethyl)phenyl)thiophene (**5h**)

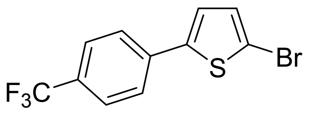


According to **TP1**, injection loop A and B were loaded with solutions of 2-bromothiophene (**4g**; 0.44 M containing 0.5 equiv $ZnCl_2 \cdot 2LiCl$) and $Cy_2NLi$ (0.66 M). After injection and in situ trapping metalation the combined streams were collected in a flask containing 4-iodobenzotrifluoride (384 mg; 1.41 mmol; 0.8 equiv), $Pd(dba)_2$ (20 mg; 2 mol%) and tfp (16 mg; 4 mol%) dissolved in THF (2 mL). The reaction mixture was stirred for further 2 h at 25 °C before it was quenched with water (20 mL). The aq. layer was extracted with EtOAc (3×60 mL), the combined organic fractions were dried over anhydrous $Na_2SO_4$, filtrated and the solvent was removed *in vacuo*. Purification by flash column chromatography (silica gel; *i*-hexane) afforded **5h** as a colorless solid (396 mg; 1.29 mmol; 91%).

**m.p.** (°C): 96.0 – 97.8.

**$^1$H-NMR** (400 MHz, $CDCl_3$): $\delta$ / ppm = 7.62 – 7.57 (m, 4H), 7.12 (d, $J$ = 3.9 Hz, 1H), 7.05 (d, $J$ = 3.9 Hz, 1H).

**$^{13}$C-NMR** (101 MHz, $CDCl_3$): $\delta$ / ppm = 144.0, 136.9, 131.2, 129.7 (q, $J$ = 33.2 Hz), 126.1 (q, $J$ = 4.0 Hz), 125.7, 124.7, 124.0 (q, $J$ = 271.6 Hz), 133.1.

**IR** (Diamond-ATR, neat): $\tilde{\nu}$ / cm$^{-1}$ = 2924, 1914, 1759, 1612, 1571, 1506, 1429, 1411, 1324, 1283, 1245, 1209, 1191, 1161, 1127, 1109, 1071, 1059, 1017, 1008, 979, 944, 880, 834, 799, 775, 735, 696.

**MS** (EI, 70 eV): m/z (%) = 309 (12), 308 (96), 307 (10, $M^+$), 306 (100), 183 (67), 133 (10), 97 (10), 83 (12), 71 (17), 69 (16), 57 (25), 55 (16), 43 (18), 43 (27), 41 (18).

**HRMS** (EI): calcd. for $[C_{11}H_6BrF_3S]$: 305.9326; found: 305.9323 ($M^+$).

Synthesis of 2-bromo-5-(3-nitrophenyl)thiophene (**5i**)

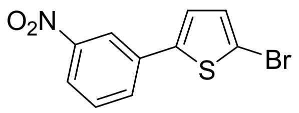

According to **TP1**, injection loop A and B were loaded with solutions of 2-bromothiophene (**4g**; 0.44 M containing 0.5 equiv $ZnCl_2 \cdot 2LiCl$) and $Cy_2NLi$ (0.66 M). After injection and in situ trapping metalation the combined streams were collected in a flask containing 1-iodo-3-nitrobenzene (351 mg; 1.41 mmol; 0.8 equiv), $Pd(dba)_2$ (20 mg; 2 mol%) and tfp (16 mg; 4 mol%) dissolved in THF (2 mL). The reaction mixture was stirred for further 2 h at 25 °C before it was quenched with water (20 mL). The aq. layer was extracted with EtOAc (3×60 mL), the combined organic fractions were dried over anhydrous $Na_2SO_4$, filtrated and the solvent was removed *in vacuo*. Purification by flash column chromatography (silica gel; *i*-hexane:EtOAc = 80:1) afforded **5i** as a yellow solid (354 mg; 1.25 mmol; 89%).

**m.p.** (°C): 114.5 – 116.7.

**$^1$H-NMR** (300 MHz, $CDCl_3$): $\delta$ / ppm = 8.32 (t, $J$ = 2.0 Hz, 1H), 8.10 (ddd, $J$ = 8.1 Hz, $J$ = 2.1 Hz, $J$ = 1.0 Hz, 1H), 7.77 (ddd, $J$ = 7.9 Hz, $J$ = 1.9 Hz, $J$ = 1.0 Hz, 1H), 7.52 (t, $J$ = 8.0 Hz, 1H), 7.15 (d, $J$ = 3.9 Hz, 1H), 7.06 (d, $J$ = 3.9 Hz, 1H).

**$^{13}$C-NMR** (75 MHz, $CDCl_3$): $\delta$ / ppm = 148.8, 142.8, 135.2, 131.3, 131.1, 130.0, 125.0, 122.2, 120.1, 113.5.

**IR** (Diamond-ATR, neat): $\tilde{\nu}$ / cm$^{-1}$ = 3096, 3063, 2921, 1762, 1575, 1537, 1516, 1480, 1437, 1418, 1346, 1287, 1244, 1209, 1102, 1077, 996, 962, 911, 886, 877, 862, 790, 732, 660.

**MS** (EI, 70 eV): m/z (%) = 285 (72), 283 (69), 239 (15), 237 (14), 159 (12), 158 (100), 114 (21), 79 (14).

**HRMS** (EI): calcd. for $[C_{10}H_6BrNO_2S]$: 282.9303; found: 282.9299 ($M^+$).

Synthesis of 5-(2,6-dichlorophenyl)-4-methoxyfuran-2(5H)-one (**8**)

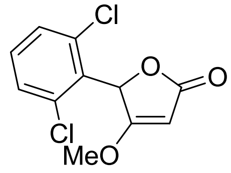


According to **TP1**, injection loop A and B were loaded with solutions of (E)-methyl 3-methoxyacrylate (**6**; 0.45 M containing 0.5 equiv $MgCl_2$) and $Cy_2NLi$ (0.68 M). After injection and in situ trapping metalation the combined streams were collected in a flask containing 2,6-dichlorobenzaldehyde (252 mg; 1.44 mmol; 0.8 equiv) dissolved in THF (2 mL). The reaction mixture was stirred for further 1 h at 25 °C before it was quenched with water (30 mL). The aq. layer was extracted with EtOAc (3×60 mL), the combined organic fractions were dried over anhydrous $Na_2SO_4$, filtrated and the solvent was removed *in vacuo*. Purification by flash column chromatography (silica gel; *i*-hexane:EtOAc = 3:1) afforded **8** as a colorless solid (241 mg; 0.93 mmol; 65%).

**m.p.** (°C): 134.0 – 136.2.

**$^1$H-NMR** (400 MHz, $CDCl_3$): $\delta$ / ppm = 7.33 – 7.30 (m, 1H), 7.23 – 7.19 (m, 2H), 6.50 (d, $J$ = 1.5 Hz, 1H), 5.22 (d, $J$ = 1.5 Hz, 1H), 3.82 (s, 3H).

**$^{13}$C-NMR** (101 MHz, $CDCl_3$): $\delta$ / ppm = 180.0, 172.3, 137.4, 136.2, 131.1, 130.3, 128.7, 127.8, 90.6, 76.3, 59.8.

**IR** (Diamond-ATR, neat): $\tilde{\nu}$ / cm$^{-1}$ = 3117, 2925, 1747, 1628, 1580, 1563, 1436, 1346, 1283, 1246, 1189, 1148, 1094, 1035, 988, 941, 888, 836, 769, 734, 674.

**MS** (EI, 70 eV): m/z (%) = 260 (23), 258 (31), 175 (13), 173 (15), 85 (100).

**HRMS** (EI): calcd. for [$C_{11}H_8Cl_2O_3$]: 257.9851; found: 257.9842 ($M^+$).

Synthesis of 4-(dimethylamino)-5-(4-(trifluoromethyl)phenyl)furan-2(5H)-one (**11**)

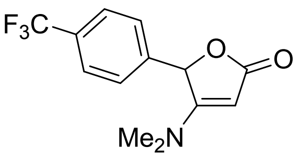

According to **TP1**, injection loop A and B were loaded with solutions of (E)-ethyl 3-(dimethylamino)acrylate (**9**; 0.44 M containing 0.5 equiv $MgCl_2$) and $Cy_2NLi$ (0.66 M). After injection and in situ trapping metalation the combined streams were collected in a flask containing 4-(trifluoromethyl)benzaldehyde (246 mg; 1.41 mmol; 0.8 equiv) dissolved in THF (2 mL). The reaction mixture was stirred for further 30 min at 25 °C before it was quenched with water (20 mL). The aq. layer was extracted with EtOAc (3×60 mL), the combined organic fractions were dried over anhydrous $Na_2SO_4$, filtrated and the solvent was removed *in vacuo*. Purification by flash column chromatography (silica gel; EtOAc) afforded **11** as a yellow solid (238 mg; 0.88 mmol; 62%).

**m.p.** (°C): 146.5 – 148.3.

**$^1$H-NMR** (400 MHz, $CDCl_3$): $\delta$ / ppm = 7.64 (d, $J$ = 8.1 Hz, 2H), 7.45 (d, $J$ = 8.0 Hz, 2H), 5.76 (s, 1H), 4.79 (s, 1H), 2.79 (br, 6H).

**$^{13}$C-NMR** (101 MHz, $CDCl_3$): $\delta$ / ppm = 173.5, 169.7, 139.1, 131.8 (q, $J$ = 33.2 Hz), 128.3, 126.2 (q, $J$ = 3.0 Hz), 123.7 (q, $J$ = 272.6 Hz), 83.3, 79.2, 40.3.

**IR** (Diamond-ATR, neat): $\tilde{\nu}$ / cm$^{-1}$ = 3116, 2925, 2855, 1811, 1779, 1714, 1617, 1518, 1478, 1443, 1425, 1411, 1331, 1306, 1272, 1201, 1163, 1106, 1068, 1009, 973, 903, 872, 850, 839, 786, 769, 753, 718, 678.

**MS** (EI, 70 eV): m/z (%) = 272 (12), 271 (84, $M^+$), 97 (11), 69 (100).

**HRMS** (EI): calcd. for [$C_{13}H_{12}F_3NO_2$]: 271.0820; found: 271.0821 ($M^+$).

Synthesis of (E)-ethyl 3-(dimethylamino)-3-(4-(trifluoromethyl)phenyl)acrylate (**13**)

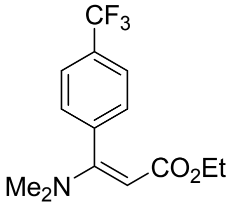


According to **TP1**, injection loop A and B were loaded with solutions of (E)-ethyl 3-(dimethylamino)acrylate (**9**; 0.45 M containing 0.5 equiv $ZnCl_2\cdot 2LiCl$) and $Cy_2NLi$ (0.69 M). After injection and in situ trapping metalation the combined streams were collected in a flask containing 4-iodobenzotrifluoride (392 mg; 1.44 mmol; 0.8 equiv), $Pd(dba)_2$ (21 mg; 2 mol%) and tfp (17 mg; 4 mol%) dissolved in THF (2 mL). The reaction mixture was stirred overnight at 25 °C before it was quenched with water (25 mL). The aq. layer was extracted with EtOAc (3×70 mL), the combined organic fractions were dried over anhydrous $Na_2SO_4$, filtrated and the solvent was removed *in vacuo*. Purification by flash column chromatography (silica gel; *i*-hexane:EtOAc = 5:1) afforded **13** as a yellow solid (291 mg; 1.01 mmol; 70%).

**m.p.** (°C): 83.1 – 84.7.

**$^1$H-NMR** (400 MHz, $CDCl_3$): $\delta$ / ppm = 7.64 (d, $J$ = 8.0 Hz, 2H), 7.31 (d, $J$ = 7.9 Hz, 2H), 4.80 (s, 1H), 3.87 (q, $J$ = 7.1 Hz, 2H), 2.76 (s, 6H), 1.04 (t, $J$ = 7.1 Hz, 3H).

**$^{13}$C-NMR** (101 MHz, $CDCl_3$): $\delta$ / ppm = 167.7, 161.9, 140.7, 130.4 (q, $J$ = 32.2 Hz), 128.7, 125.3 (q, $J$ = 4.0 Hz), 124.1 (q, $J$ = 272.6 Hz), 87.7, 58.6, 40.3, 14.3.

**IR** (Diamond-ATR, neat): $\tilde{\nu}$ / $cm^{-1}$ = 2983, 2933, 1680, 1618, 1563, 1519, 1481, 1448, 1401, 1351, 1320, 1243, 1210, 1145, 1116, 1090, 1061, 1043, 1015, 998, 911, 856, 840, 796, 755, 721, 687.

**MS** (EI, 70 eV): m/z (%) = 287 (57, $M^+$), 286 (100), 268 (11), 258 (30), 242 (98), 240 (14), 215 (23), 214 (70), 212 (19), 199 (16), 184 (14), 173 (33), 171 (24), 151 (22), 145 (15), 72 (18), 44 (32), 42 (11).

**HRMS** (EI): calcd. for $[C_{14}H_{15}F_3NO_2]$: 286.1055; found: 286.1043 ($M^+$ - H).

ethyl 5'-bromo-2'-fluorobiphenyl-4-carboxylate (**3a**)

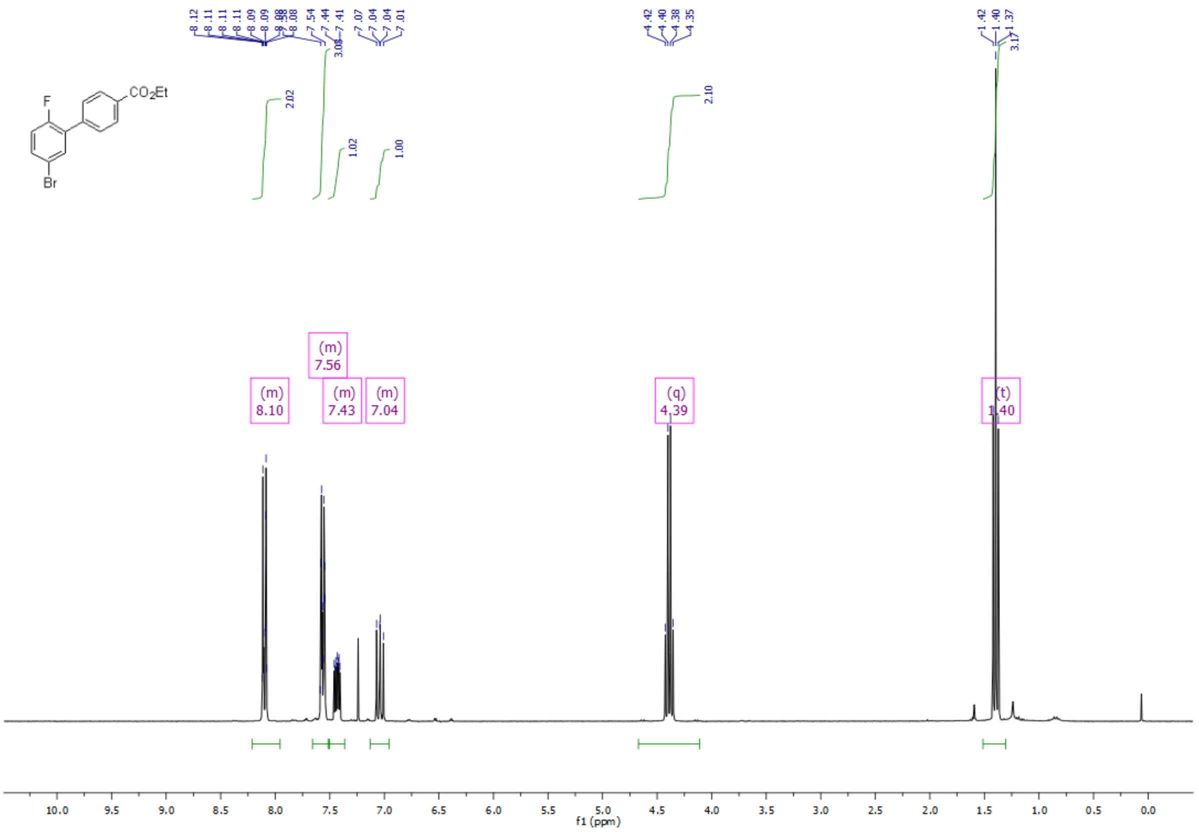


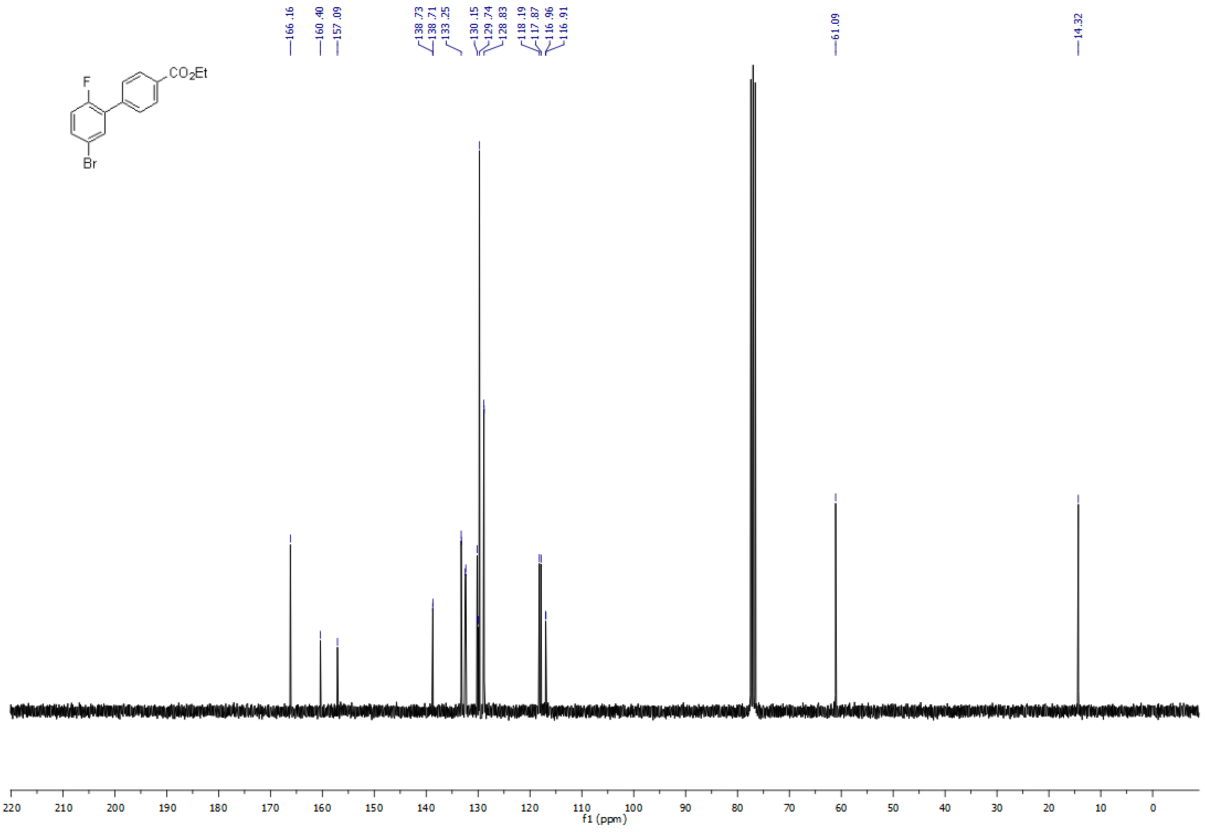

5-bromo-2-fluoro-3'-methoxybiphenyl (**3b**)

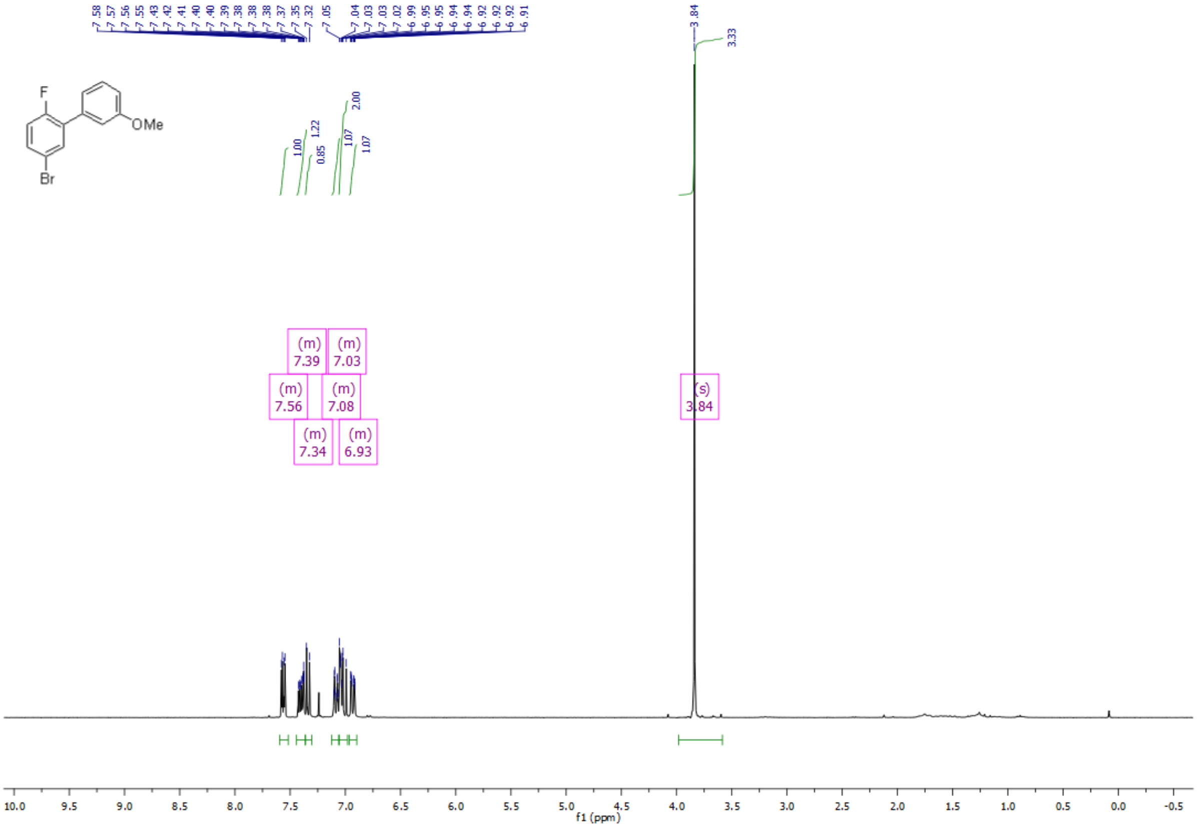


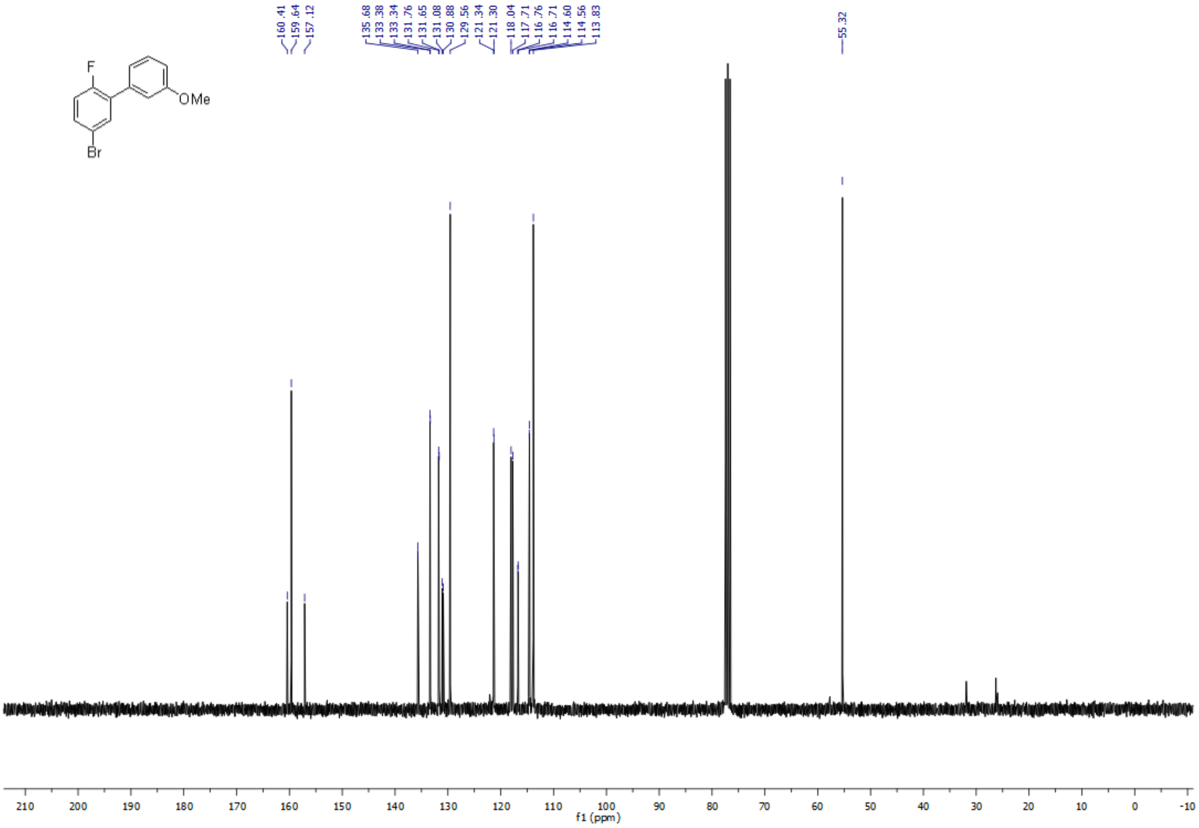

2-chloro-6-fluoro-4'-methoxybiphenyl (**3c**)

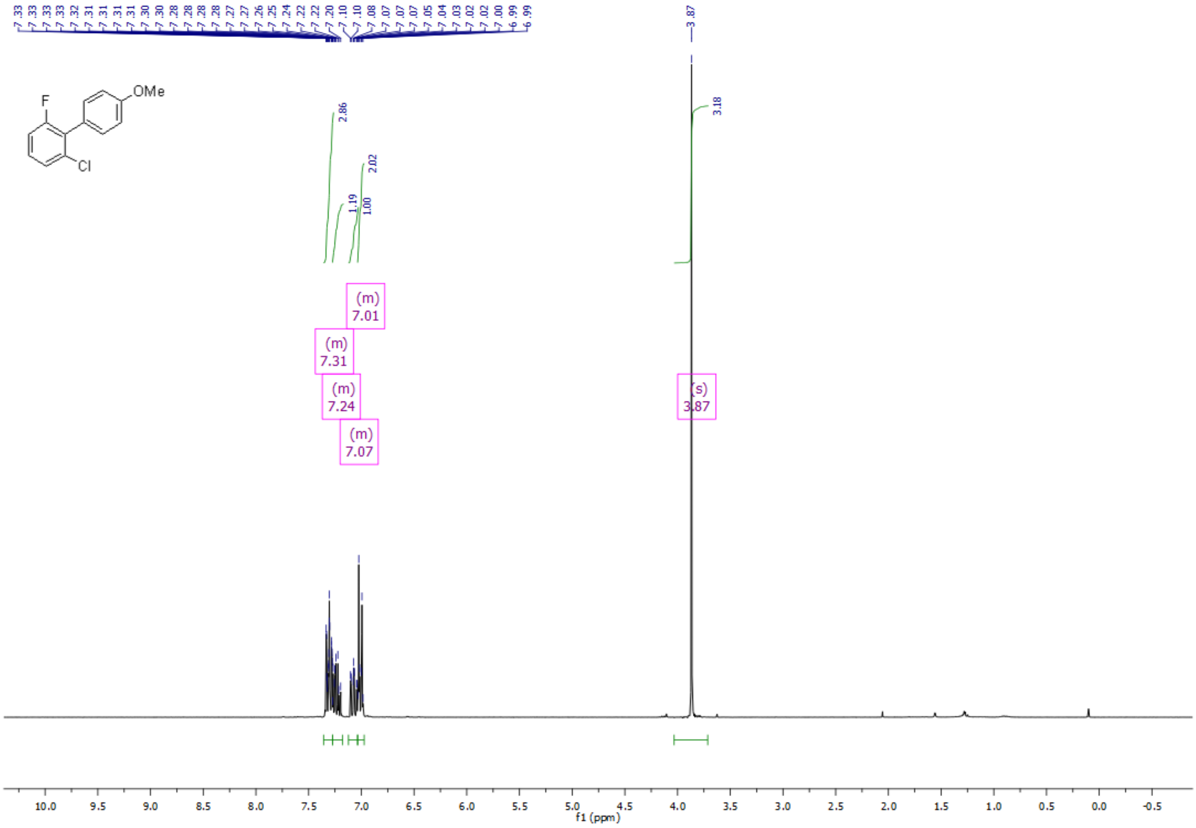


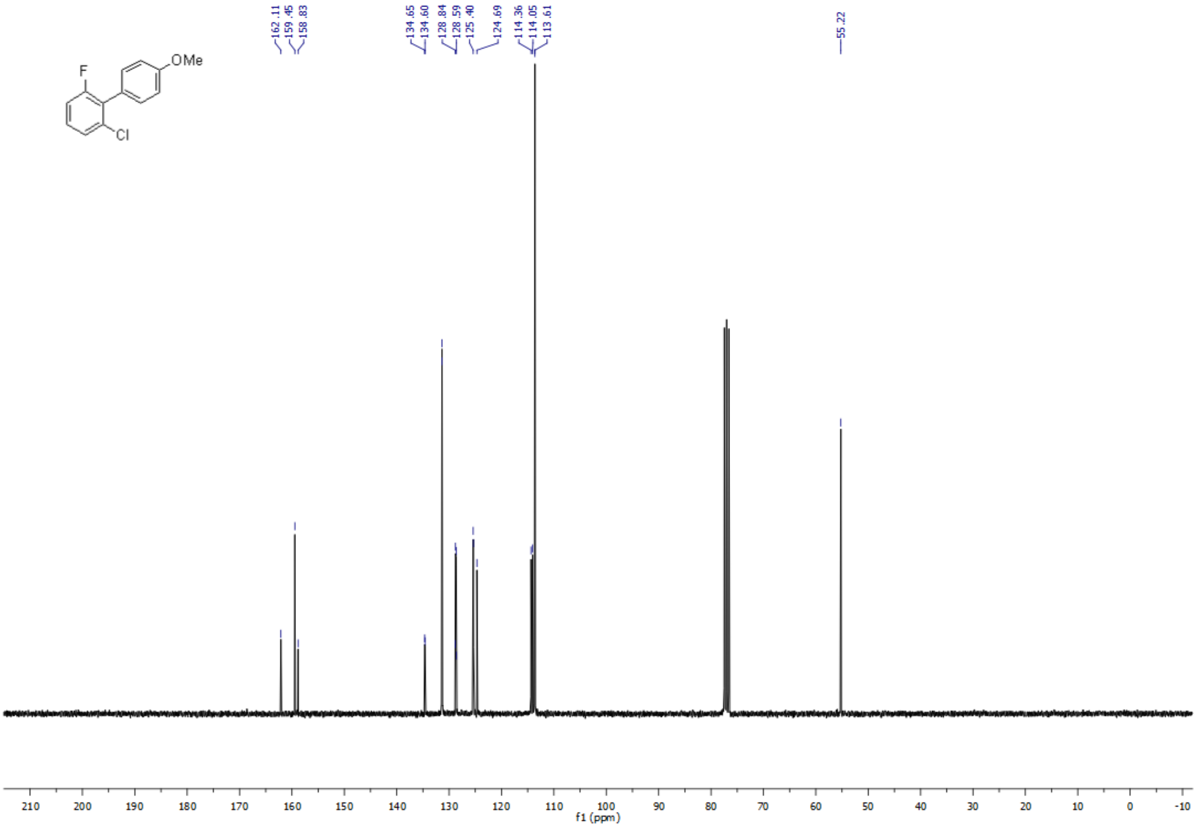

(2-chloro-6-fluorophenyl)(phenyl)sulfane (**3d**)

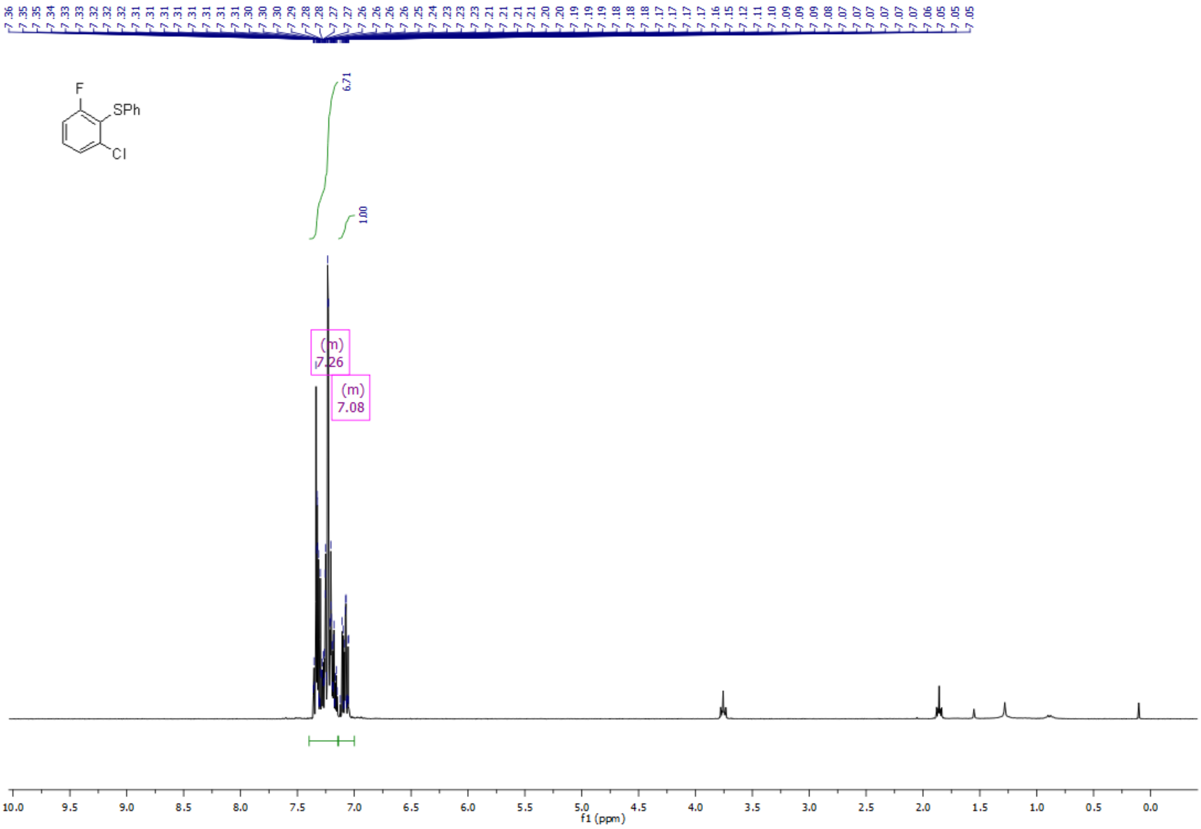


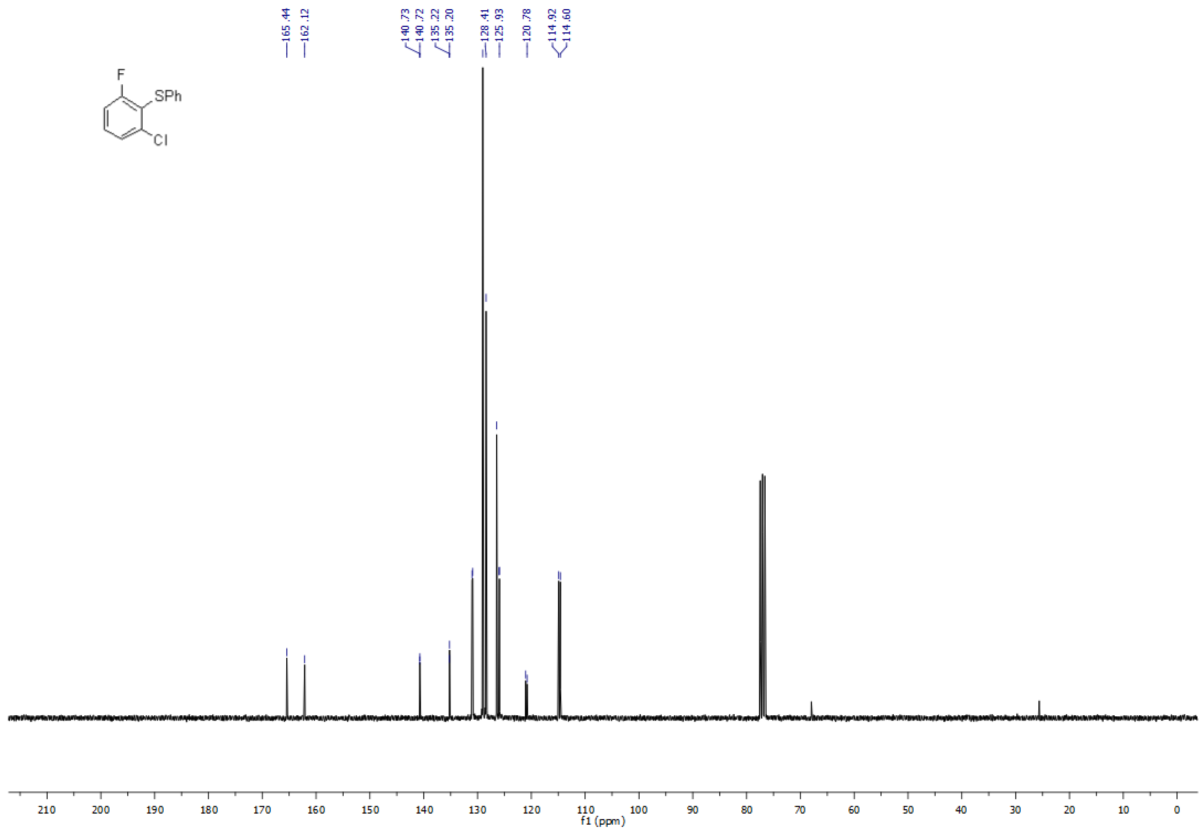

ethyl 2',6'-difluorobiphenyl-4-carboxylate (**3e**)

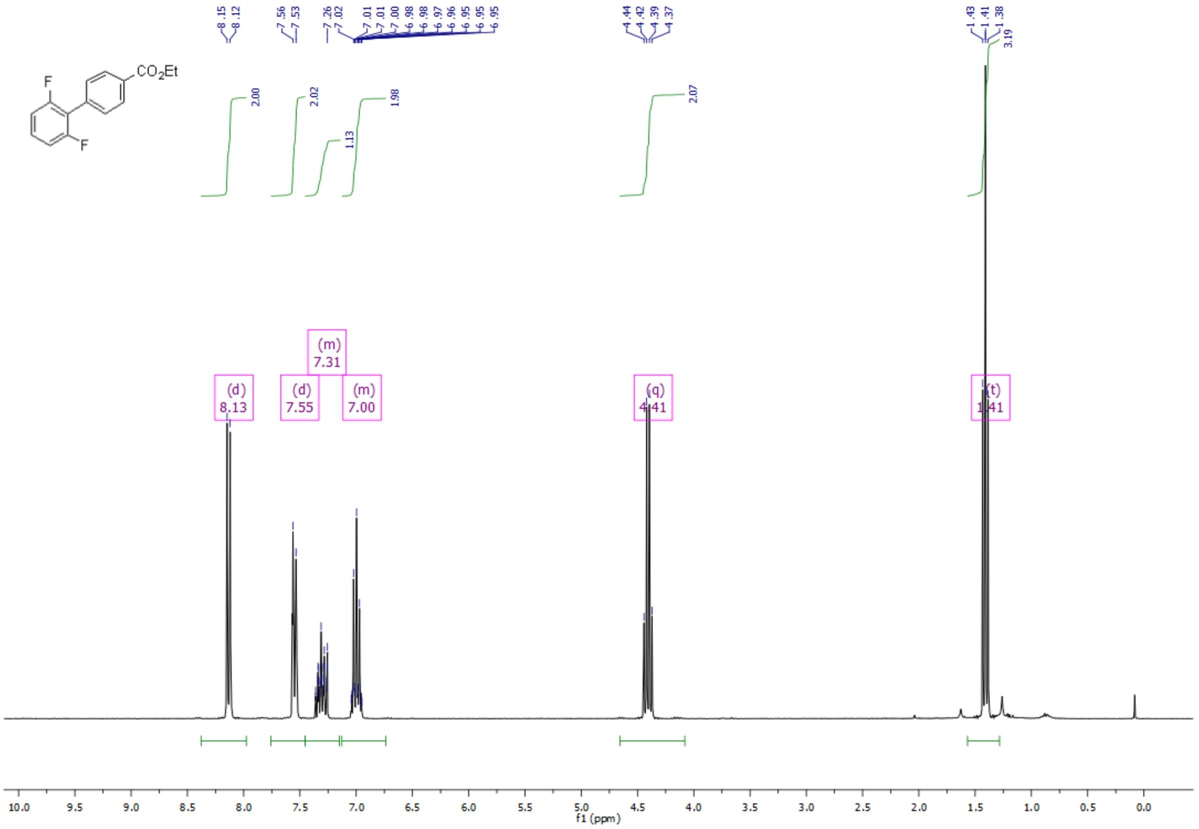


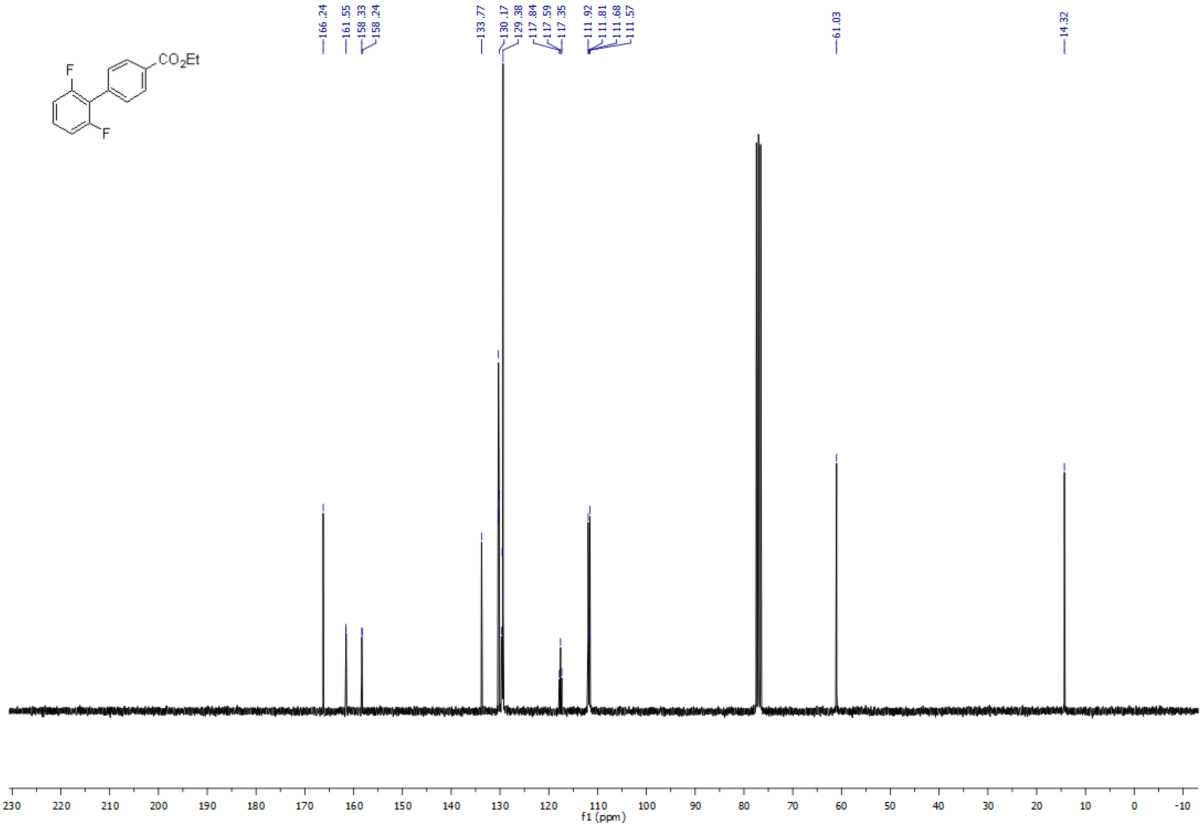

ethyl 2,6-difluorobenzoate (**3f**)

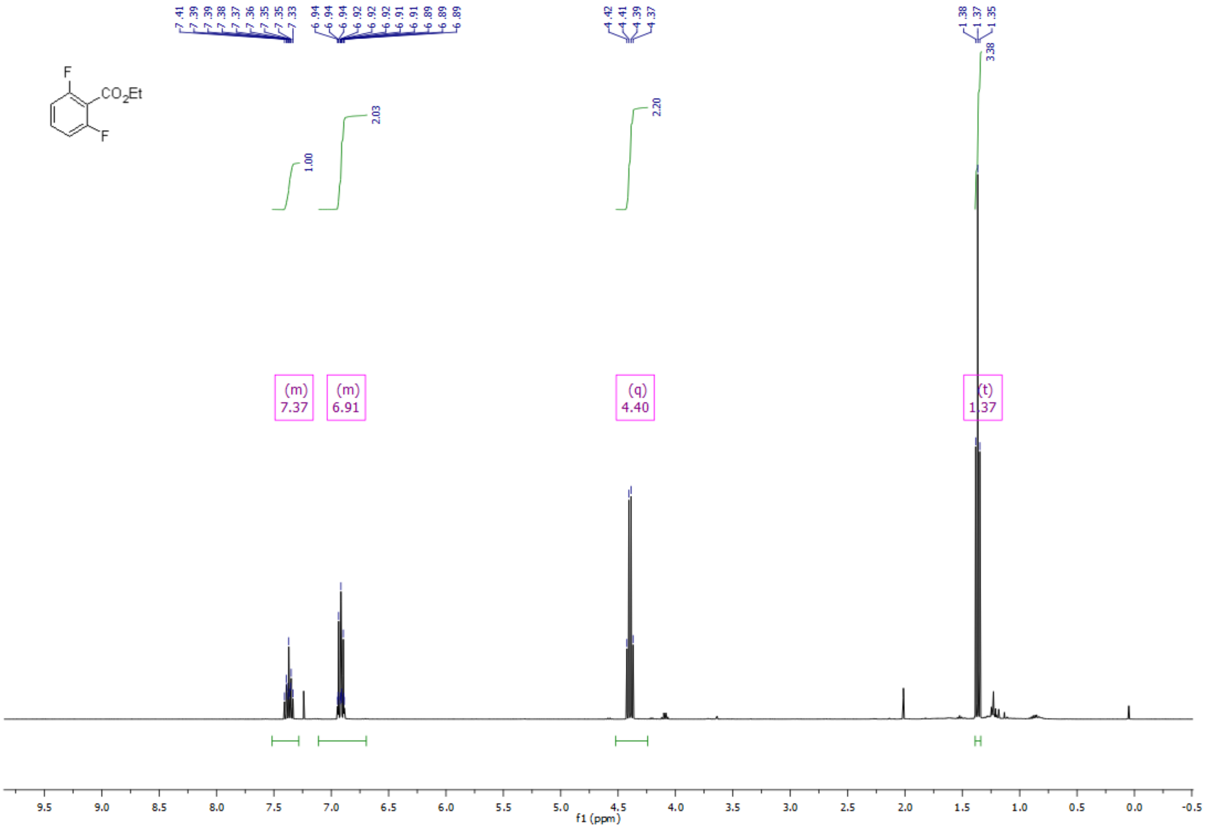

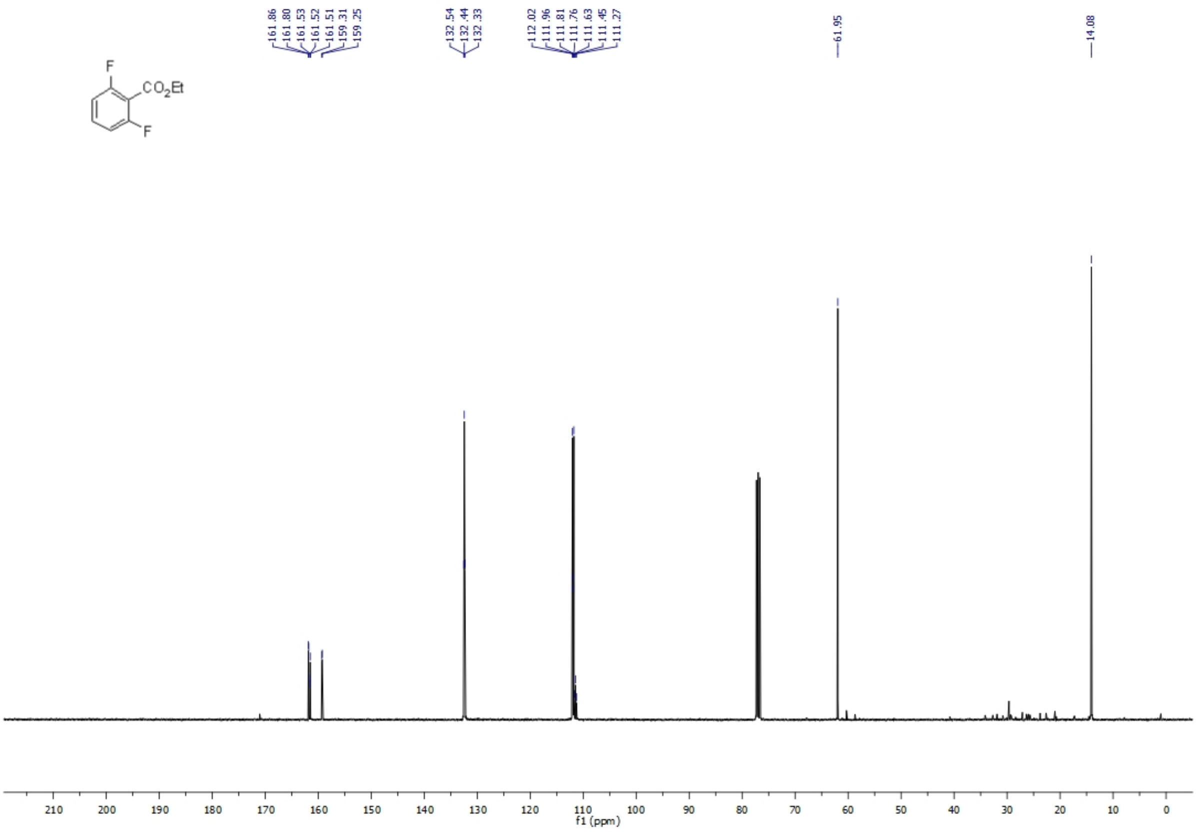

triethyl 5-bromobiphenyl-2,4,4'-tricarboxylate (**3g**)

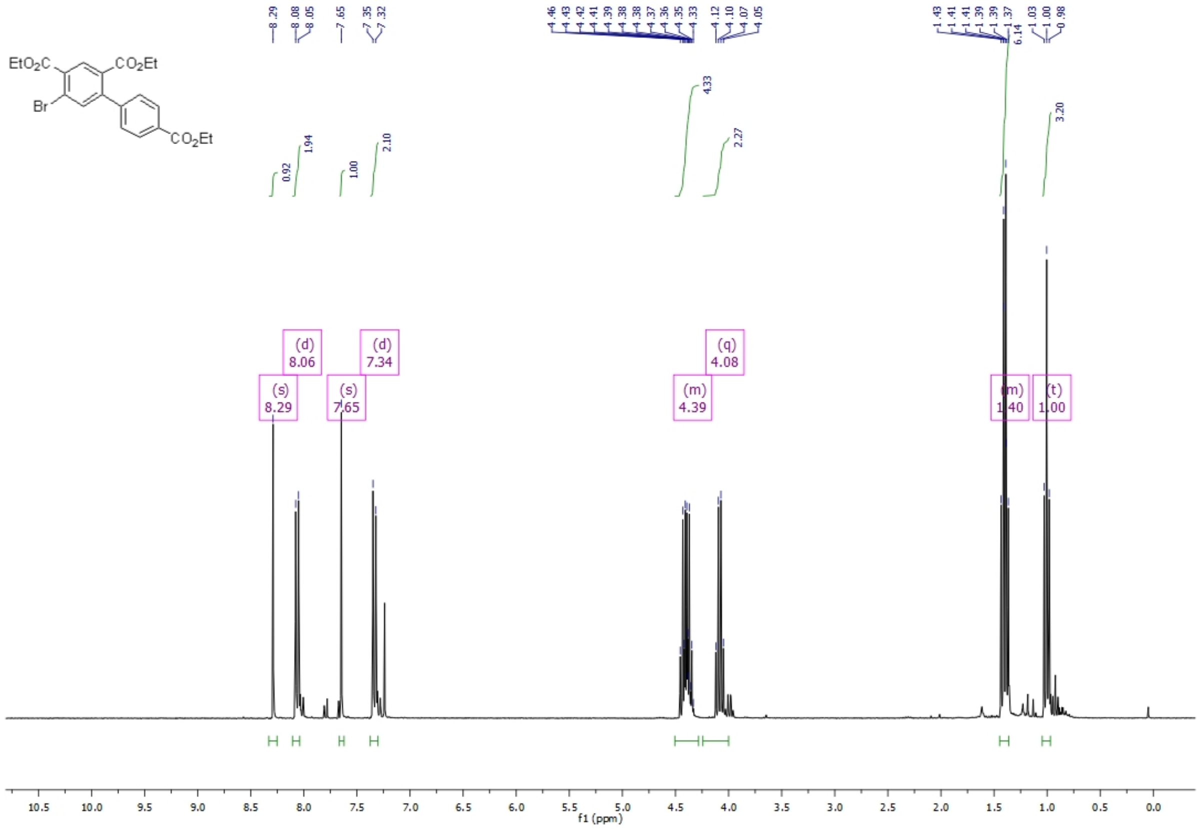


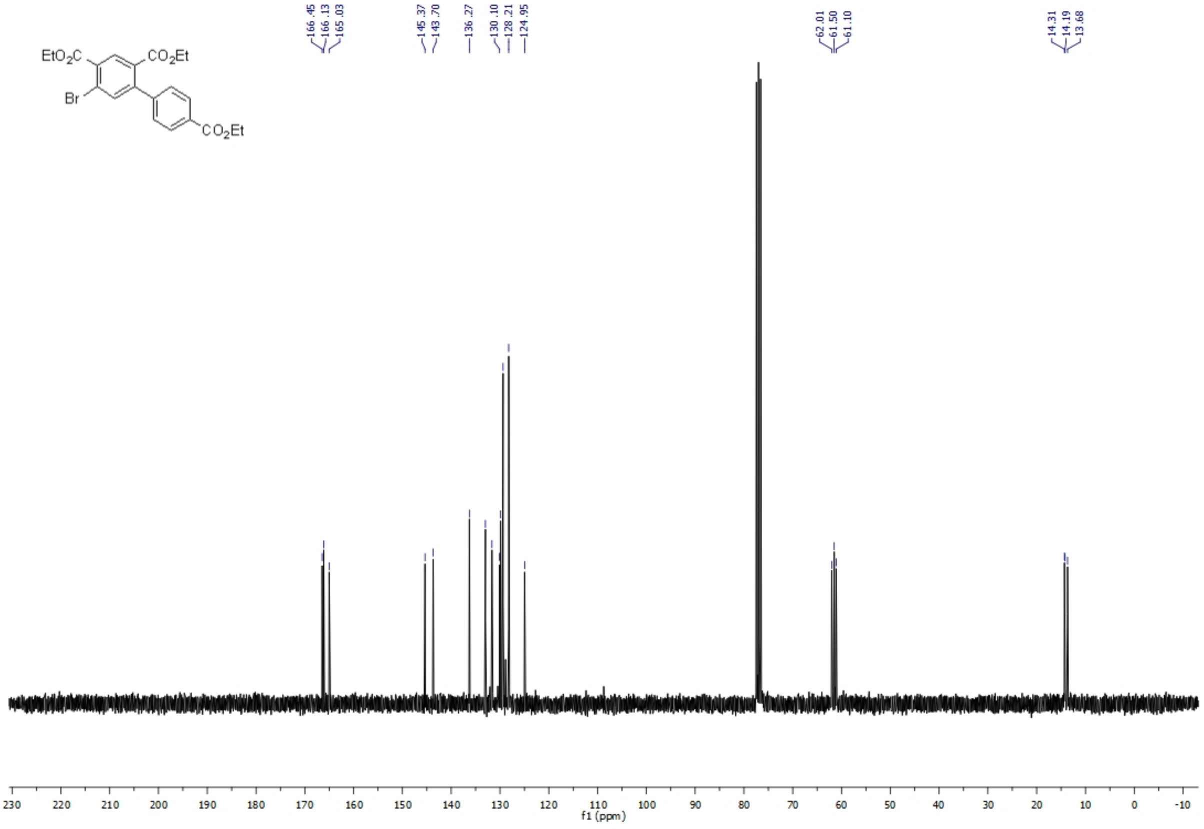

4'-methoxy-5-(trifluoromethyl)biphenyl-2-carbonitrile (**3h**)

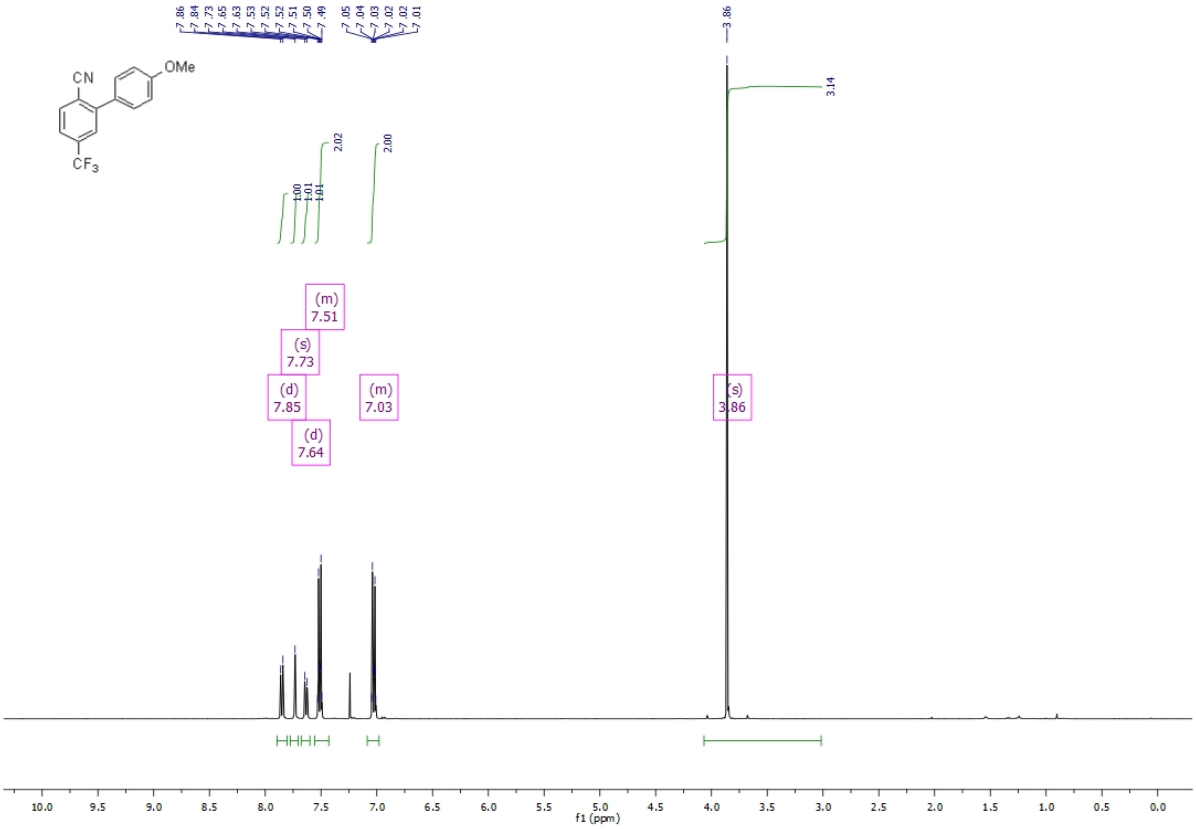


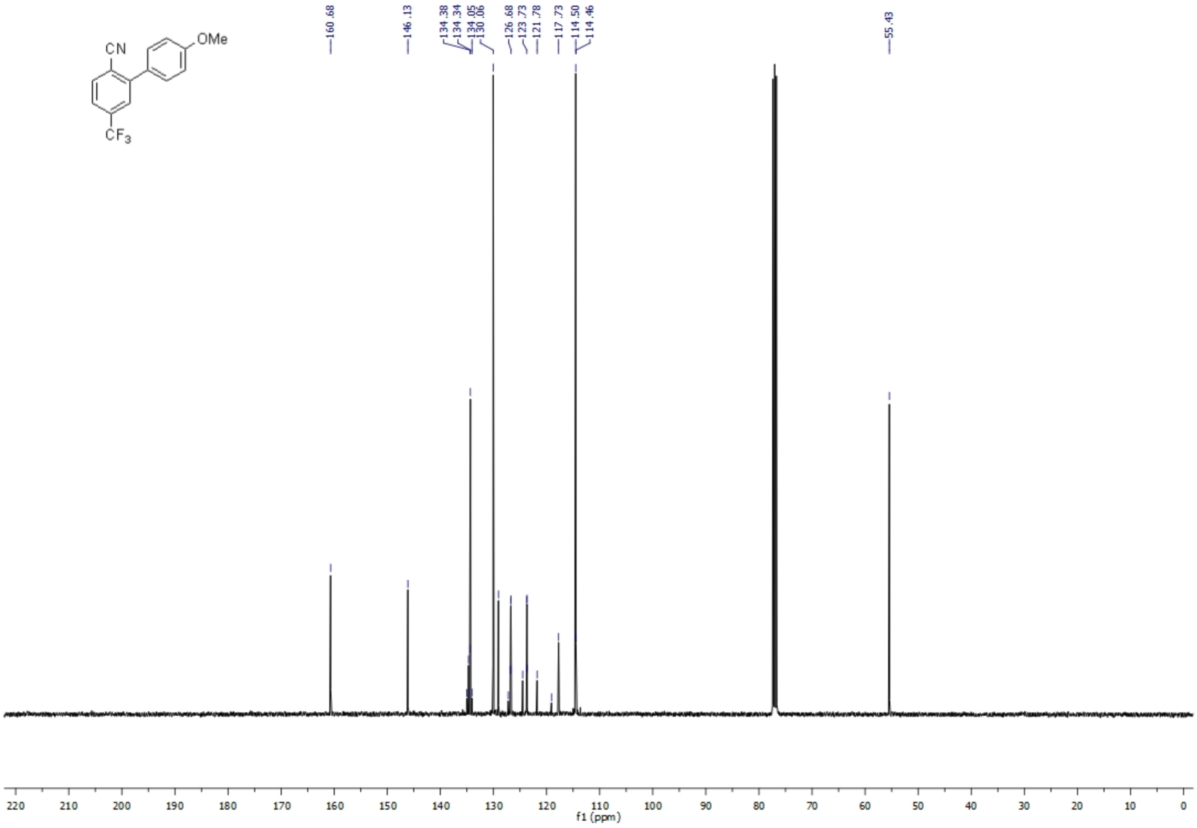

4-chloro-2-fluoro-3'-methoxybiphenyl-3-carbonitrile (**3i**)

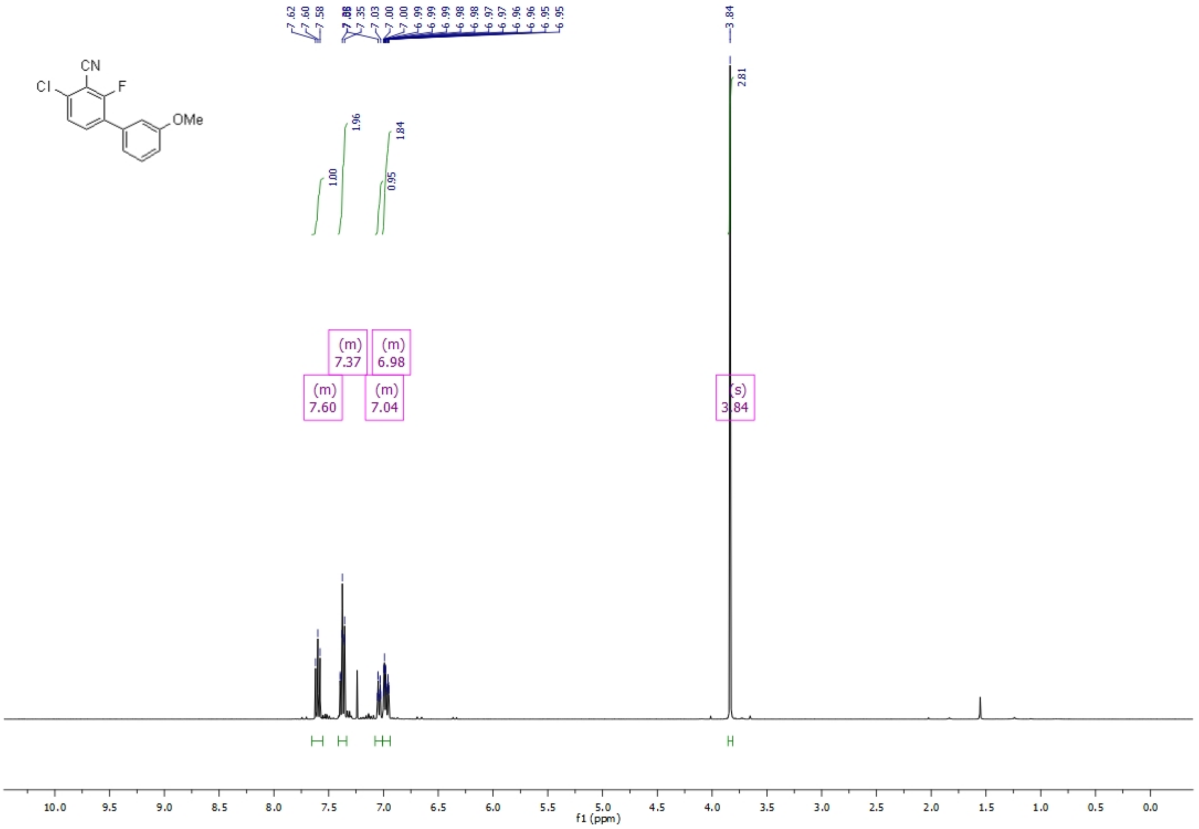


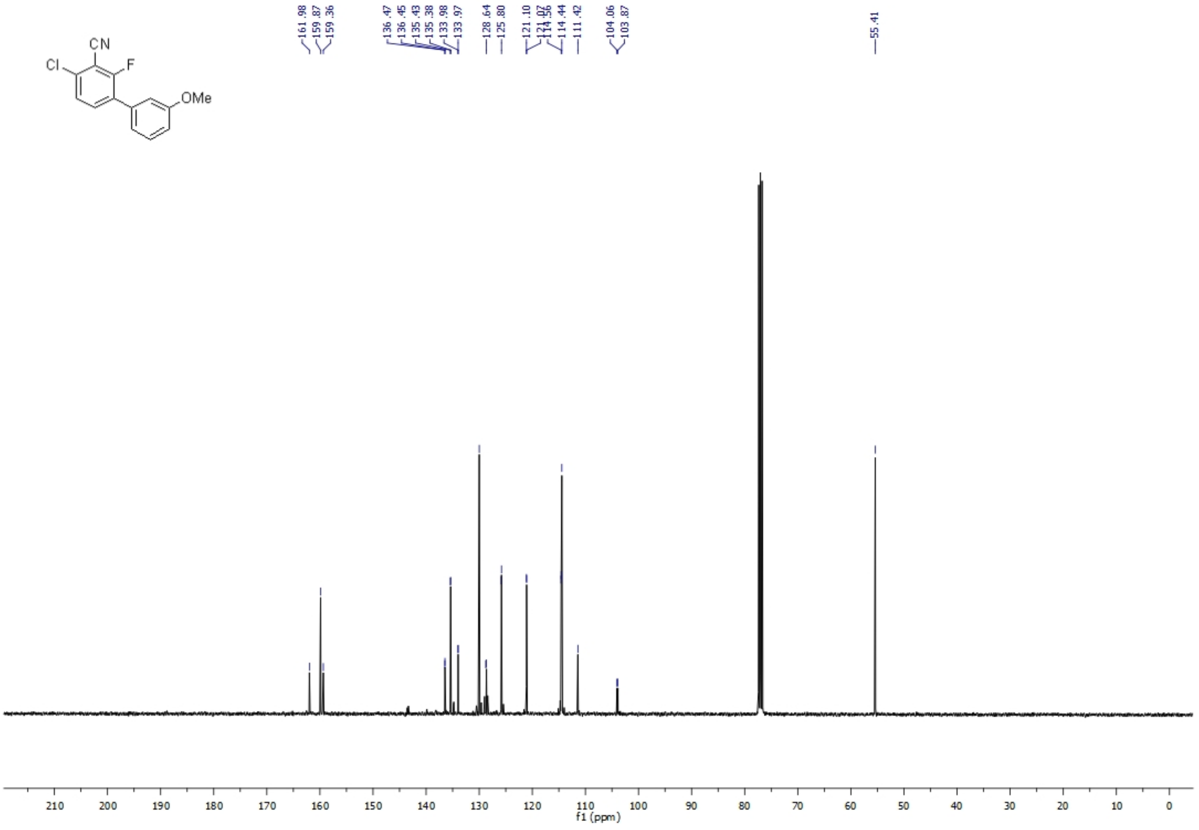

4-chloro-2-fluorobiphenyl-3,4'-dicarbonitrile (**3j**)

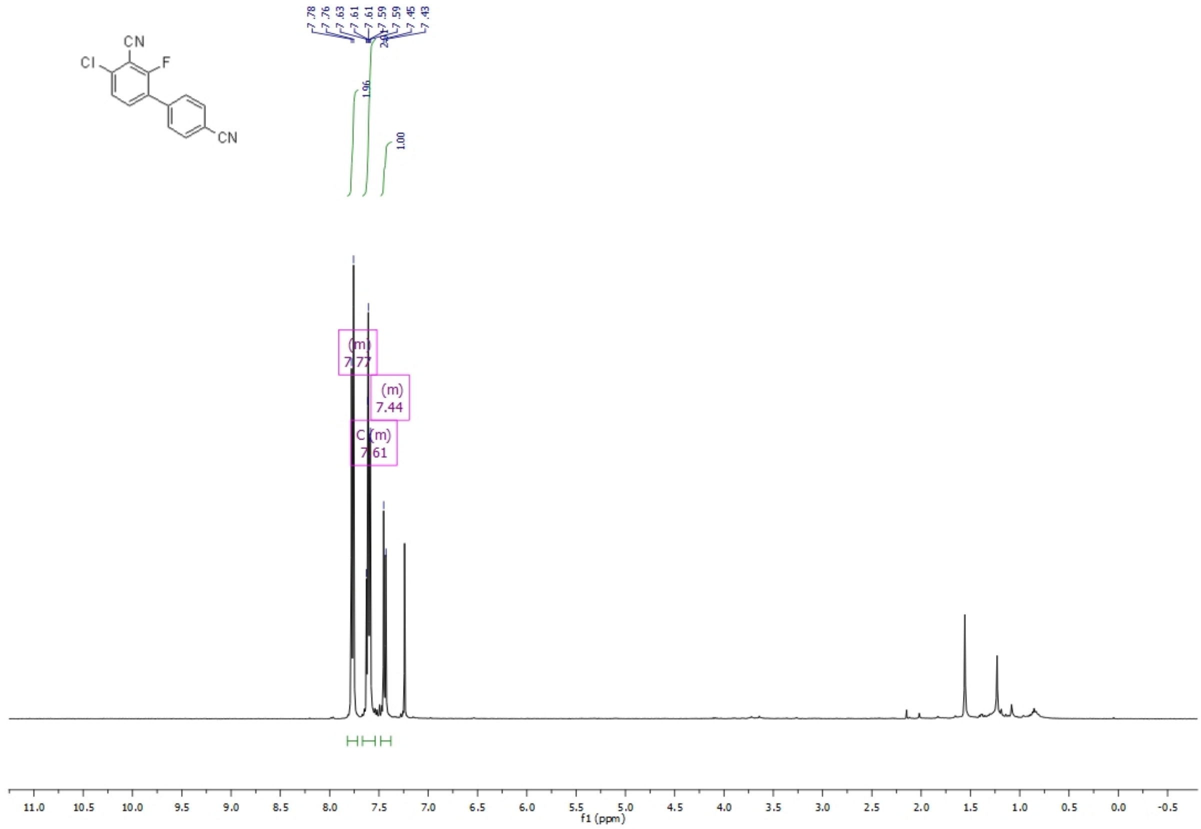


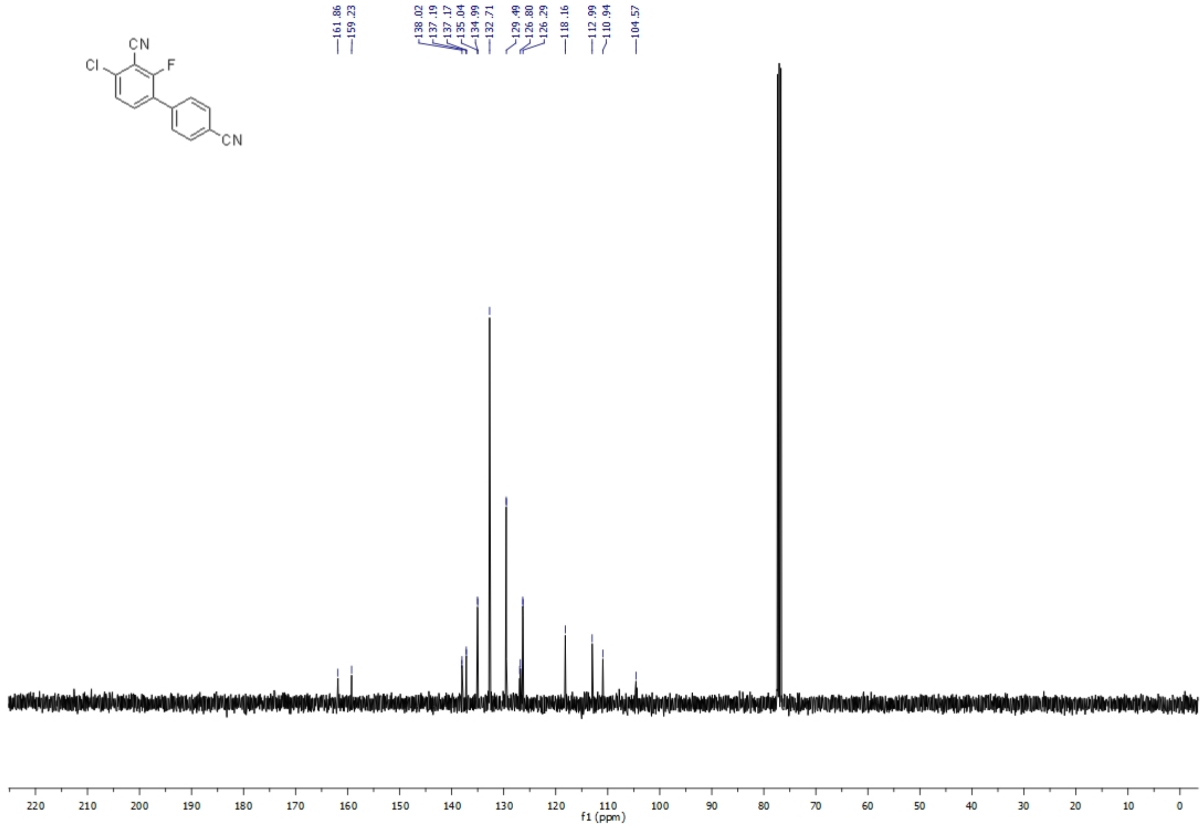

ethyl 4-(2-fluoropyridin-3-yl)benzoate (**5a**)

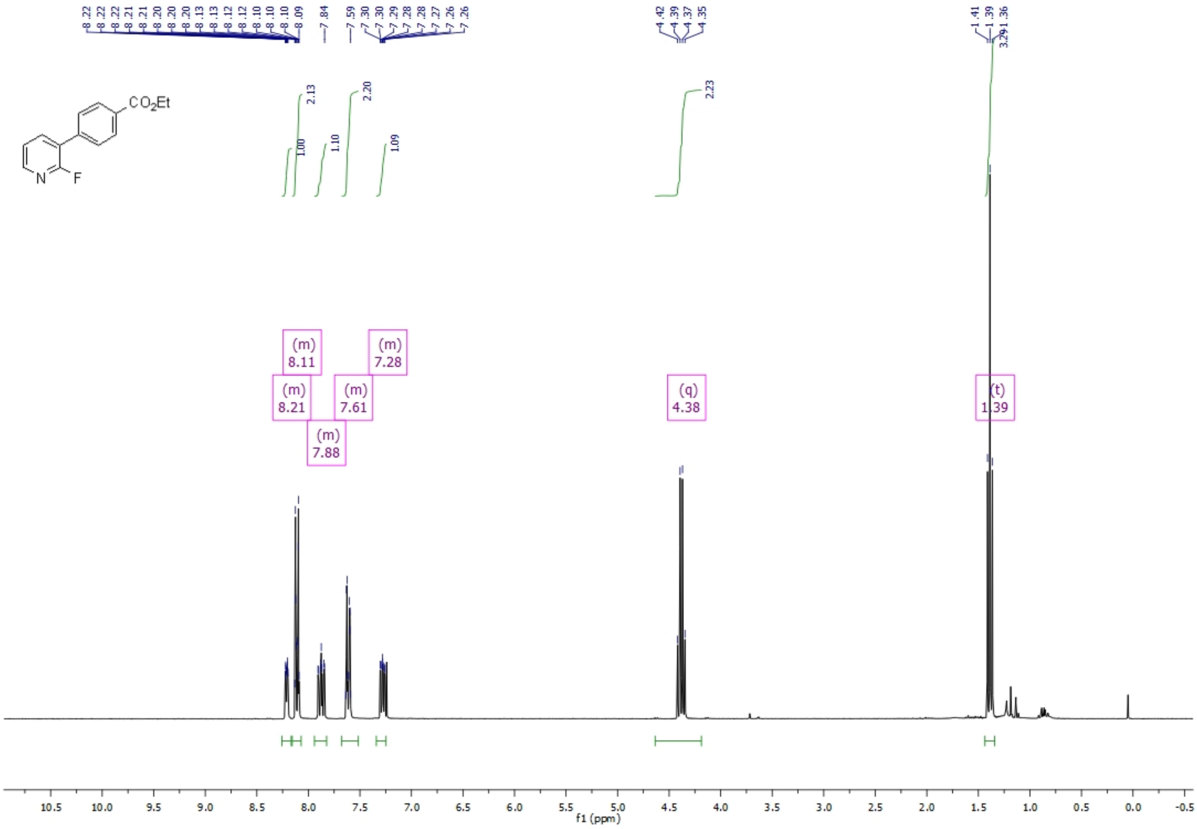


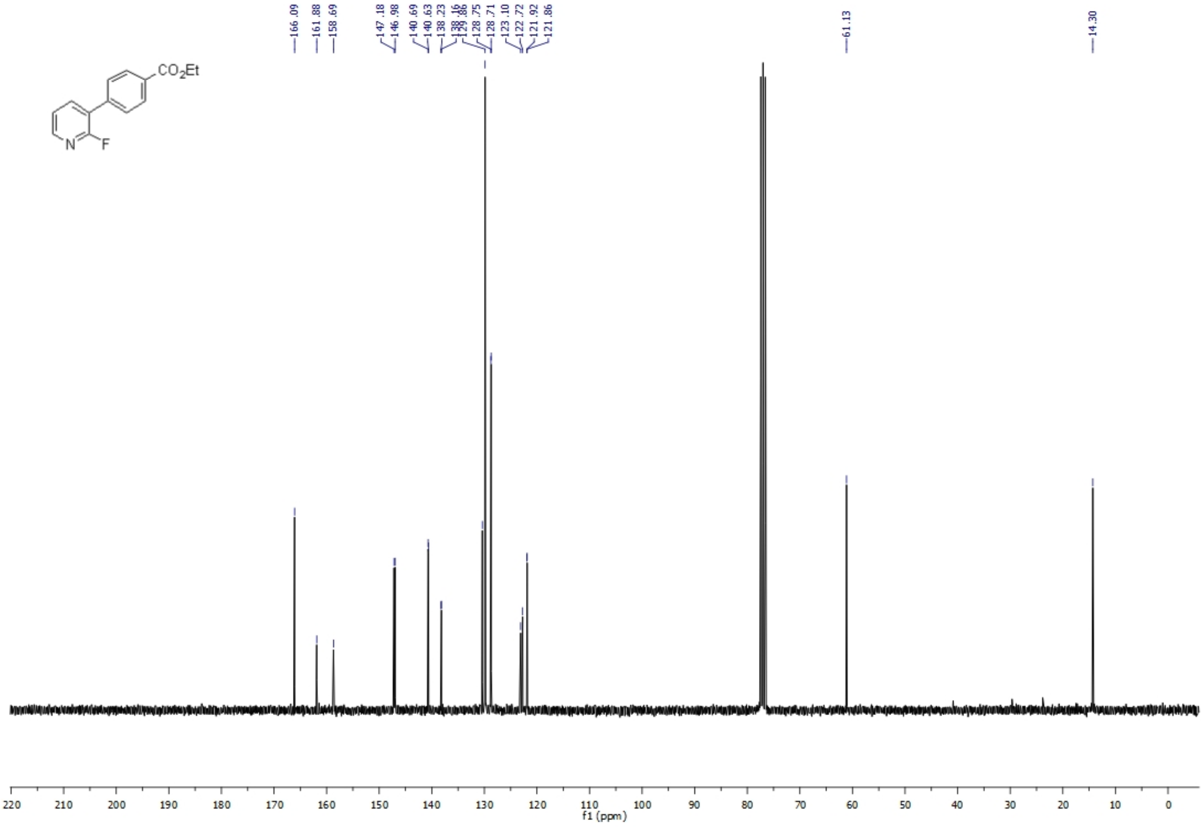

2-fluoro-3-(methylthio)pyridine (**5b**)

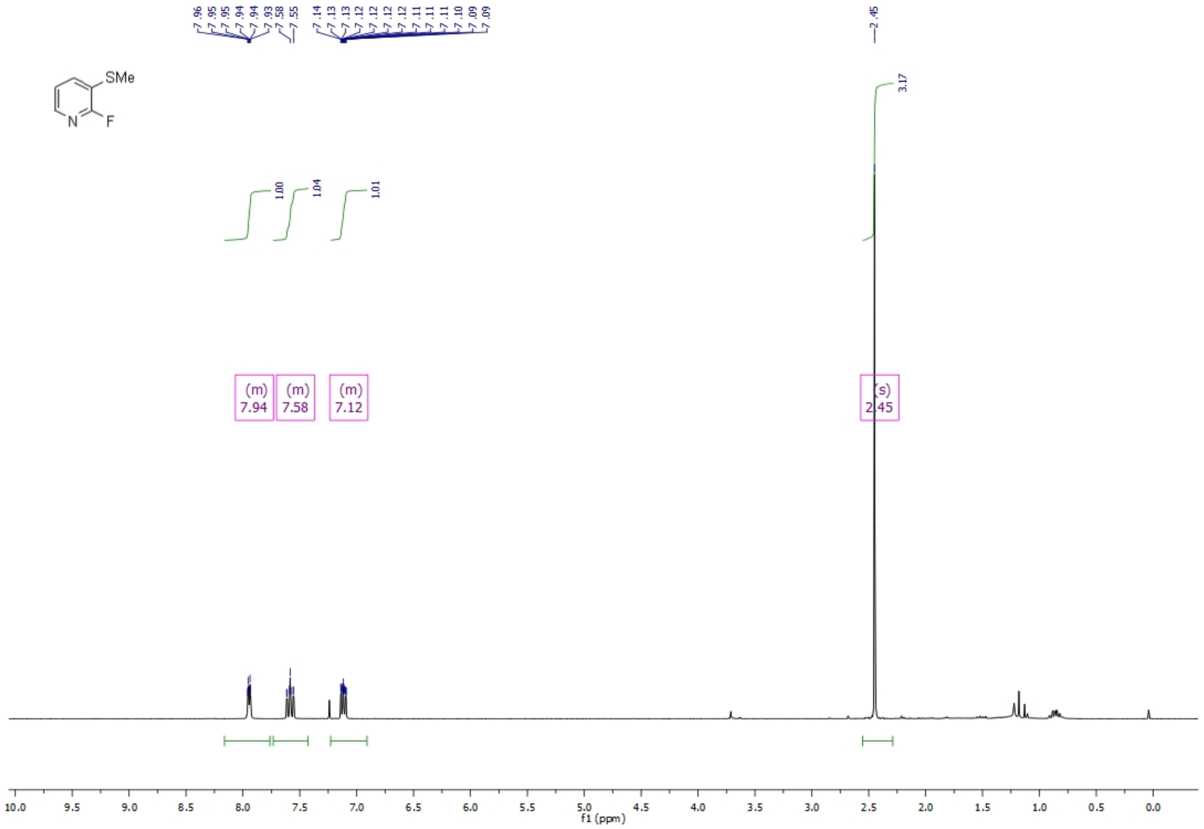


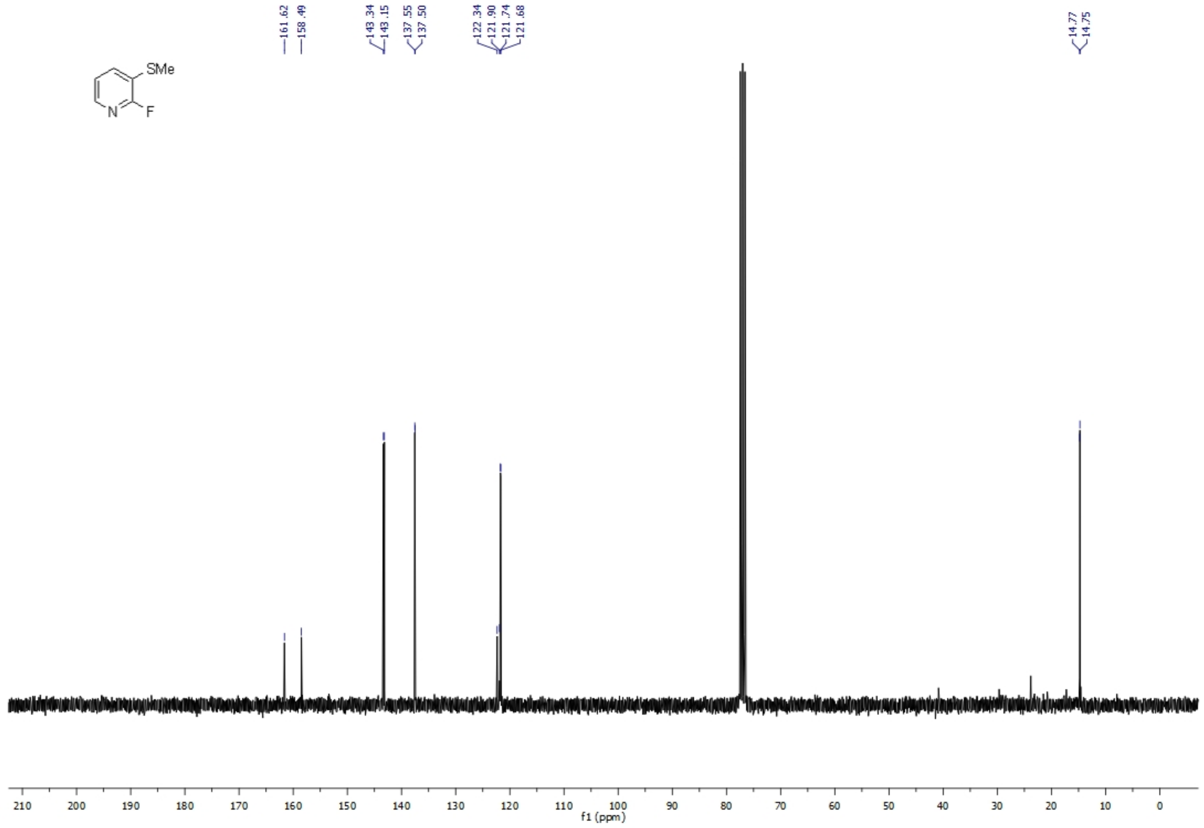

ethyl 4-(2,6-dibromopyridin-4-yl)benzoate (**5c**)

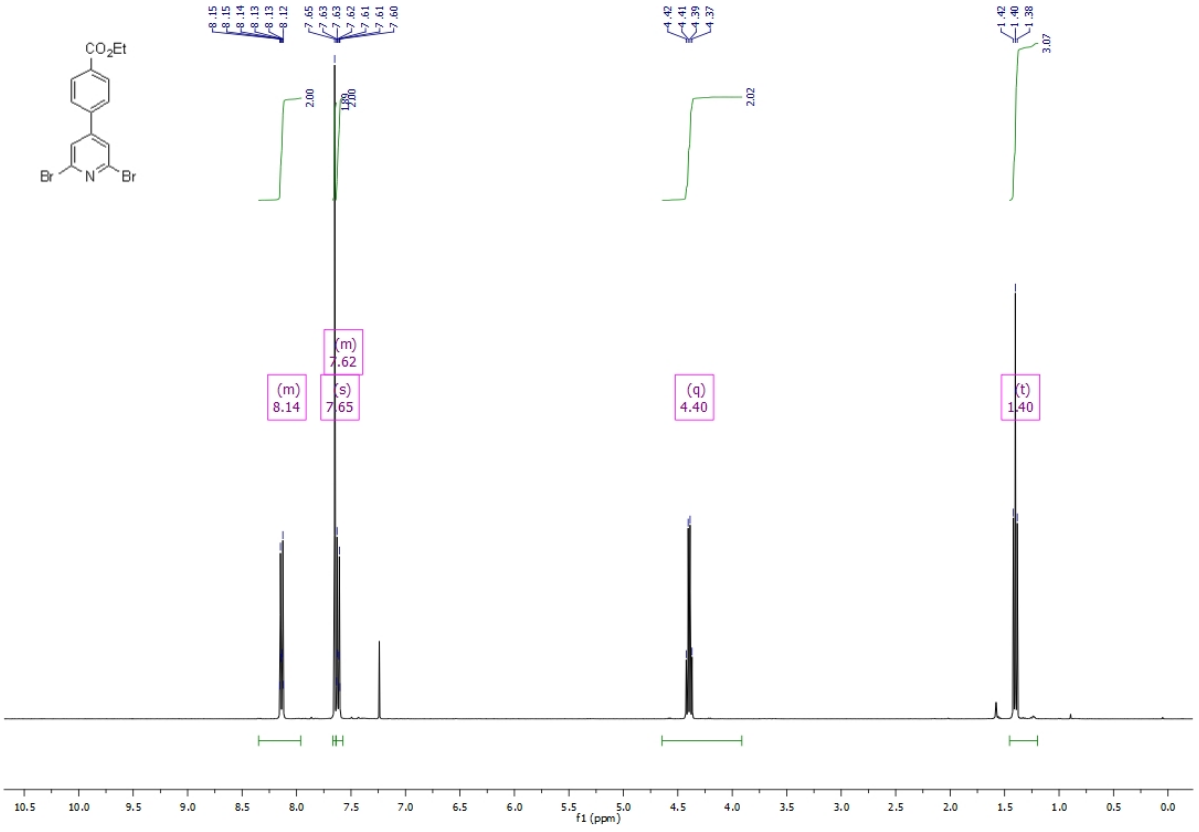

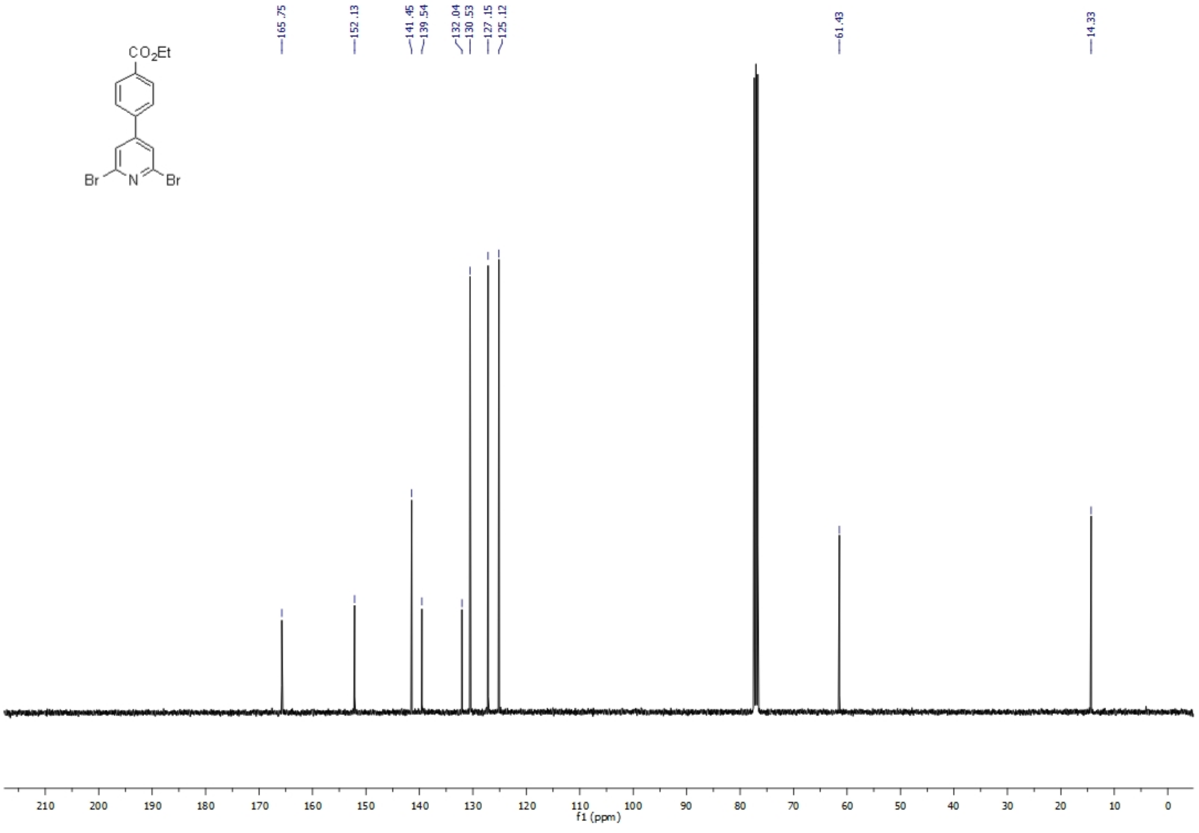

ethyl 2-chloro-4-(cyclohex-2-enyl)nicotinate (**5d**)

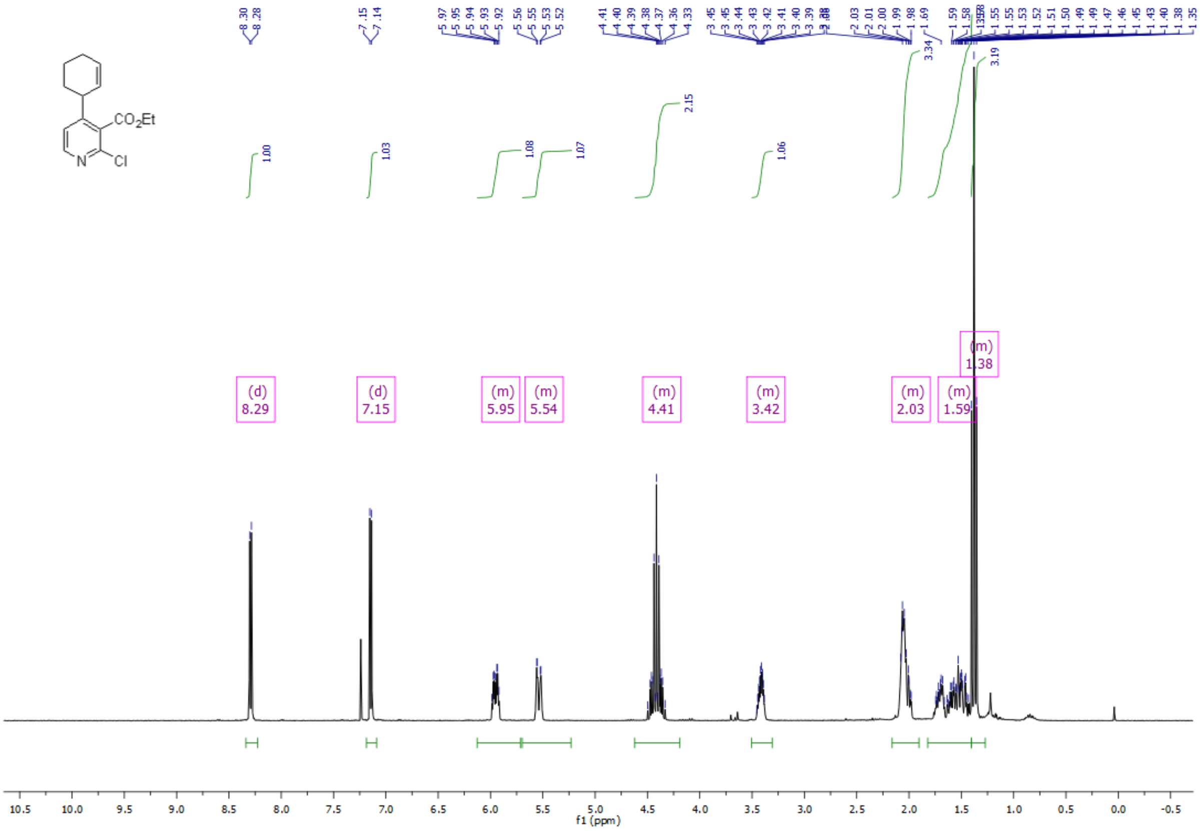


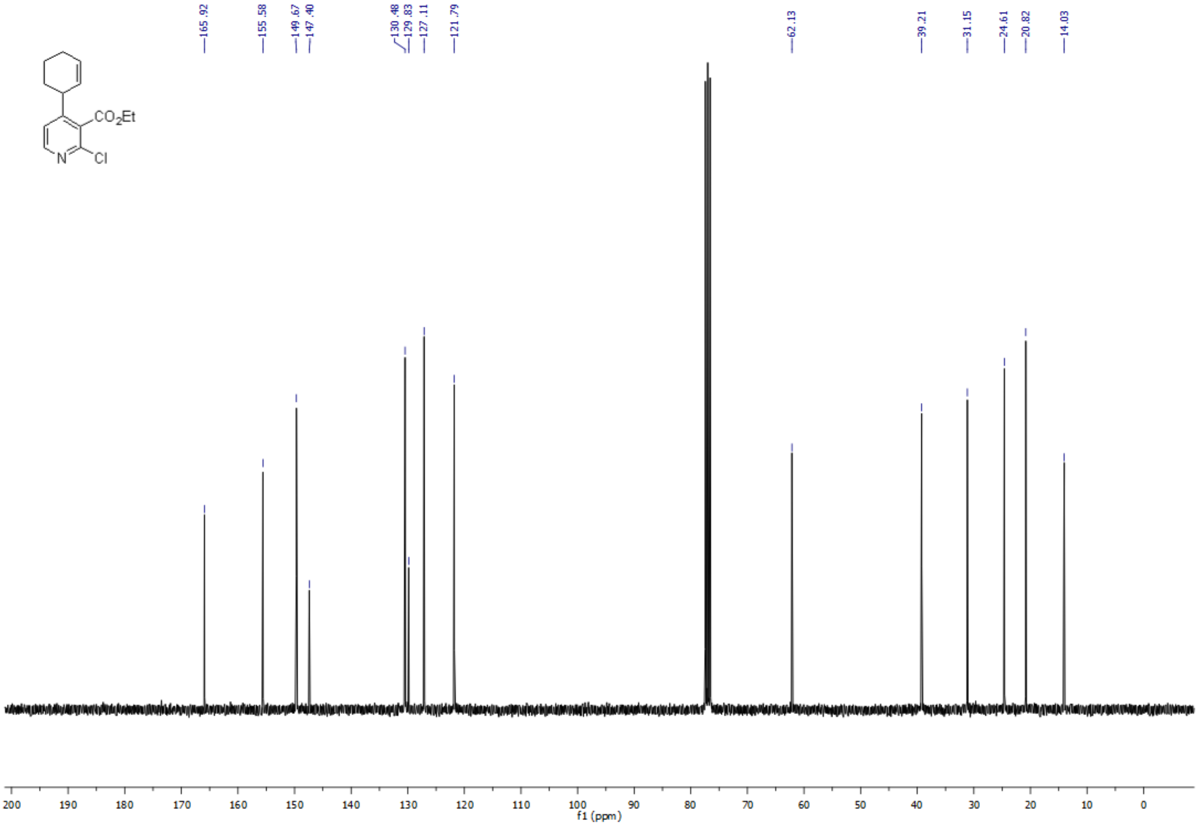

2,3-dichloro-5-(3-methoxyphenyl)pyrazine (**5e**)

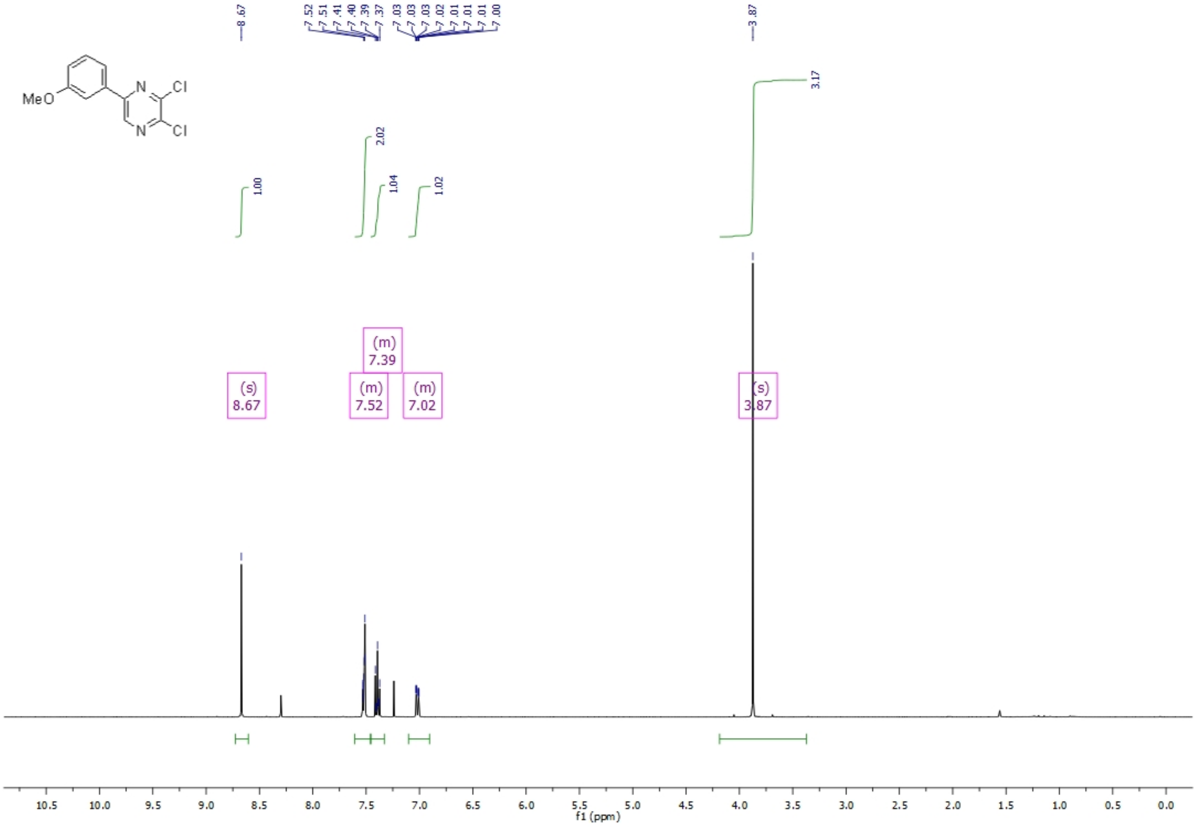


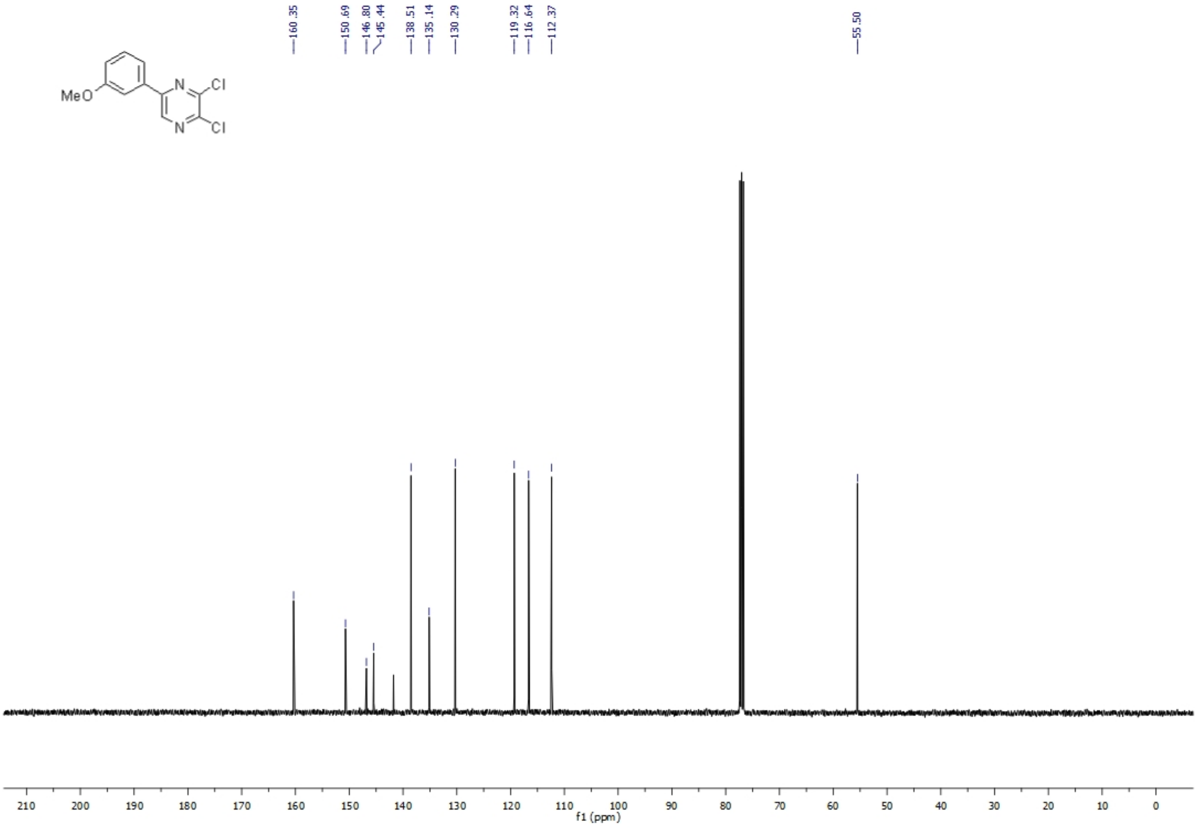

(4-chlorophenyl)(1-methyl-1H-pyrazol-5-yl)methanol (**5f**)

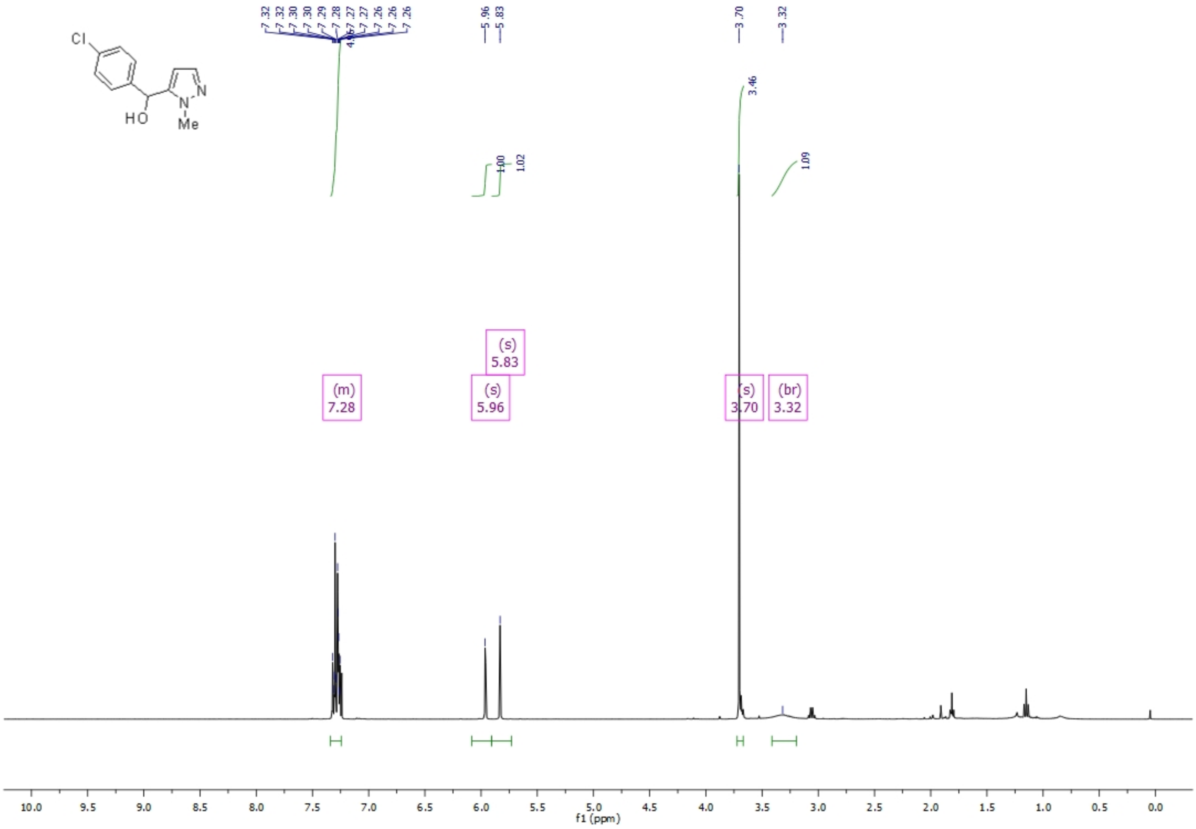

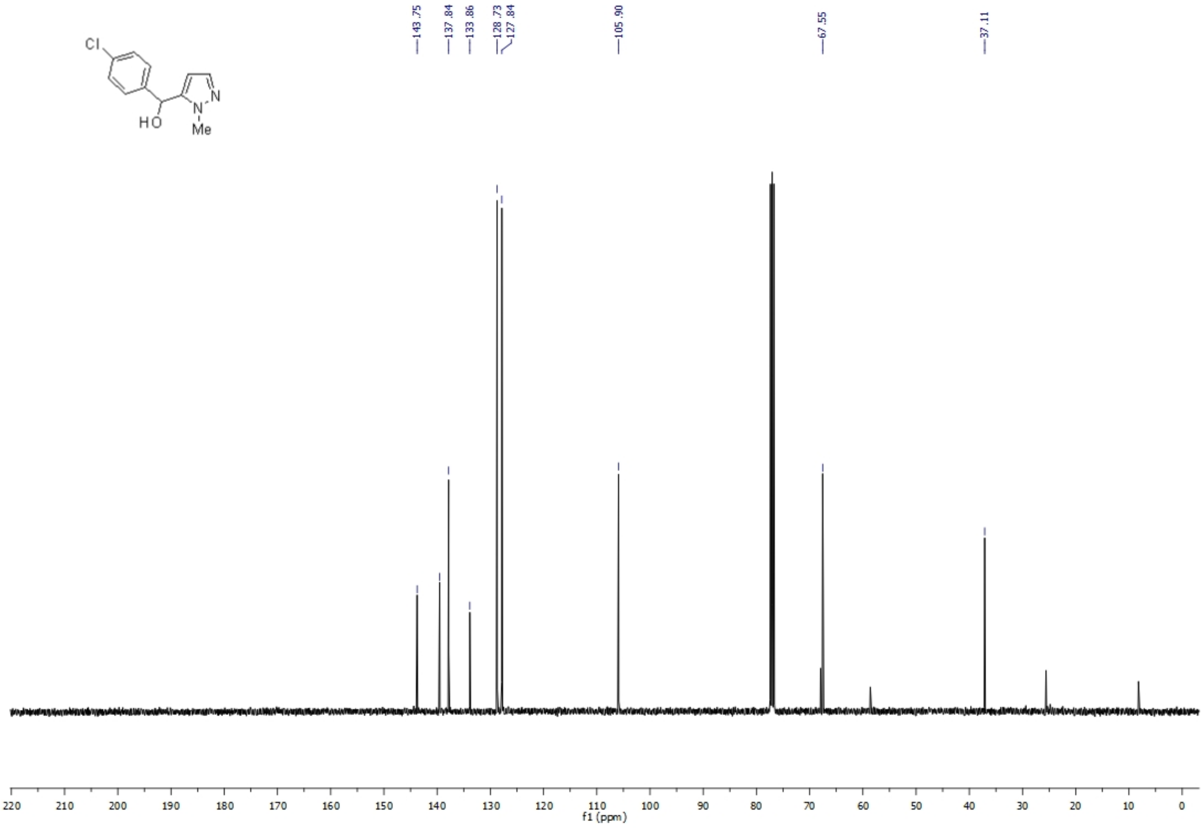

ethyl 5-bromo-3-(cyclohex-2-enyl)furan-2-carboxylate (**5g**)

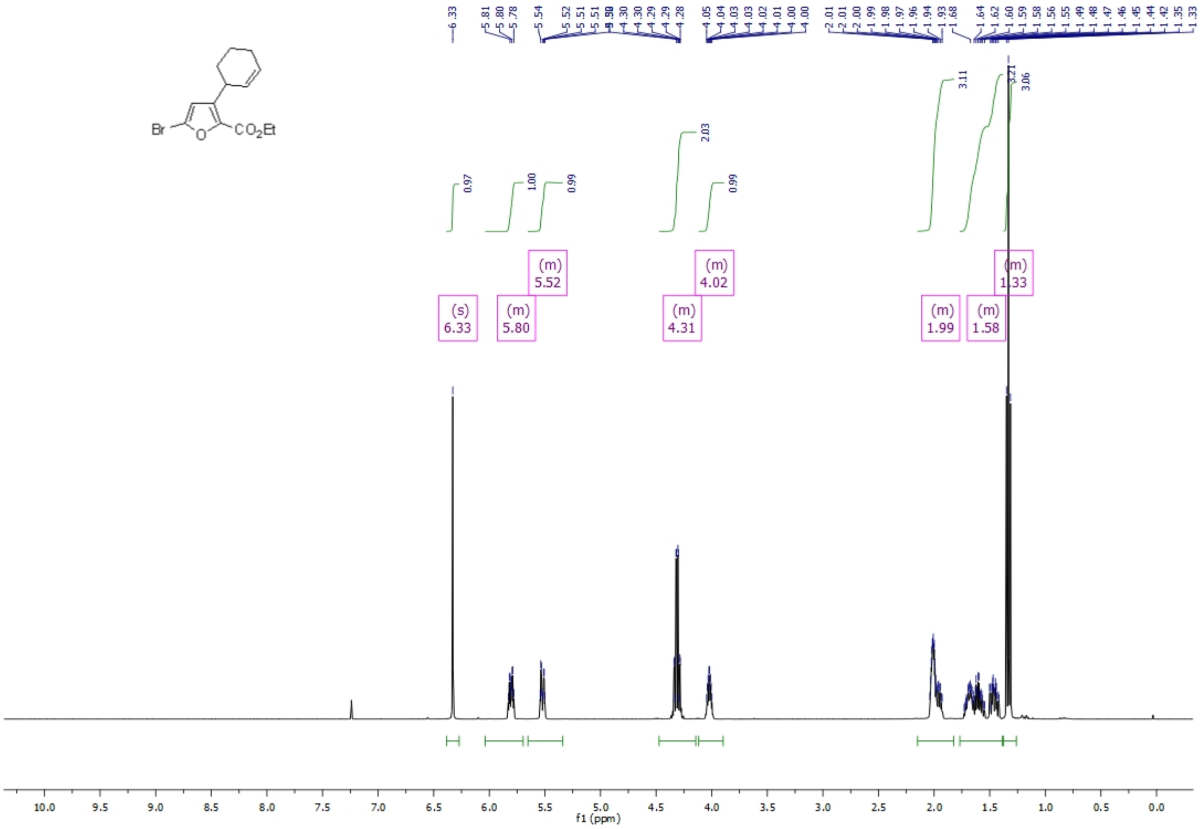


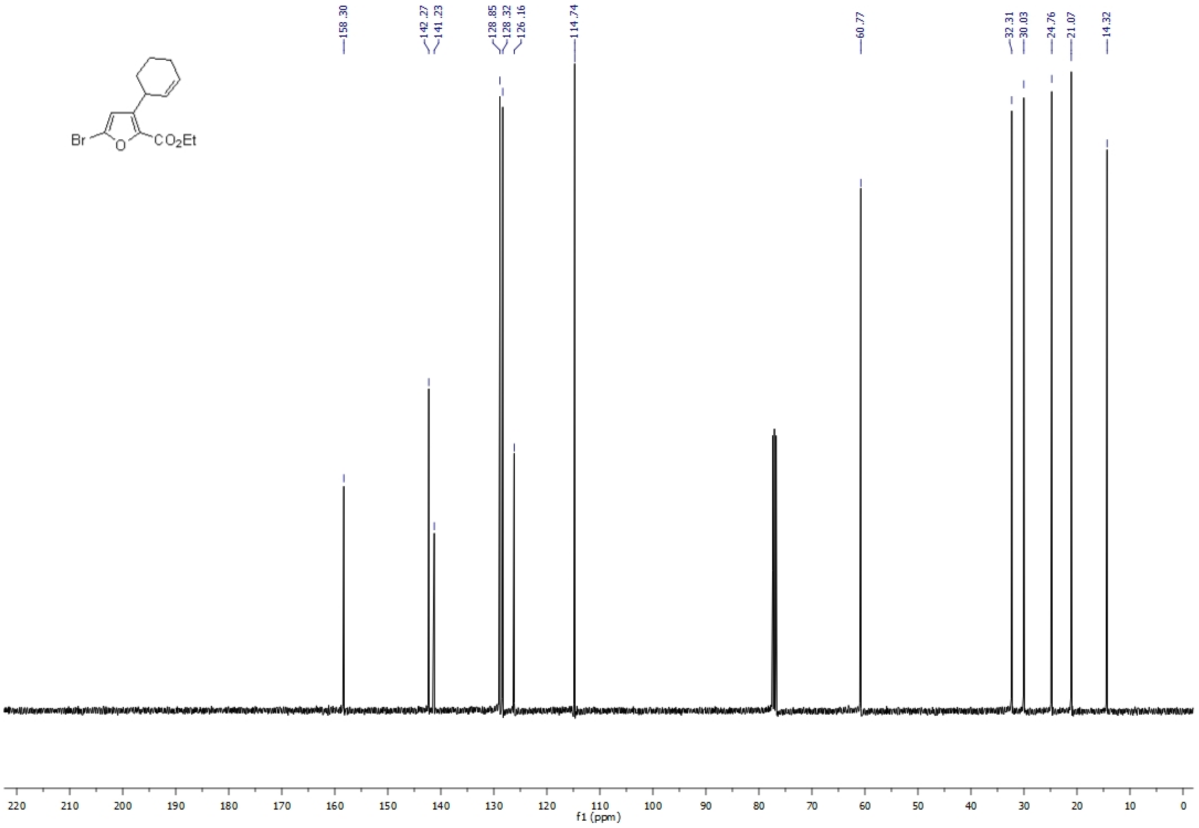

2-bromo-5-(4-(trifluoromethyl)phenyl)thiophene (**5h**)

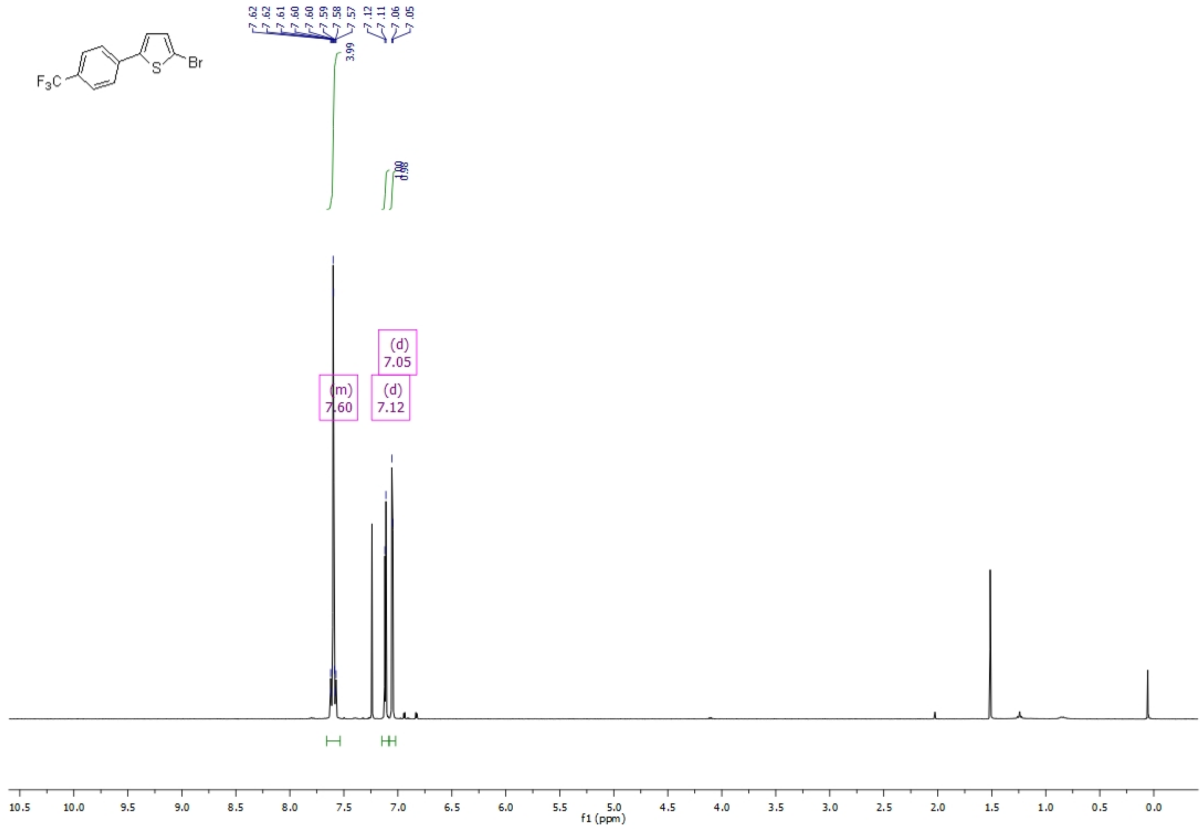


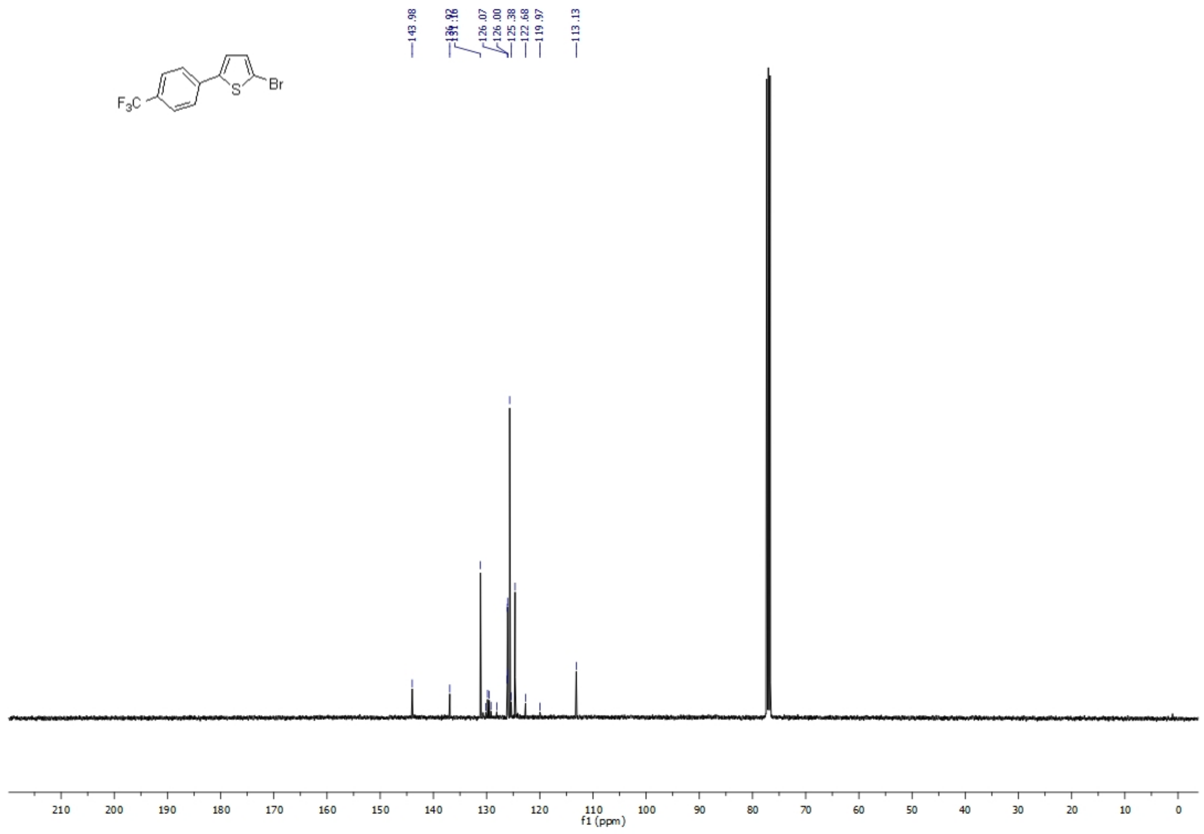

2-bromo-5-(3-nitrophenyl)thiophene (**5i**)

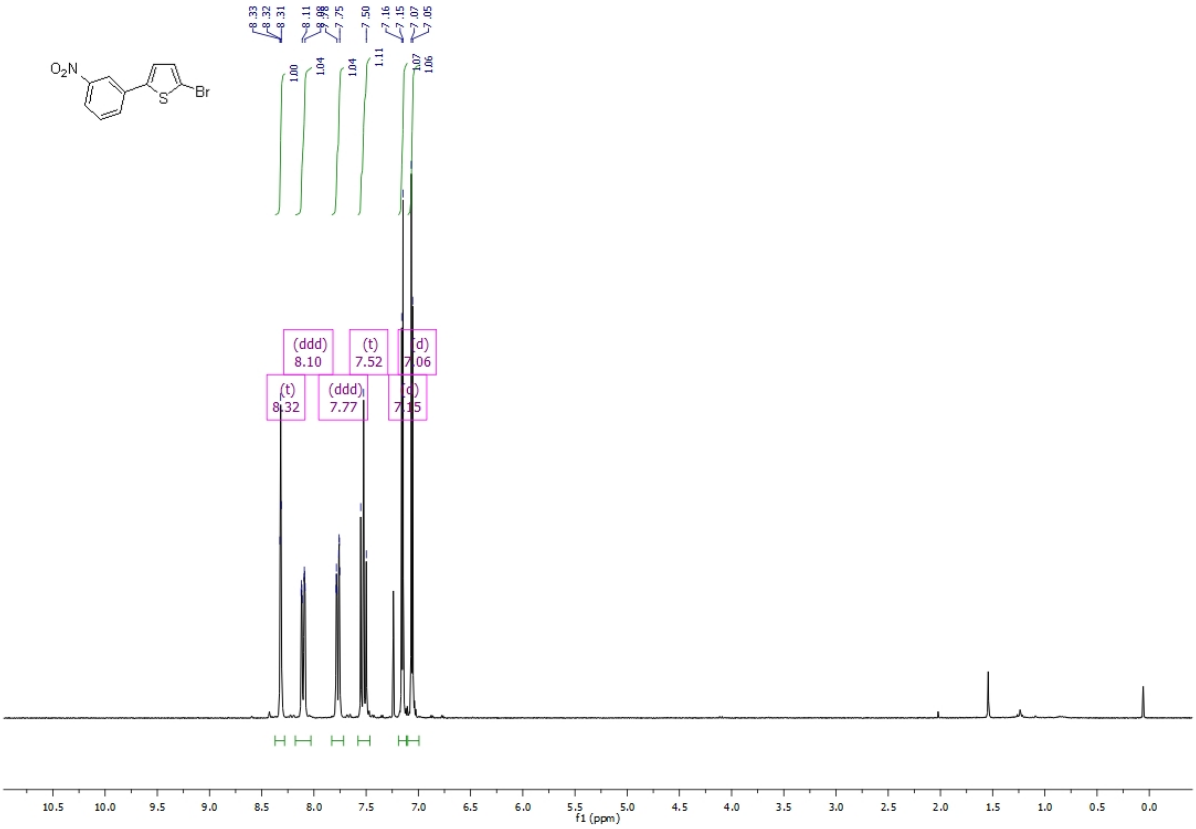


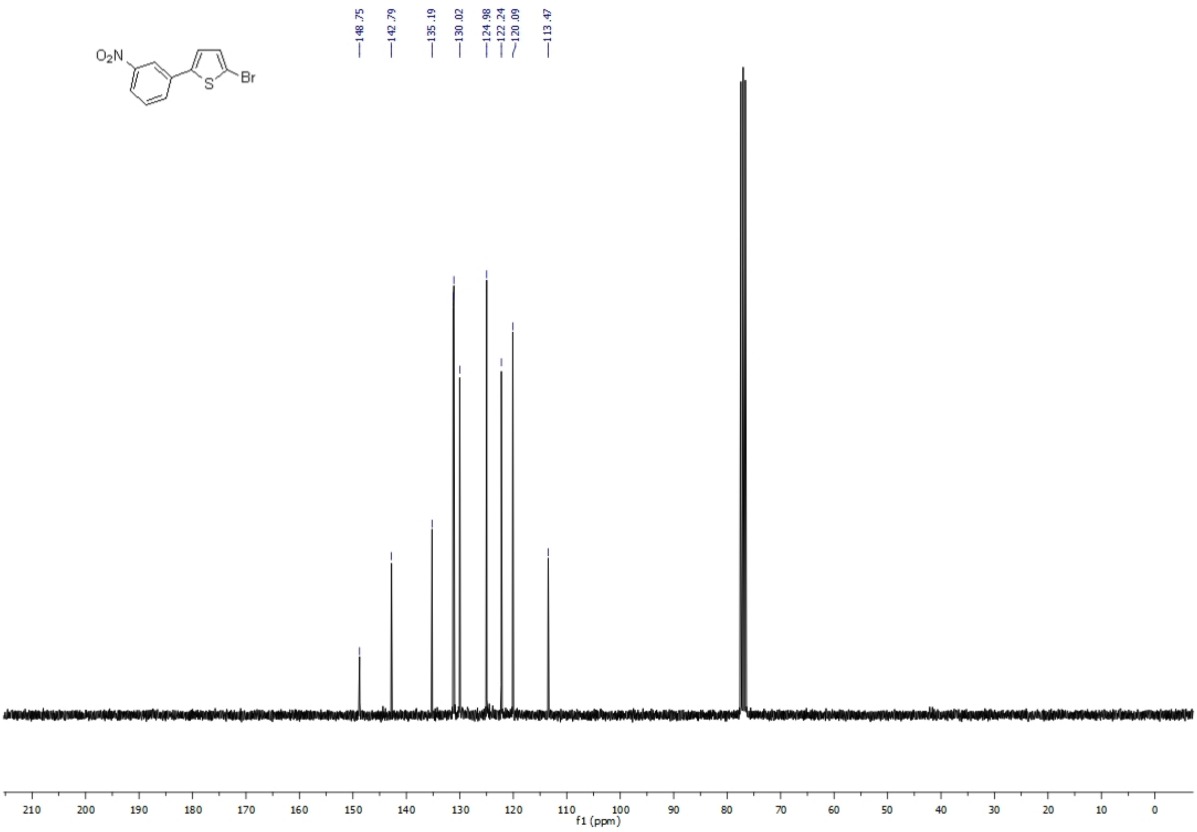

5-(2,6-dichlorophenyl)-4-methoxyfuran-2(5H)-one (**8**)

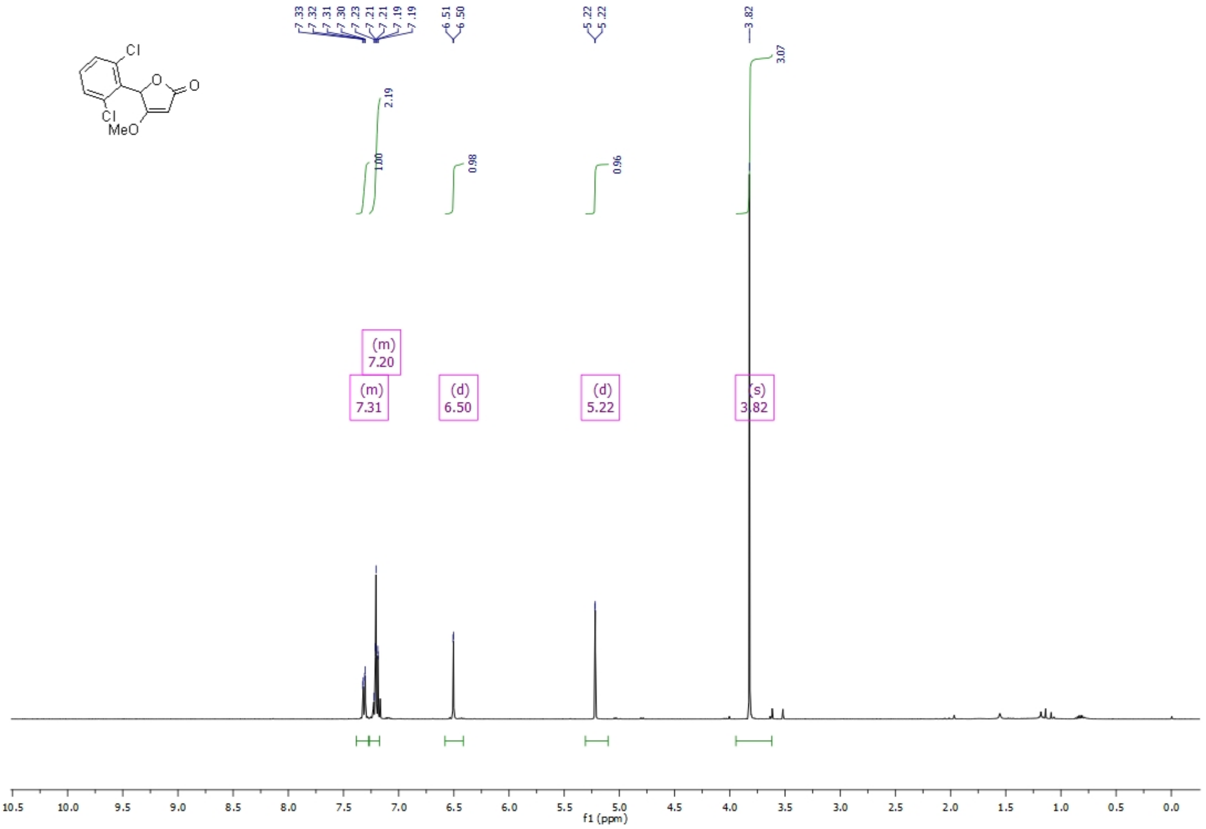


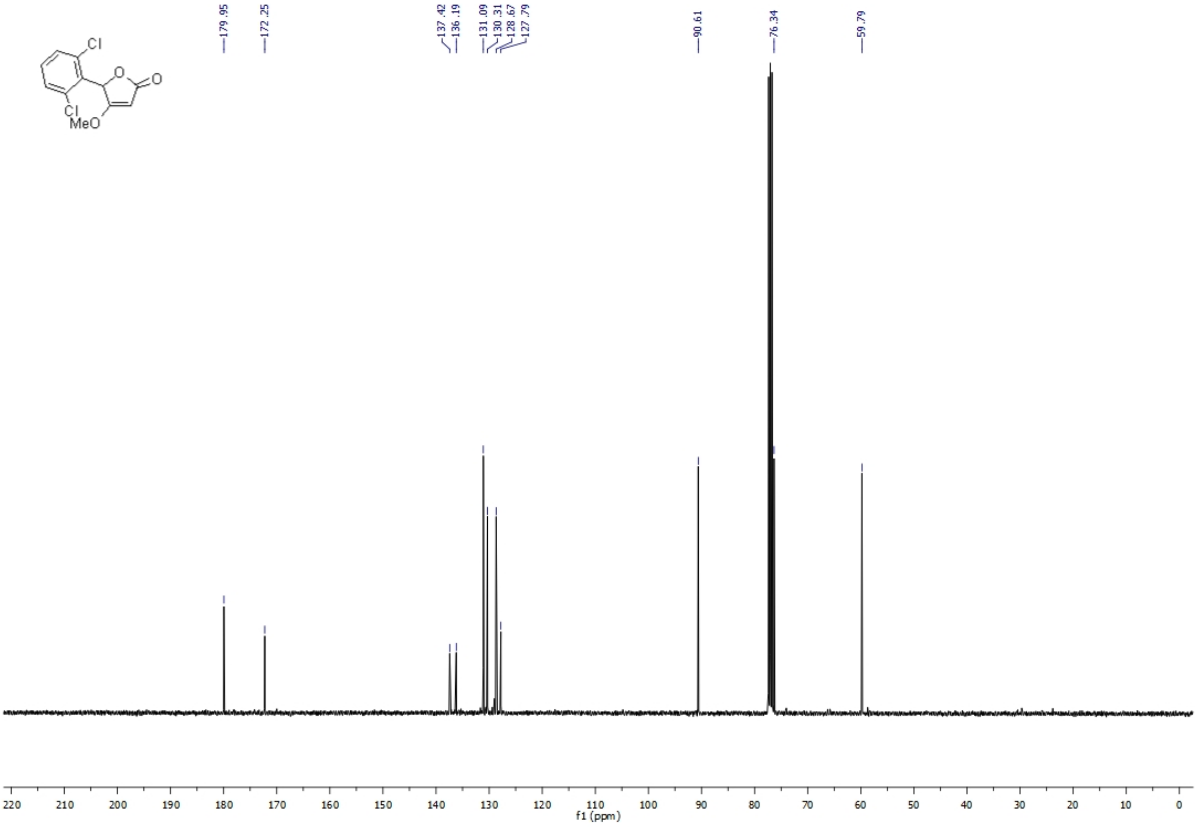

4-(dimethylamino)-5-(4-(trifluoromethyl)phenyl)furan-2(5H)-one (**11**)

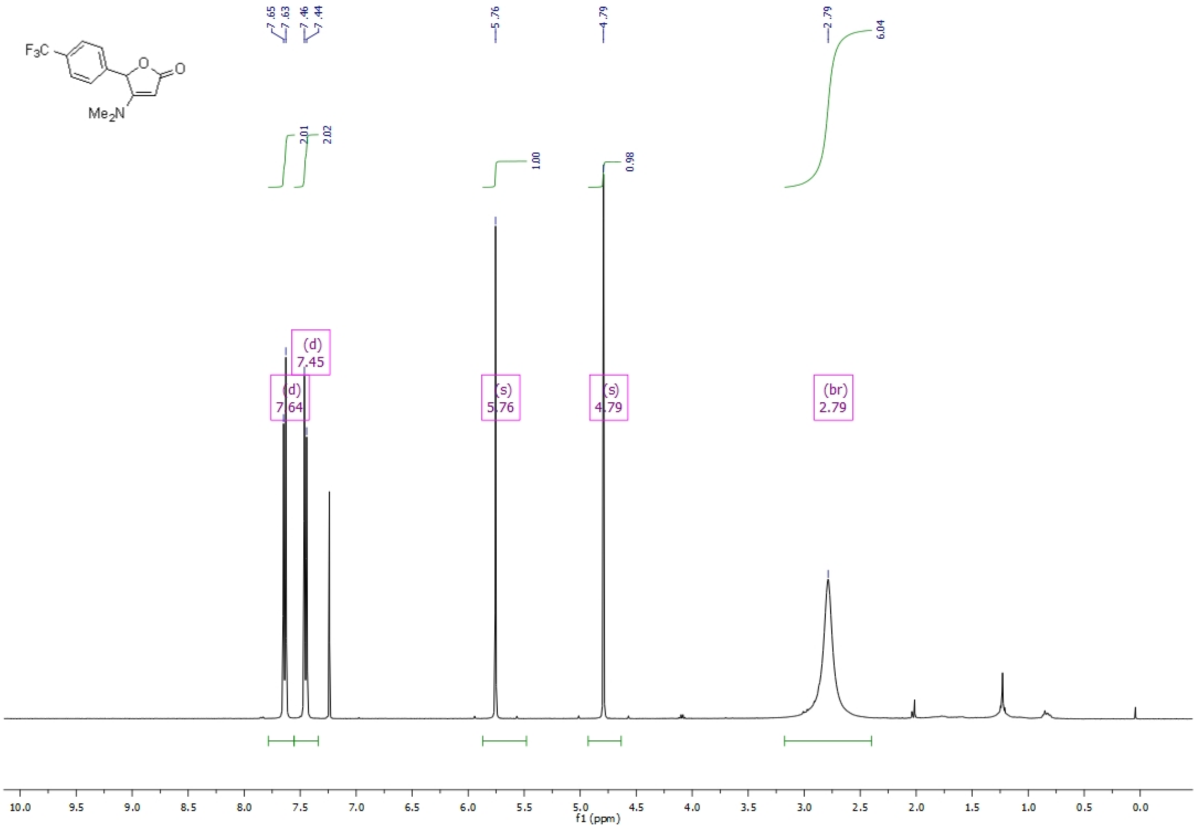


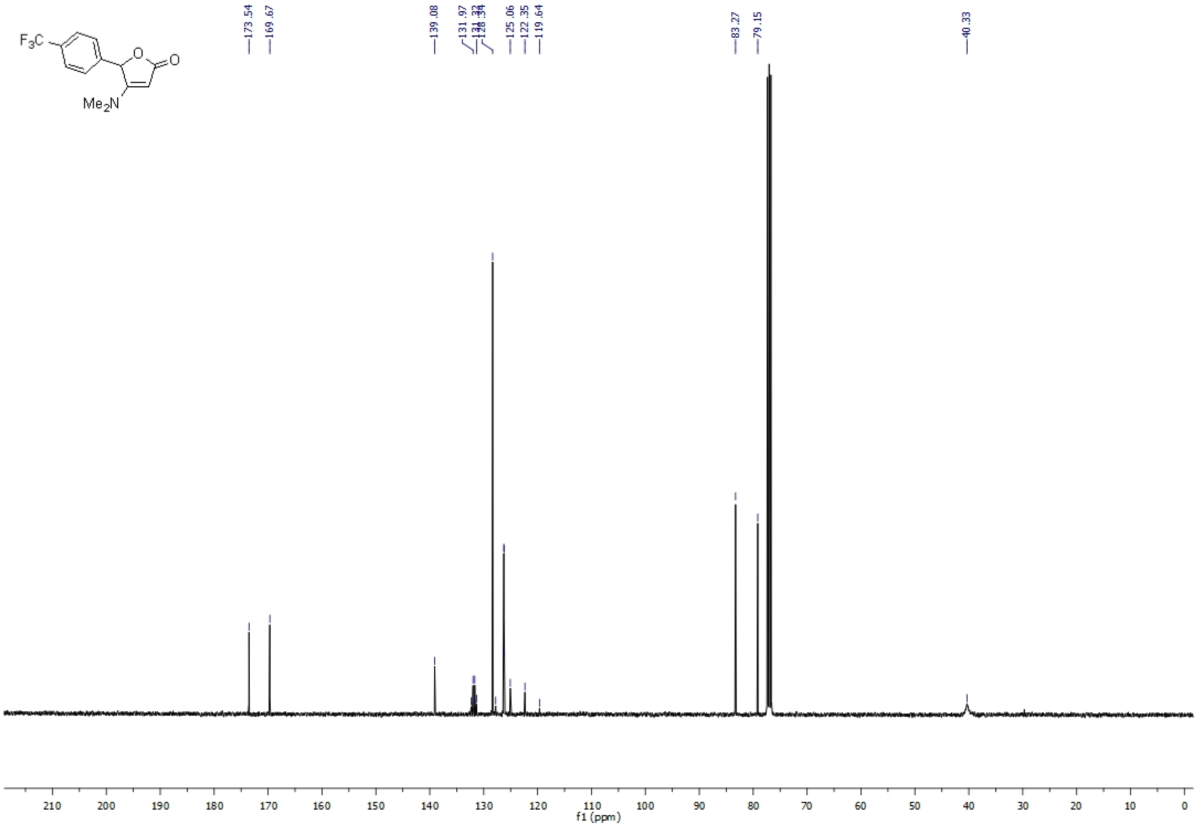

(E)-ethyl 3-(dimethylamino)-3-(4-(trifluoromethyl)phenyl)acrylate (**13**)

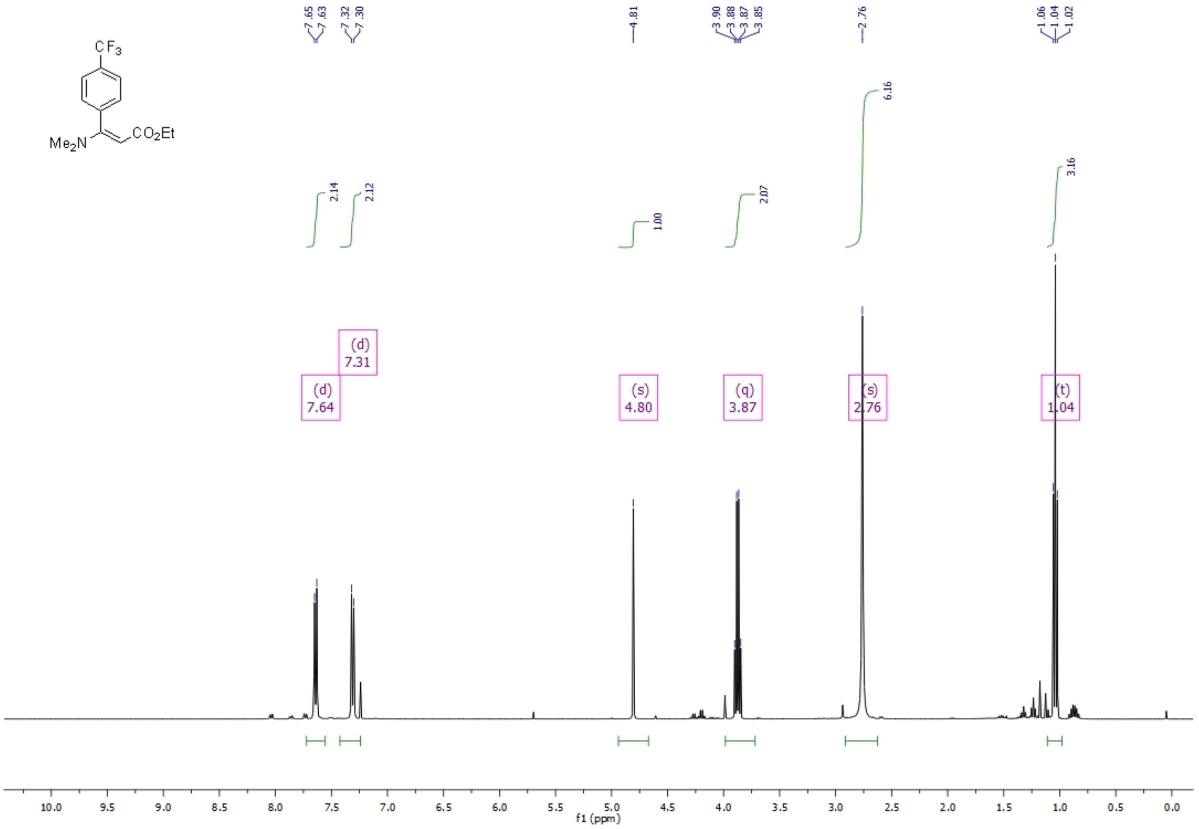


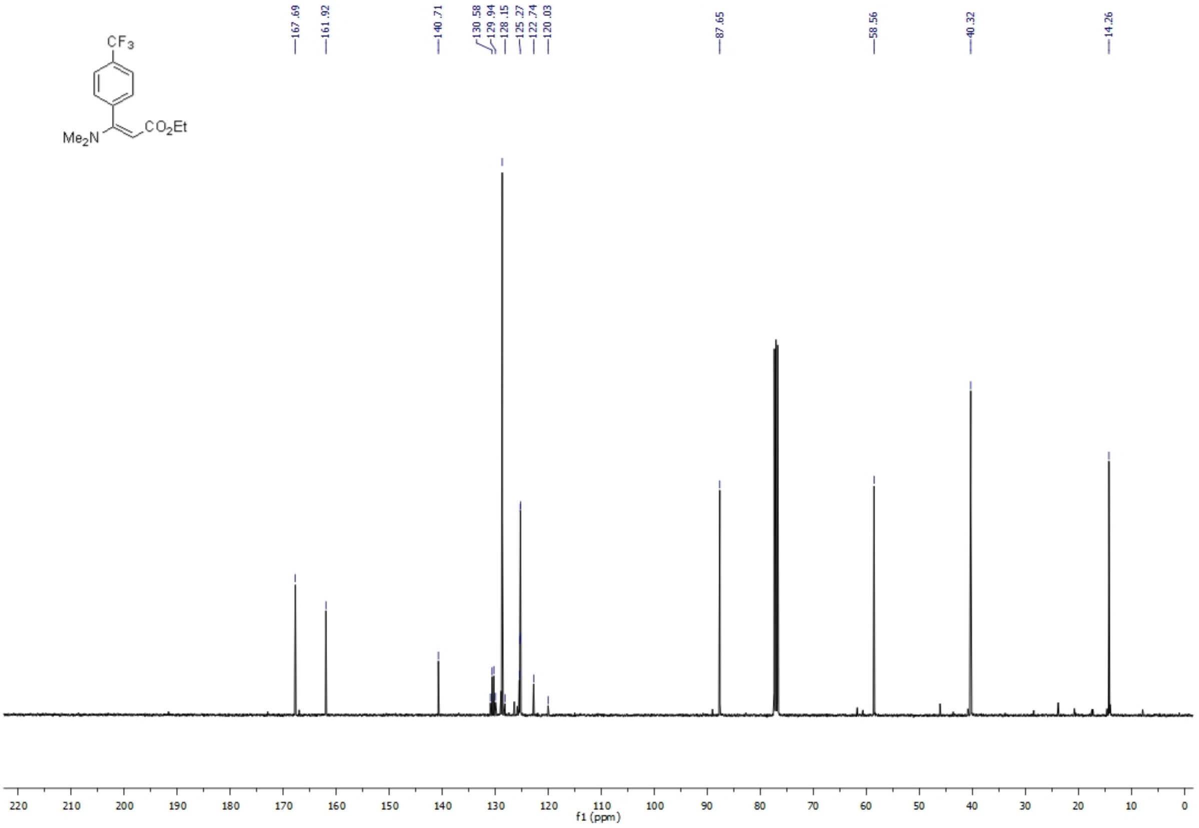

Supplement: Supplementary file 1 [file SC-006-C5SC02558C-s001.pdf]
